# Organocopper(ii) complexes: new catalysts for carbon–carbon bond formation *via* electrochemical atom transfer radical addition (*e*ATRA)†

**DOI:** 10.1039/d2sc03418b

**Published:** 2022-08-17

**Authors:** Miguel A. Gonzálvez, Chuyi Su, Craig M. Williams, Paul V. Bernhardt

**Affiliations:** School of Chemistry and Molecular Biosciences, University of Queensland Brisbane 4072 Australia p.bernhardt@uq.edu.au c.williams3@uq.edu.au

## Abstract

Organocopper(ii) complexes are a rarity while organocopper(i) complexes are commonplace in chemical synthesis. In the course of building a strategy to generate organocopper(ii) species utilizing electrochemistry, a method to form compounds with Cu^II^–C bonds was discovered, that demonstrated remarkably potent reactivity towards different functionalized alkenes under catalytic control. The role of the organocopper(ii) complex is to act as a source of masked radicals (in this case ˙CH_2_CN) that react with an alkene to generate the corresponding γ-halonitrile in good yields through atom transfer radical addition (ATRA) to various alkenes. The organocopper(ii) complexes can be continuously regenerated electrochemically for ATRA (*e*ATRA), which proceeds at room temperature, under low Cu loadings (1–10 mol%) and with the possibility of Cu-catalyst recovery.

## Abstract 2

Electrochemical generation of a novel organocopper(ii) complex offers a new way to carry out atom transfer radical addition to alkenes under mild conditions with high yields and low catalyst loadings.

## Graphical abstract

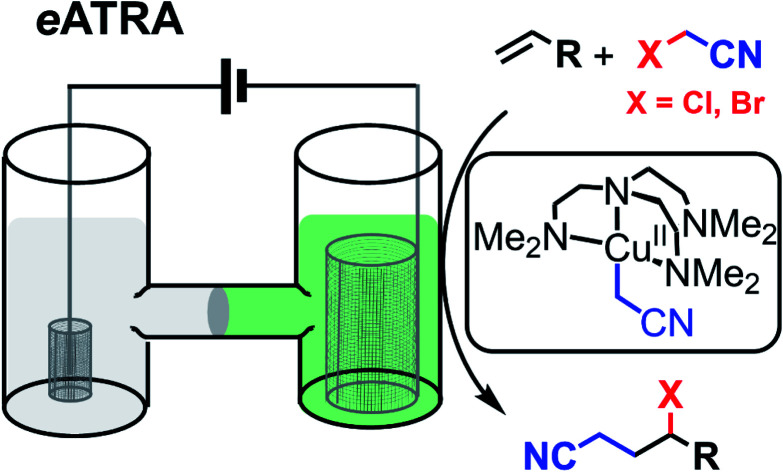

## Introduction

Organocopper(i) compounds are among the most extensively used reagents in the functionalization of organic molecules, namely in the form of nucleophilic C–C bond and C–heteroatom bond formation as stoichiometric reagents or catalysts.^1–4^ In stark contrast to the myriad of organocopper(i) complexes that have been prepared, organocopper(ii) compounds are rare. Only a few organocopper(ii) complexes have been structurally characterized by X-ray diffraction, specifically, those containing ligands that exert sufficient electronic and steric effects to protect the Cu^II^–C bond from dissociation. Examples of these include N-heterocyclic carbene,^5–7^ N-confused porphyrin,^8^ macrocyclic aryl tripyridyl^9^ and tripodal tris(2-pyridylthio)methyl^10^ ligands. Two particularly important cases include monodentate C-bound –CH_2_CN to copper(ii) from the Tolman^11^ and Huang groups,^12^ where pyridine-2,6-dicarboxamide co-ligands were utilized. Huang and co-workers showed that the Cu^II^–CH_2_CN moiety acted as a cyanide source (activating the C–C bond) for catalytic cyanation of iodobenzene, phenylboronic acid, and 2-phenylpyridine. However, beyond these examples, the reactivity of organocopper(ii) complexes remains largely unexplored.

1 [Cu^II^LR]^+^ → [Cu^II^L]^2+^ + R^−^ (heterolytic dissociation),

2 [Cu^II^LR]^+^ → [Cu^I^L]^+^ + R˙ (homolytic dissociation),

A key issue is the reactivity of the Cu^II^–C bond, in terms of both its lability and cleavage mode. As shown in eqn (1), heterolysis of the Cu^II^–C bond generates Cu^II^ and a carbanion (R^−^); a powerful base and nucleophile.^13^ Alternatively, homolysis liberates a radical (R˙) and Cu^I^ (eqn (2)). The latter transformation would render the organocopper(ii) species an ideal candidate for radical addition reactions since a controlled radical release *via* Cu^II^–C bond homolysis minimizes radical termination.

**Scheme 1:**
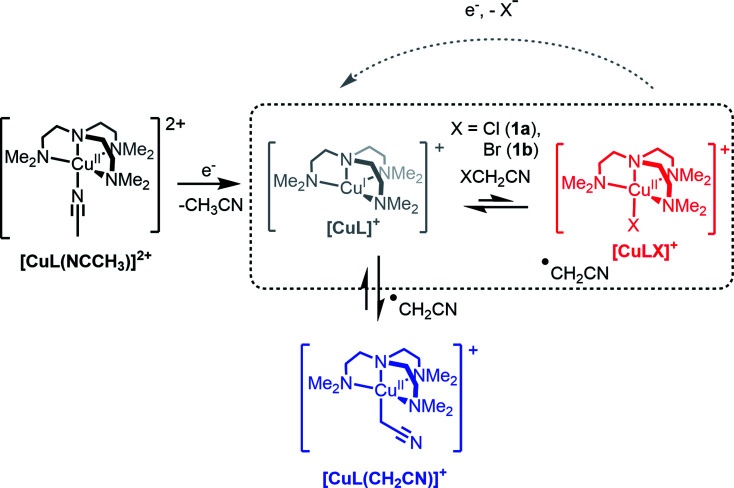
The electrochemically triggered formation of the organocopper(ii) complex [CuL(CH_2_CN)]^+^ (L = Me_6_tren).

The role of Cu complexes in atom transfer radical addition (ATRA) has been well established.^14^ The redox activity of Cu is central to the mechanism of ATRA and the key step is initiation whereby a reactive radical is generated from a dormant alkyl halide. As an illustrative example of initiation (Scheme 1, highlighted box), the Cu(i) complex of the tetradentate ligand Me_6_tren (hereafter abbreviated as L) reacts with an organic halide (XCH_2_CN, X = Cl (1a) or Br (1b)) yielding a halido-copper(ii) complex ([Cu^II^LX]^+^) and the radical ˙CH_2_CN (see Scheme 1). In recently published work, we showed that rapid electrochemical regeneration of [Cu^I^L]^+^ leads to an accumulation of [Cu^I^L]^+^ and ˙CH_2_CN near the electrode, which rapidly combine to form the organocopper(ii) complex [Cu^II^L(CH_2_CN)]^+^.^15–17^

The reactivity of [Cu^II^L(CH_2_CN)]^+^ is now explored in the context of developing and executing controlled carbon–carbon bond formation based on ATRA. One of the main deficiencies of conventional copper-catalyzed ATRA, however, is the need for high Cu loadings relative to the substrate (up to 30%) and high temperatures (over 90 °C) to achieve desired yields and selectivities.^14,18,19^ Electrosynthesis is a promising and innovative synthetic methodological tool in organic synthesis that can accomplish challenging transformations under mild conditions.^20–23^ Herein we report, for the first time, electrochemical atom transfer radical addition (*e*ATRA) with [Cu^II^L(CH_2_CN)]^+^ as the radical source using mild reaction conditions, and with a protocol for catalyst recovery.

## Results and discussion

### Electrochemical synthesis of [Cu^II^L(CH_2_CN)]^+^

In order to generate [Cu^II^L(CH_2_CN)]^+^ in solution, a bulk electrolysis protocol, based on a previously described method, was routinely employed for this work.^15,17^ The stable [Cu^II^L(NCCH_3_)]^2+^ complex forms spontaneously when crystalline [Cu^II^L(OH_2_)](ClO_4_)_2_ (ref. 24) is dissolved in CH_3_CN, and electrochemical reduction to [Cu^I^L]^+^ is accompanied by a change in coordination number (5 to 4), which is typical of copper coordination chemistry.^25^ In the presence of 1a or 1b radical activation occurs generating [Cu^II^LX]^+^ and ˙CH_2_CN (Scheme 1). If the applied electrode potential is kept within a window low enough to reduce [Cu^II^LX]^+^ yet high enough to avoid reduction of [Cu^II^L(CH_2_CN)]^+^ (*E*^0^_[CuLR]_ < *E* < *E*^0^_[CuLX]_), then [Cu^I^L]^+^ and ˙CH_2_CN accumulate and react rapidly^15^ to form [Cu^II^L(CH_2_CN)]^+^ (Scheme 1).

**Fig. 1:**
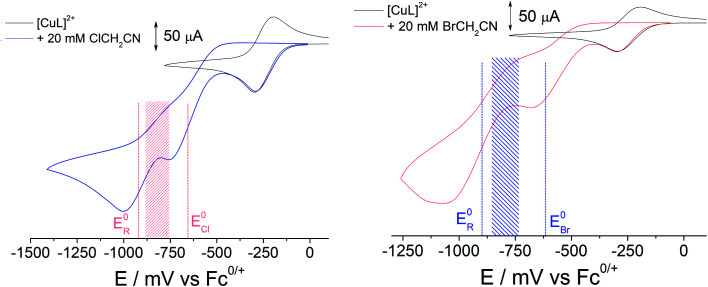
CV of [Cu^II^L(NCCH_3_)]^2+^ (2 mM) before and after the addition of 10 equivalents (20 mM) ClCH_2_CN (left, 1a) and BrCH_2_CN (right, 1b) (at a scan rate of 100 mV s^−1^: highlighted shaded areas show the potential window for forming [Cu^II^L(CH_2_CN)]^+^.

The alkyl halides ClCH_2_CN or BrCH_2_CN can be directly reduced electrochemically^26^ to the radical ˙CH_2_CN (ESI Fig. S3A†), but only at potentials well below those shown in Fig. 1 (<−1600 mV *vs.* Fc^+/0^). The complex [Cu^I^L]^+^ is essential in achieving controlled radical activation. CV experiments carried out with Cu(ClO_4_)_2_ in CH_3_CN (giving the [Cu(NCCH_3_)_4_]^2+/+^ couple at *ca.* +650 mV *vs.* Fc^+/0^) led to no catalytic reaction with either ClCH_2_CN or BrCH_2_CN upon electrochemical reduction (ESI Fig. S3B and C†). This is in line with the known dependence of the radical activation rate constant on the Cu^II/I^ redox potential.^27^

### Stoichiometric ATRA

When electrogenerated [Cu^II^L(CH_2_CN)]^+^ and styrene (2) were mixed anaerobically in equimolar quantities for 24 h at room temperature, a polymeric precipitate formed with no detectable quantities of the desired ATRA product by ^1^H NMR analysis (Table 1, entry 5). When 2 equivalents of the halide 1b were added to the reaction mixture, the desired γ-halonitrile (2b) was obtained in 25% yield (Table 1, entry 1). The reaction was optimized for temperature and reaction time (Table 1, entries 1–4) leading to higher product yields upon heating, but at temperatures above 60 °C elimination of HBr from 2b gave the corresponding alkene 2c (Table 1, entry 3). Similarly, increased amounts of 1b relative to [Cu^II^L(CH_2_CN)]^+^ and 2 led to higher yields of 2b at lower temperatures (Table 1, entries 6 and 7), but increasing amounts of succinonitrile (1d) were observed to form through self-termination (dimerization) of ˙CH_2_CN radicals. Reaction at 82 °C (refluxing acetonitrile) gave no product due to the thermal polymerisation of 2.^28^ Importantly, no elimination occurred when 1a was used under the same conditions given the greater stability of the chlorinated product 2a.

**Table 1:** Optimization of the stoichiometric reaction of [CuL(CH_2_CN)]^+^ with styrene (2) at different temperatures and equivalents of BrCH_2_CN (1b)^a^

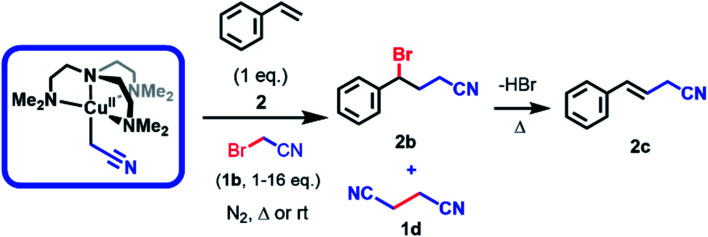					
Entry	Ratio 2 : 1b	Temperature (°C)	Monoadduct formation^b^ (%)	Ratio^b^	
				2b	2c
1	1 : 2	25	25	100	0
2	1 : 2	40	55	100	0
3	1 : 2	60	100	75	25
4	1 : 2	82 (reflux)	0	—	—
5	1 : 1	60	0	—	—
6	1 : 6	60	100	94	6
7	1 : 16	60	100	94	6

a Reactions were carried out with the electro-generated [Cu^II^L(CH_2_CN)]^+^ (*E*_app_ −860 mV *vs.* Fc^+/0^) in 25 mL of anhydrous CH_3_CN (0.1 M [Et_4_N](ClO_4_)) under N_2_ for 24 h. The ratio of styrene (2) and [Cu^II^L(CH_2_CN)]^+^ was 1 : 1. Reactions were monitored by TLC and ^1^H NMR spectroscopy.

b Determined by ^1^H NMR (CDCl_3_) and expressed as a percentage of the styrene derivatives 2b and 2c.

### Electrocatalytic ATRA (*e*ATRA)

Gratifyingly, the same reaction outcome could be achieved at room temperature under electrocatalytic conditions with sub-stoichiometric amounts of copper complex in the presence of 2 and two equivalents of 1a or 1b. This led to the formation of the ATRA adducts (2a and 2b) in good yields at room temperature with a significant decrease in reaction time.

The effect of pre-catalyst [Cu^II^L(NCCH_3_)]^2+^ concentration was examined by investigating the room temperature electrochemical ATRA reaction of 1a and 2. High conversions and good yields were obtained when using 1–10 mol% of the pre-catalyst with reaction times under 12 h (Table 2, entries 1–4). When catalyst loadings decreased to 0.4 mol% or less, longer reaction times were required and lower yields were obtained (Table 2, entries 5–6). Loadings over 10 mol% Cu did not shorten reaction times or improve yields so all subsequent experiments were carried out with 10 mol% Cu loading.

**Table 2:** Optimization of pre-catalyst loadings ([Cu^II^L(NCCH_3_)](ClO_4_)_2_) for *e*ATRA reaction of styrene (2) and ClCH_2_CN (1b)^a^

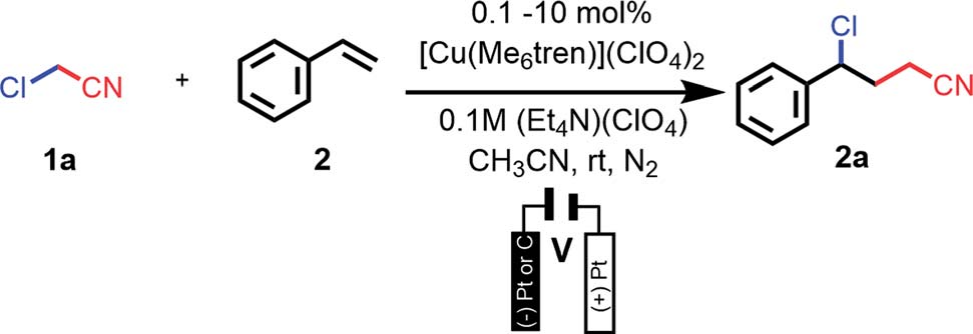			
Entry	Loading of [Cu^II^L(NCCH_3_)](ClO_4_)_2_ (mol%)	Conversion^b^ (%)	Yield^c^ (%)
1	10	94	72
2	5	100	60
3	2	83	64
4	1	75	46
5	0.4	82	37
6	0.2	27	12

a All reactions were performed with 100 mg (0.96 mmol) of styrene (2) at room temperature in an H-cell under N_2_, and the molar ratio of 1a : 2 was 1 : 2. The applied working electrode potential was −960 mV *vs.* Fc^+/0^. Reactions were generally complete within 5 h, except for entry 6, which required *ca.* 16 h.

b Based on ^1^H NMR.

c Isolated product after chromatography.

### *e*ATRA scope

**Scheme 2:**
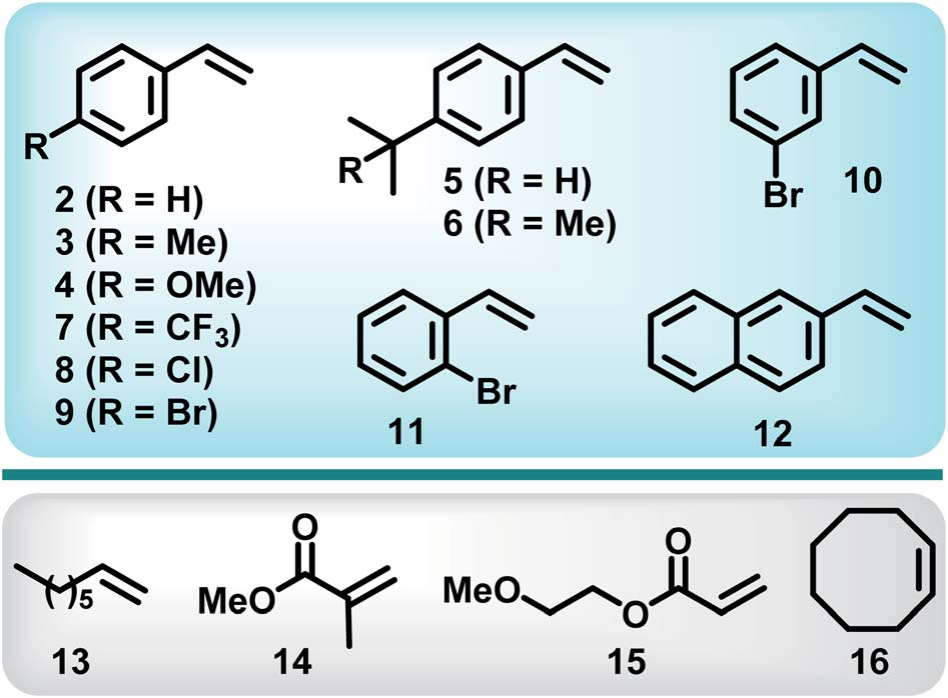
Alkene substrates investigated with *e*ATRA.

**Scheme 3:**
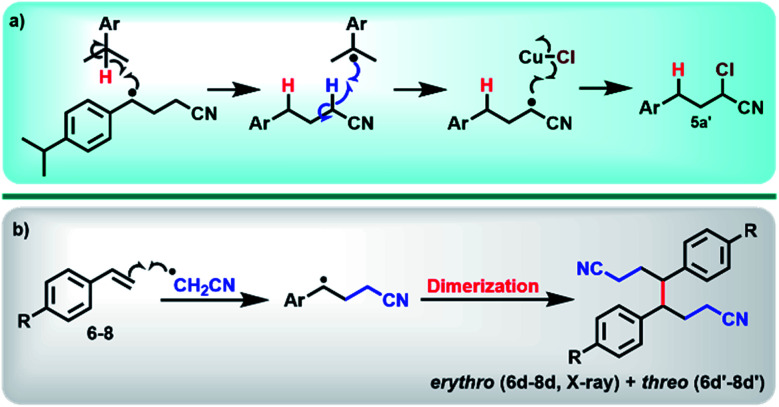
(a) Proposed intermolecular chain transfer mechanism of the radical intermediate formed in the reaction of *para*-isopropyl styrene (5) to form the isomeric by-product 5a′. (b) Termination of the transient radical intermediate in the absence of [Cu^II^LBr]^+^, which leads to the formation of dimers (6d–8d/6d′–8d′).

With optimum conditions determined, the scope of the copper-catalyzed *e*ATRA was investigated by employing various functionalized alkenes (2–16, Scheme 2) to react with organic halides 1a or 1b (Table 3). *Para-*substituted styrenes afforded the expected ATRA γ-halonitrile products (*i.e.*, 2–9a, 2–5b, 9b) in moderate to excellent yields (52–96%) with no alkene elimination by-products (*e.g.*2c). However, *p*-isopropylstyrene (5), also gave a small amount of isomeric halonitrile by-product 5a′. This is potentially due to an intermolecular radical chain transfer mediated by the reactive Me_2_CH– substituent (Scheme 3a). Non-aromatic alkenes (13–16), exhibit full conversion to the corresponding ATRA products by ^1^H NMR analysis. Volatility of these aliphatic products is the origin of their lower isolated yields. Of the two organic halides surveyed, 1b required shorter reaction times compared with 1a, which was in accord with the expected relative C–Br and C–Cl bond reactivity (strength). Despite this, the yields were consistently higher when 1a was employed, so 1a became the focus for *e*ATRA while 1b was limited to representative examples from Scheme 2. The results are summarized in Table 3.

**Table 3:** Substrate scope of *e*ATRA reaction utilizing functionalized alkenes (2–16) in the formation of γ-halonitriles^a^

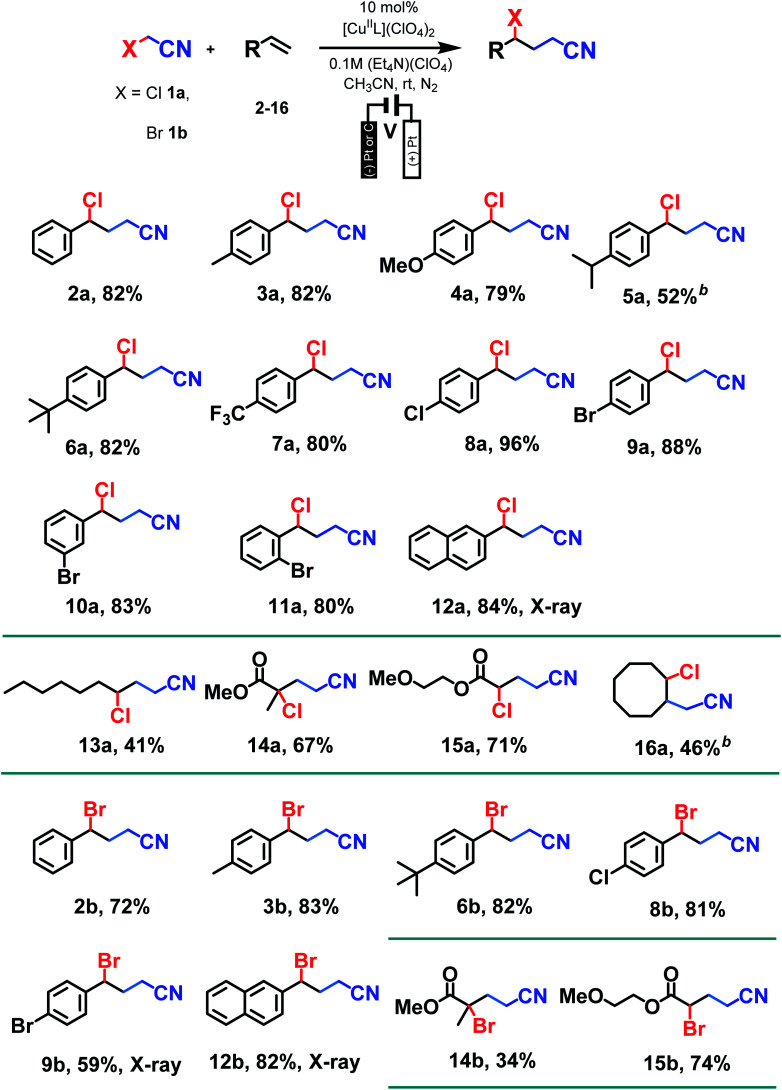

a Reactions undertaken with [Cu^II^L(NCCH_3_)^2+^(10 mol%) in anhydrous CH_3_CN (50 mL, 0.1 M (Et_4_N)(ClO_4_)) under N_2_ and at 298 K. Yields of isolated product shown unless noted otherwise.

b Yield corresponds to total isomeric product mixture (5a + 5a′ or *syn* + *anti*16a).

When *e*ATRA reactions of *p-t*-butylstyrene (6), *p*-trifluoromethylstyrene (7), and *p*-chloro styrene (8) were explored at very low catalyst loadings (*e.g.*, 10 mM alkene and 0.01 mM [Cu^II^L(NCCH_3_)](ClO_4_)_2_ (0.1 mol%)), the corresponding dimers (6d/6d′, 7d/7d′ and 8d/8d′) were formed as mixtures of *erythro*- and *threo*-isomers. The centrosymmetric *erythro*-isomers were all characterised by X-ray crystallography (see ESI†). These products are a result of termination of the transient radical intermediate following radical addition (Scheme 3b), when insufficient [Cu^II^LX]^+^ is present to complete ATRA by halogen atom transfer. To avoid this reaction, the loadings of the copper pre-catalyst should be kept above 1 mol% relative to alkene substrates.

### Mechanism

**Scheme 4:**
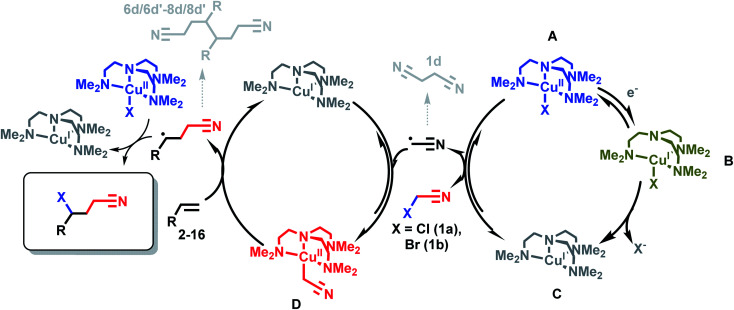
Reaction scheme for copper-catalyzed *e*ATRA. Charges omitted for clarity.

Scheme 4 illustrates the roles of each Cu complex (A–D) in the *e*ATRA mechanism. Electrochemical reduction of [Cu^II^LX]^+^ (A) to [Cu^I^L]^+^ (C) *via* the halido cuprous complex [Cu^I^LX] (B) initiates the cycle. The role of [Cu^II^L(CH_2_CN)]^+^ (D) in *e*ATRA is to stabilise ˙CH_2_CN and block self-termination (to 1d). The complex [Cu^II^L(CH_2_CN)]^+^ has proven to be a reactive yet robust intermediate that we have been able to prepare *in situ* and characterise spectroscopically.^15,17^ However, the halido complex [Cu^II^LX]^+^ (X = Cl, Br) (A) is an equally essential participant in *e*ATRA as a halogen atom donor to form the final product and close the catalytic cycle (Scheme 4, left hand side). Without [Cu^II^LX]^+^ (generated by the second equivalent of XCH_2_CN), dimers (6d/6d′–8d/8d′) or polymeric products ensue. As illustrated in Scheme 4, this reaction is genuinely catalytic as no Cu complex is consumed; only the first electron to reduce the initial [CuL(NCCH_3_)]^2+^ pre-catalyst is required.

## Conclusions

Electrochemically mediated atom transfer radical addition (*e*ATRA), is enabled by a rare but resilient organocopper(ii) species [Cu^II^L(CH_2_CN)]^+^ (L = Me_6_tren), generating new carbon–carbon bonds in good to excellent yields under mild reaction conditions. The complex [Cu^II^L(CH_2_CN)]^+^ is a controlled source of ˙CH_2_CN radicals that add to aromatic and aliphatic alkenes (2–16) either stoichiometrically or catalytically (1–10% mol Cu), and importantly the pre-catalyst can be easily recovered after work-up.

## Data availability

All experimental data are provided in the ESI.†

## Author contributions

M. A. Gonzálvez and C. Su carried out all experimental work and contributed equally. All authors analysed the data and contributed to writing the manuscript.

## Conflicts of interest

There are no conflicts to declare.

## Supplementary Material

SC-013-D2SC03418B-s001

SC-013-D2SC03418B-s002
