# Supplementary material for: Organocopper(ii) complexes: new catalysts for carbon–carbon bond formation via electrochemical atom transfer radical addition (eATRA)
Source: Chem Sci. 2022 Aug 17;13(35):10506–11. doi: 10.1039/d2sc03418b (PMC9473645; doi:10.1039/d2sc03418b)
Supplement: SC-013-D2SC03418B-s001 [file SC-013-D2SC03418B-s001.pdf]

Electronic Supplementary Material (ESI) for Chemical Science.
This journal is © The Royal Society of Chemistry 2022

Supporting Information

**1.**	**General Information**	**2**
**2.**	**Synthesis and Characterization of Substrates**	**3**
**3.**	**Electrochemical Procedures**	**5**
**4.**	**Stoichiometric ATRA of [Cu(Me_6tren)(CH_2CN)]$^+$ with styrene**	**7**
**5.**	**Optimized protocol for eATRA**	**8**
**6.**	**Reaction conditions for the electro-synthesis of dimeric products**	**9**
**7.**	**Characterization Data of Products**	**9**
**8.**	**NMR Spectra**	**20**
**9.**	**Control Electrochemical Experiments**	**89**
**10.**	**X-Ray Crystal Structures**	**90**
**11.**	**References**	**102**

1. General Information

Caution. Perchlorate salts are potentially explosive, and special care should be taken when handling them. They should never be heated in the solid state or scraped from sintered glass frits.

Characterization Methods

^{1}H- and ^{13}C-NMR spectra were recorded with a Bruker AS500 (500 MHz, 125 MHz), AV500 (500 MHz, 125 MHz) spectrometer at 25 °C in $CDCl_3$. The chemical shift (δ) is given in parts per million (ppm), and the signal multiplicities are abbreviated as follows: s = singlet, d = doublet, dd = doublet of doublets, t = triplet, dt = doublet of triplets, m = multiplet.

High-resolution mass spectrometry (HRMS) was conducted using a Bruker MicroOTOF-Q spectrometer HCT 3D Ion Trap spectrometer, and low-resolution mass spectrometry (LRMS) measurements were conducted in a Bruker HCT 3D Ion Trap spectrometer. GCMS experiments were performed with a Shimadzu QP2010-Ultra GC-MS Instrument equipped with a Restek Rtx-5MS (Integra Guard) capillary column (30 m × 0.25 mm × 0.1 µm). A 1 µL aliquot of the sample dissolved in CH_2Cl_2 was injected by split injection mode with an initial pressure of 72.6 kPa and a flow rate of 1 mL min^{-1}. The injector temperature was set at 200 °C, and the column oven temperature at 100 °C for 1 min, ramping to 275 °C at 15 °C min^{-1} during 28.5 min. The ion source temperature was set at 230 °C, and the data were collected in the time interval 2.6-45.2 min. Melting point measurements were conducted in a capillary tube using a Digimelt MPA160 melting point apparatus.

Crystallographic data were acquired on an Oxford Diffraction Gemini dual source (Mo/Cu) diffractometer with the crystals cooled to 190 K with an Oxford Cryosystems Desktop Cooler. Data reduction was performed with the CrysAlisPro software. Structures were solved with SHELXT and refined with SHELXL.[1] Thermal ellipsoid diagrams were generated with Mercury.[2] All calculations were carried out within the WinGX user interface.[3] Crystallographic data in CIF format have been deposited (CCDC deposition numbers 2176281-2176286).

Reagents and Materials

The alkenes styrene (**2**), 4-methylstyrene (**3**), 4-tert-butylstyrene (**6**), 4-trifluoromethylstyrene (**7**) and 4-chlorostyrene (**8**) were purchased from commercially available

sources. Before use, the stabilizers present in the commercial alkene samples were removed by distillation. Acetonitrile and THF were freshly distilled from CaH_2 and sodium/benzophenone, respectively. All moisture-sensitive reactions were performed with oven-dried glassware. $(Et_4N)(ClO_4)$ was prepared, and purified, as previously described.[4]

Styrenes (**4-5**, **9-11**) and 2-vinylnaphthalene (**12**) were synthesized following a procedure based on Haubenreisser *et al.*[5] *n*-BuLi (1.38 M in hexane, 1.3 eq) was added dropwise to a suspension of methyltriphenylphosphonium bromide ($MePPh_3Br$, 2.0 eq) in anhydrous THF under an argon atmosphere at 0°C. The reaction mixture was allowed to warm to room temperature over an hour before the addition of the neat aldehyde (1.0 eq). Alternatively, a solution of aldehyde (1.0 eq) in anhydrous THF (5 mL) was slowly added to the reaction mixture at 0°C. After stirring at room temperature overnight, the reaction was quenched with sat. aqueous NH_4Cl (15 mL) and extracted with EtOAc (2 × 20 mL). The aqueous layer was washed with EtOAc (3 × 20 mL). The combined organic layers were further washed with brine (3 × 30 mL), dried over Na_2SO_4, filtered, and concentrated under a stream of N_2.

2. Synthesis and Characterization of Substrates

4-Vinylanisole (4)

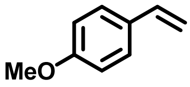

Freshly distilled 4-anisaldehyde (0.40 mL, 3.29 mmol, 1.0 eq), $MePPh_3Br$ (2.36 g, 6.58 mmol, 2.0 eq) and 1.38 M *n*-BuLi solution in hexane (3.1 mL, 4.29 mmol, 1.3 eq) were used in 20 mL of anhydrous THF. The residue was purified by column chromatography (Silica gel, 2%-10% EtOAc in pet. ether) to provide **4** as a colorless oil (331 mg, 71%). ^{1}H-NMR (500 MHz, $CDCl_3$): δ (ppm) 7.36-7.35 (d, *J* = 8.9 Hz, 2H), 6.88-6.85 (d, *J* = 11.6 Hz, 2H), 6.70-6.64 (dd, *J* = 17.6, 10.9 Hz, 1H), 5.63-5.60 (dd, *J* = 17.6, 1.0 Hz, 1H), 5.14-5.12 (dd, *J* = 10.9, 1.0 Hz, 1H), 3.82 (s, 3H). ^{13}C-NMR (125 MHz, $CDCl_3$): δ (ppm) 159.5, 136.4, 130.6, 127.5, 114.1, 112.2, 55.4. LRMS (ESI) $[M+H]^+$: 135.00. Spectroscopic data matched the literature.[6]

4-Isopropyl styrene (5)

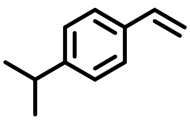

Freshly distilled cuminaldehyde (1.0 mL, 6.55 mmol, 1.0 eq), $MePPh_3Br$ (4.68 g, 13.10 mmol, 2.0 eq) and 1.38 M *n*-BuLi solution in hexane (6.17 mL, 8.52 mmol, 1.3 eq) were used in 30 mL of anhydrous THF. The residue was purified

by column chromatography (Silica gel, 100% pet. ether) to provide **5** as a pale-yellow oil (356 mg, 37%). ^{1}H-NMR (500 MHz, $CDCl_3$): δ (ppm) 7.36-7.34 (d, *J* = 8.0 Hz, 2H), 7.20-7.19 (d, *J* = 8.5 Hz, 2H), 6.73-6.67 (dd, 17.6, 10.8 Hz, 1H), 5.72-5.69 (dd, *J* = 17.6, 1.0 Hz, 1H), 5.20-5.18 (dd, *J* = 10.9, 1.0 Hz, 1H), 2.93-2.87 (m, 1H), 1.26-1.24 (d, *J* = 7.0 Hz, 6H). ^{13}C-NMR (125 MHz, $CDCl_3$): δ (ppm) 148.8, 136.9, 135.4, 126.7, 236.3, 113.0, 34.0, 24.1. LRMS (ESI) $[M+H]^+$: 147.08. Spectroscopic data matched the literature.[7]

4-Bromostyrene (9)

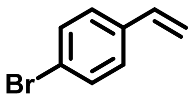

Freshly distilled 4-bromobenzaldehyde (0.70 g, 3.78 mmol, 1.0 eq), $MePPh_3Br$ (2.12 g, 7.56 mmol, 2.0 eq) and 1.38 M *n*-BuLi solution in hexane (3.56 mL, 4.92 mmol, 1.3 eq) were used in 30 mL of anhydrous THF. The residue was purified by column chromatography (Silica gel, 100% pet. ether) to provide **9** as a yellow oil (370 mg, 54%). ^{1}H-NMR (500 MHz, $CDCl_3$): δ (ppm) 7.46-7.44 (d, *J* = 8.5 Hz, 1H), 7.29-7.27 (d, *J* = 8.9 Hz, 2H), 6.68-6.63 (dd, *J* = 17.6, 10.9 Hz, 1H), 5.76-5.73 (dd, *J* = 17.6, 0.7 Hz, 1H), 5.29-5.27 (dd, *J* = 10.9, 0.8 Hz, 1H). ^{13}C-NMR (125 MHz, $CDCl_3$): δ (ppm) 136.6, 135.9, 131.8, 127.9, 121.7, 114.8. LRMS (ESI) $[M+H]^+$: 183.00. Spectroscopic data matched the literature.[8]

3-Bromostyrene (10)

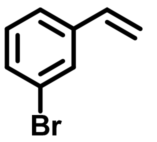

3-Bromobenzaldehyde (0.7 mL, 6.0 mmol, 1.0 eq), $MePPh_3Br$ (4.3 g, 12.0 mmol, 2.0 eq) and 1.35 M *n*-BuLi solution in hexane (6.7 mL, 9.0 mmol, 1.5 eq) were used in 30 mL of anhydrous THF. The residue was purified by column chromatography (Silica gel, 100% pet. ether) to provide **10** as a pale-yellow oil (918 mg, 84%). ^{1}H-NMR (500 MHz, $CDCl_3$): δ (ppm) 7.56 (s, 3H), 7.39-7.37 (d, *J* = 7.9 Hz, 1H), 7.33-7.31 (d, *J* = 7.7 Hz, 1H), 7.21-7.18 (t, *J* = 7.8 Hz, 1H), 6.67-6.62 (dd, *J* = 17.6, 10.8 Hz, 1H), 5.77-5.74 (d, *J* = 17.6, 0.7 Hz, 1H), 5.31- 5.29 (dd, *J* = 10.8, 0.8 Hz, 1H). ^{13}C-NMR (125 MHz, $CDCl_3$): δ (ppm) 139.8, 135.7, 130.8, 130.2, 129.3, 125.0, 122.9, 115.5. LRMS (ESI) $[M+H]^+$: 183.17. Spectroscopic data matched the literature.[9]

2-Bromostyrene (11)

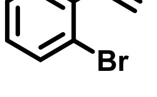

2-Bromobenzaldehyde (1.0 mL, 8.54 mmol, 1.0 eq), $MePPh_3Br$ (6.3 g, 17.5 mmol, 2.0 eq) and 1.35 M *n*-BuLi solution in hexane (9.5 mL, 12.8 mmol, 1.5 eq) were

used in 30 mL of anhydrous THF. The residue was purified by column chromatography (Silica gel, 100% pet. ether) to provide **11** as a colorless oil (910 mg, 57%). ^{1}H-NMR (500 MHz, $CDCl_3$): δ (ppm) 7.56-7.54 (dt, *J* = 8.4, 1.2 Hz, 2H), 7.30-7.27 (m, 1H), 7.14-7.03 (m, 2H), 5.72-5.69 (dd, *J* = 17.5, 1.1 Hz, 1H), 5.38-5.36 (dd, *J* = 11.0, 1.1 Hz, 1H). ^{13}C-NMR (125 MHz, $CDCl_3$): δ (ppm) 137.6, 136.0, 133.0, 129.2, 127.6, 126.9, 123.7, 116.8. LRMS (ESI) $[M+H]^+$: 183.17. Spectroscopic data matched the literature.[10]

2-Vinylnaphthalene (12)

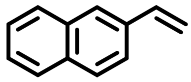

Recrystallized 2-naphthaldehyde (615 mg, 3.94 mmol, 1.0 eq), $MePPh_3Br$ (2.95 g, 8.27 mmol, 2.1 eq) and 1.38 M *n*-BuLi solution in hexane (3.70 mL, 5.12 mmol, 1.3 eq) were used in 30 mL of anhydrous THF. The residue was purified by column chromatography (Silica gel, 100% pet. ether) to provide **12** as a colorless solid (460 mg, 76%). m.p. 65˚C-66˚C (lit. 64˚C-66˚C).[11] ^{1}H-NMR (500 MHz, $CDCl_3$): δ (ppm) 7.83-7.79 (m, 3H), 7.76 (s, 1H), 7.65-7.63 (dd, *J* = 8.5, 1.8 Hz, 1H), 7.48-7.45 (m, 2H), 6.92-6.86 (dd, *J* = 17.6, 10.9 Hz, 1H), 5.90-5.86 (d, *J* = 17.6 Hz, 1H), 5.33 (d, *J* = 10.8 Hz, 1H). ^{13}C-NMR (125 MHz, $CDCl_3$): δ (ppm) 137.0, 135.2, 133.7, 133.3, 128.3, 128.2, 127.8, 126.5, 126.4, 126.1, 123.3, 114.3. LRMS (ESI) $[M+H]^+$: 155.17. Spectroscopic data matched the literature.[12]

3. Electrochemical Procedures

Cyclic Voltammetry and Bulk Electrolysis Experiments

Cyclic voltammograms, and chronocoulometric experiments were recorded with a Bioanalytical Systems Inc. BAS100B/W potentiostat. The configuration for cyclic voltammetry experiments consisted of a Pt (1.6 mm diam.) or GC (3 mm diam.) working electrode, a non-aqueous $Ag^{+/0}$ reference electrode (0.1 M $(Et_4N)(ClO_4)$ in anhydrous CH_3CN), and a Pt wire counter electrode. All experiments were performed at room temperature, and the potentials are referenced *versus* the ferrocenium/ferrocene ($Fc^{+/0}$) couple.

The bulk electrolysis set-up consisted of an 'H-Cell' (Fig. S1) with the cathode divided from the anode by a sintered glass frit. In the working electrode compartment a Pt basket (2.6 × 7.2 × 0.1 cm) or a reticulated vitreous carbon working electrode (3.2 × 7.6 × 0.3 cm), and a non-aqueous $Ag^{+/0}$ reference electrode were installed, and a smaller Pt basket (2.0 × 3.6 × 0.1 cm) or Pt mesh

electrode was added to the counter electrode compartment. The Pt electrodes and glassware used for bulk electrolysis were cleaned before each use with piranha solution (4:1 mixture of H_2SO_4:H_2O_2, *caution!*) and when required, were further cleaned by constant current electrolysis in a 0.5 M H_2SO_4 aqueous solution.

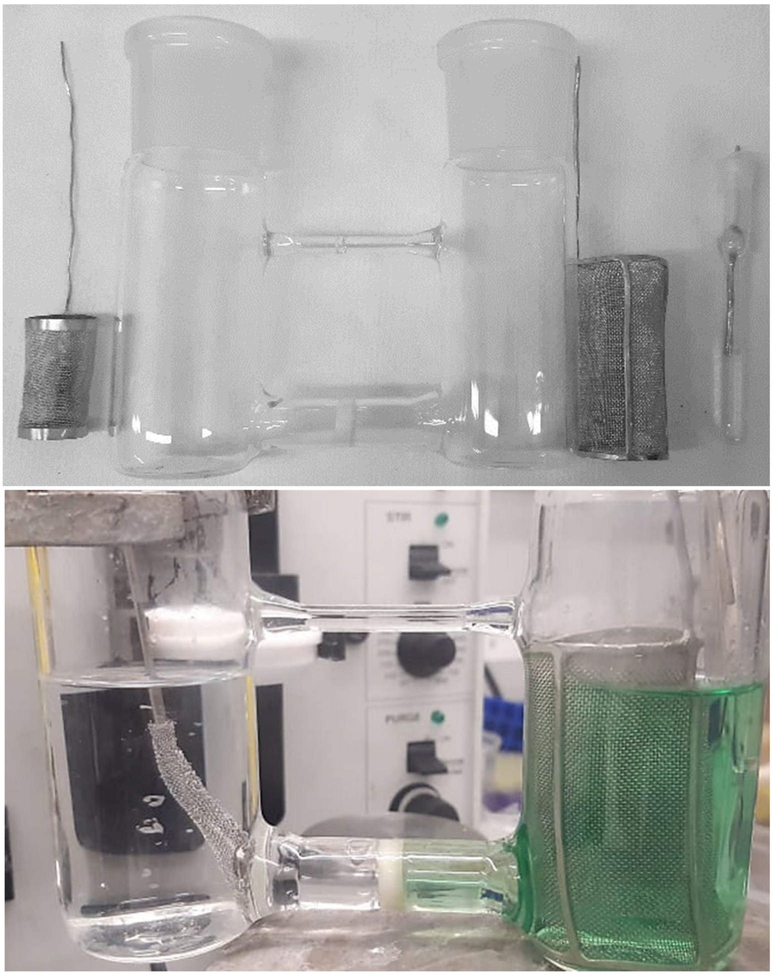

Figure S1. H-cell electrolysis before set-up (top) and during electrolysis (bottom). The green colour of $[Cu(Me_6tren)(CH_2CN)]^+$ is apparent in the working electrode chamber (right).

Work-up Procedure and Purification

The solution in the working electrode compartment was transferred to a beaker and diluted with 50 mL of distilled water and extracted with DCM (3 × 50 mL). The combined organic layers were thoroughly washed with distilled water (3 × 100 mL), and 100 mL of brine. The DCM mixture containing the product was dried over anhydrous $MgSO_4$, filtered, and the solvent was removed

under reduced pressure. The crude product was purified by flash column chromatography on silica gel (230-400 mesh size), with the required solvent mixtures specified in section 6 for each product. Isolated yields are reported.

Catalyst Recovery

The precatalyst $[Cu(Me_6tren)(H_2O)](ClO_4)_2$ was recovered by cation-exchange chromatography over a Sephadex C-25 resin (Na^+ form, 10 × 2 cm). The aqueous extracts from several electrolysis experiments were combined, loaded onto the column, and eluted with 0.3 M aqueous $NaClO_4$ to give a single blue band. Concentration of this blue solution afforded crystals of $[Cu(Me_6tren)](ClO_4)_2$. Further crops were obtained from the mother liquor by taking up the concentrated filtrate in ethanol, layering with petroleum spirits, and cooling at 3 °C to facilitate precipitation.

4. Stoichiometric ATRA of $[Cu(Me_6tren)(CH_2CN)]^+$ with styrene

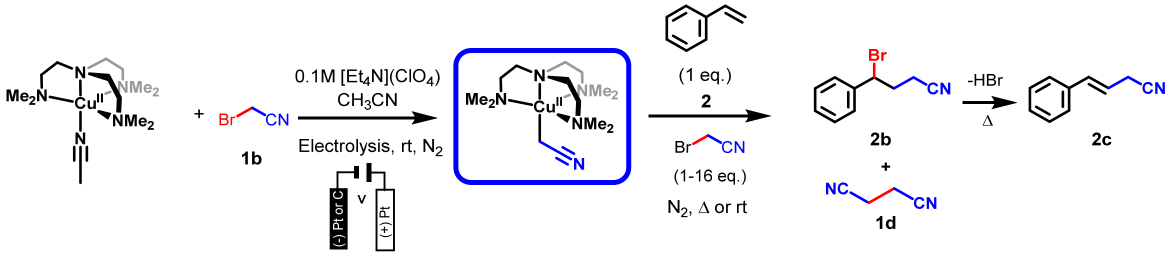

Scheme S1. Conditions for stoichiometric ATRA with $[Cu(Me_6tren)(CH_2CN)]^+$.

A solution of $[Cu(Me_6tren)(H_2O)](ClO_4)_2$ (1 mmol, 49 mg), in 50 mL 0.1 M $(Et_4N)(ClO_4)$ in CH_3CN and initiator (**1a** or **1b**, 1.1 mmol) was added to the working compartment of the H-cell, and 50 mL of 0.1 M $(Et_4N)(ClO_4)$ was added to the other counter electrode compartment. Both compartments were purged with nitrogen for 15 min. The electrolysis potential (E_{app} = –860 mV vs $Fc^{+/0}$) determined from cyclic voltammetry (see Fig. S2) was applied and the nitrogen purge was continued. The formation of $[Cu(Me_6tren)(CH_2CN)]^+$ was monitored by UV-Vis spectroscopy[13] and once fully formed (30 min) the potentiostat was switched off and 10 mL aliquots of the working electrode solution were dispensed into five different Schlenk tubes under an atmosphere of nitrogen, which were then purged with three freeze-pump-thaw cycles each. Different equivalents of de-gassed initiator and styrene were then added to each Schlenk tube (see

Table 1 in the main text) and were reacted at different temperatures for 24 h under nitrogen. The formation percentage of monomer and products was determined by ^{1}H NMR.

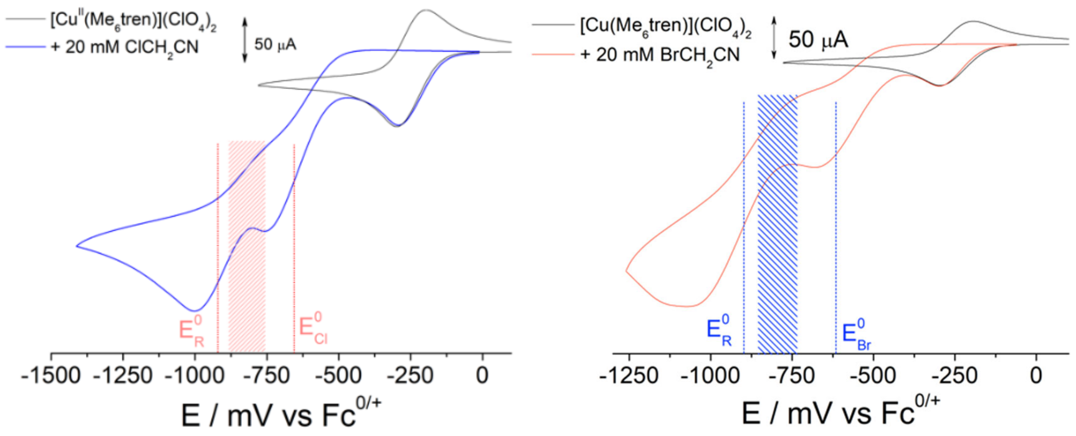

Figure S2. Cyclic voltammetry of $[Cu(Me_6tren)(NCCH_3)]^{2+}$ in the absence and presence of XCH_2CN (X = Cl (**1a**) or Br (**1b**)). The electrolysis potential (E_{app}) lies within the shaded band shown on each voltammogram ($E_R < E_{app} < E_X$, X = Cl, Br).

5. Optimized protocol for eATRA

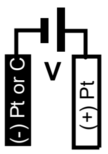

Scheme S2. eATRA conditions.

The ratios of reagents and catalyst were as follows: alkene (**2-15**, 2 mmol), initiator (**1a** or **1b**, 3 mmol, 1.5 eq.), and $[Cu(Me_6tren)](ClO_4)_2$ (0.2 mmol, 0.1 eq.) dissolved in 50 mL of a 0.1 M $(Et_4N)(ClO_4)$ solution in CH_3CN and placed into the working electrode compartment while 50 mL of a 0.1 M $(Et_4N)(ClO_4)$ solution in CH_3CN was added simultaneously to the counter electrode compartment. During electrolysis at potential E_{appl} = –860 mV vs $Fc^{+/0}$ (from cyclic voltammetry, see Fig. S2) a constant stream of nitrogen was maintained to both cell compartments. The reaction

was monitored periodically by thin layer chromatography (silica-60 F245 plates): the product spot was visualized with ultraviolet light (254 nm) and developed with $KMnO_4$, vanillin and phosphomolybdic acid. When the final current was less than 5% of the initial current, the reactions were stopped, which for $ClCH_2CN$ was approximately 12 h, and for $BrCH_2CN$ 8 h; the individual conditions are specified in section S7.

6. Reaction conditions for the electro-synthesis of dimeric products

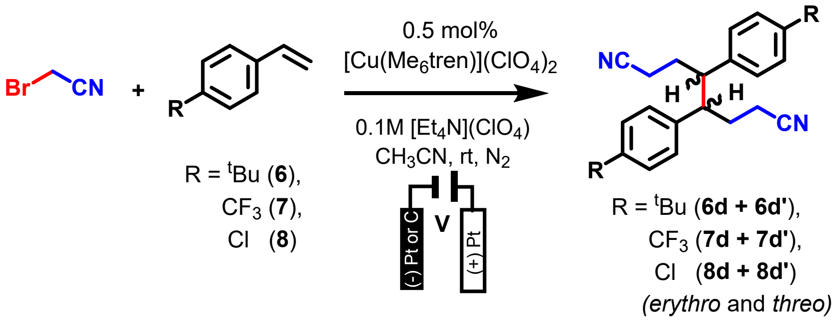

The same procedure as described above (section 5) was carried out except the ratios of reagents and catalyst were as follows: alkene (**6-8**, 2 mmol), $BrCH_2CN$ (**1b**, 2.4 mmol, 1.2 eq.), and $[Cu(Me_6tren)](ClO_4)_2$ (0.01 mmol, 0.005 eq.) dissolved in 50 mL of a 0.1 M $(Et_4N)(ClO_4)$ solution in CH_3CN all in the working electrode compartment. During electrolysis at potential E_{app} = –860 mV vs $Fc^{+/0}$ (from cyclic voltammetry, see Fig. S2) a constant stream of nitrogen was maintained. When the current was less than 5% of the initial current, the reactions were stopped (approx. 12h). Individual conditions are described in section 7.

7. Characterization Data of Products

4-Chloro-4-phenylbutanenitrile (2a)

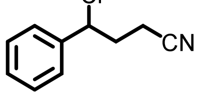

Styrene (**2**, 250 mg, 2.40 mmol, 1.0 eq), $ClCH_2CN$ (**1a**, 272 mg, 3.60 mmol, 1.5 eq) and **$[Cu(Me_6tren)](ClO_4)_2$** (118 mg, 0.24 mmol, 0.1 eq) in 50 mL of anhydrous CH_3CN were used. The reaction was stopped after 10 h of electrolysis at -900 mV vs $Fc^{0/+}$. The residue was purified by column chromatography (Silica gel, 5%-20% EtOAc in pet. ether and then 50%-75% DCM in pet. ether) to provide **2a** as a pale-yellow oil (354 mg, 82%). ^{1}H-NMR (500 MHz, $CDCl_3$): δ (ppm) 7.40-7.33 (m, 5H), 5.00-4.97

(dd, J = 8.9, 5.5 Hz, 1H), 3.05-2.63 (m, 1H), 2.45-2.31 (m, 3H). ^{13}C-NMR (125 MHz, $CDCl_3$): δ (ppm) 139.9, 129.1, 129.1, 126.9, 118.6, 61.3, 35.6, 15.4. LRMS (ESI) $[M+H]^+$: 180.17. Spectroscopic data matched the literature.[14]

4-Chloro-4-(*p*-tolyl)butanenitrile (3a)

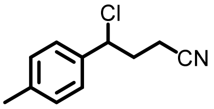

4-Methylstyrene (**3**, 205 mg, 1.73 mmol, 1.0 eq), $ClCH_2CN$ (**1a**, 195 mg, 2.60 mmol, 1.5 eq.), and **[Cu(Me$_6$tren)](ClO$_4$)$_2$** (95 mg, 0.20 mmol, 0.1 eq) in 50 mL of anhydrous CH_3CN were used. The reaction was stopped after 12 h of electrolysis at -900 mV vs $Fc^{0/+}$. The residue was purified by column chromatography (Silica gel, 10%-25% EtOAc in pet. ether) to provide **3a** as a pale-yellow oil (322 mg, 82%). ^{1}H-NMR (500 MHz, $CDCl_3$): δ (ppm) 7.28-7.29 (m, 2H), 7.20-2.18 (m, 2H), 4.97-4.94 (m, 1H), 2.61-2.54 (m, 1H), 2.51-2.40 (m, 2H), 2.39-2.30 (m, 3H), 2.36 (s, 3H). ^{13}C-NMR (125 MHz, $CDCl_3$): δ (ppm) 139.1, 136.4, 129.8, 126.9, 121.1, 118.6, 61.3, 35.6, 21.3, 15.4. LRMS (ESI) $[M+H]^+$: 194.00. Spectroscopic data matched the literature.[15]

4-Chloro-4-(4-methoxyphenyl)butanenitrile (4a)

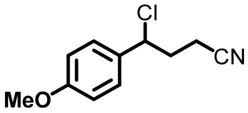

4-Vinylanisole (**4**, 204 mg, 1.52 mmol, 1.0 eq), $ClCH_2CN$ (**1a**, 173 mg, 2.30 mmol, 1.5 eq) and **[Cu(Me$_6$tren)](ClO$_4$)$_2$** (75 mg, 0.15 mmol, 0.1 eq) in 50 mL of anhydrous CH_3CN were used. The reaction was stopped after 12 h of electrolysis at -900 mV vs $Fc^{0/+}$. The residue was purified by column chromatography (Silica gel, 15%-25% EtOAc in pet. ether) to provide **4a** as a pale-yellow oil (252 mg, 79%). ^{1}H-NMR (500 MHz, $CDCl_3$): δ (ppm) 7.29-7.28 (m, 2H), 6.91-6.90 (m, 2H), 4.80-4.77 (m, 1H), 2.54-2.50 (m, 1H), 2.43-2.35 (m, 1H), 2.13-1.95 m, 2H). ^{13}C-NMR (125 MHz, $CDCl_3$): δ (ppm) 159.7, 135.2, 127.1, 119.7, 114.3, 72.2, 55.47, 34.3, 14.0. HRMS m/z calculated for: $[C_{11}H_{13}ClNO]^+$ $[M+H]^+$: 210.0681; found 210.0684.

4-Chloro-4-(4-isopropylphenyl)butanenitrile (5a)

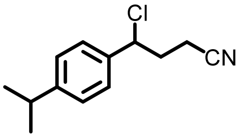

4-Isopropyl styrene (**5**, 1.37 mmol, 1.0 eq), $ClCH_2CN$ (**1a**, 155 mg, 2.06 mmol, 1.5 eq) and **[Cu(Me$_6$tren)](ClO$_4$)$_2$** (67 mg, 0.14 mmol, 0.1 eq) in 50 mL of anhydrous CH_3CN were used. The residue was purified by column chromatography (Silica gel, 5%-20% EtOAc in pet. Ether then 50-75% DMC/pet. Ether) to

provide a mixture of **5a** and **5a'** as a pale-yellow oil. The reaction was stopped after 12 h of electrolysis at -900 mV vs $Fc^{0/+}$. The residue was purified by column chromatography (Silica gel, 5%-20% EtOAc in pet. ether, then 50%-75% DCM in pet. ether) to provide the **5a/5a'** mixture as a colorless oil. ^{1}H-NMR (500 MHz, $CDCl_3$): 7.34-7.28 (m, 2H), 7.27-7.24 (m, 2H), 5.00-4.97 (m, 1H), 2.98-2.88 (m, 1H), 2.62-2.32 (m, 4H), 1.27-1.25 (d, J = 6.9 Hz, 6H). ^{13}C-NMR (125 MHz, $CDCl_3$): δ (ppm) 150.00, 137.21, 127.18, 126.92, 118.66, 61.35, 35.54, 35.15, 15.66, 15.41. HRMS m/z calculated for: $[C_{13}H_{17}ClN]^+$ $[M+H]^+$: 222.1045; found 222.1047.

4-Chloro-4-(4-isopropylphenyl)butanenitrile (5a')

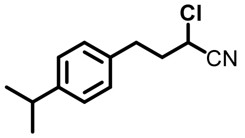

The structure of this isomer was elucidated by 2D-NMR. ^{1}H-NMR (500 MHz, $CDCl_3$): 7.44-7.32 (m, 2H), 7.27-7.24 (m, 2H), 5.43-5.38 (m, 1H), 3.33-3.24 (m, 1H), 2.72-2.32 (m, 4H), 1.32-1.29 (d, J = 6.8 Hz, 6H). ^{13}C-NMR (125 MHz, $CDCl_3$): δ (ppm) 146.09, 136.63, 129.22, 126.62, 126.02, 56.92, 33.99, 28.70, 23.99.

4-(4-(*Tert*-butyl)phenyl)-4-chlorobutanenitrile (6a)

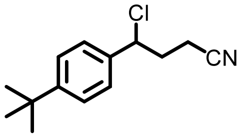

4-*Tert*-butylstyrene (**6**, 238 mg, 1.48 mmol, 1.0 eq), $ClCH_2CN$ (**1a**, 168 mg, 2.22 mmol, 1.5 eq) and **[Cu(Me$_6$tren)](ClO$_4$)$_2$** (73 mg, 0.15 mmol, 0.1 eq) in 50 mL of anhydrous CH_3CN were used. The residue was purified by column chromatography (Silica gel, 10%-20% EtOAc in pet. ether) to provide **6a** as a clear oil (267 mg, 82%). ^{1}H-NMR (500 MHz, $CDCl_3$): δ (ppm) 7.42-7.39 (dt, J = 8.8, 2.2 Hz, 2H), 7.32-7.31 (dt, J = 8.2, 2.2 Hz, 2H), 4.99-4.96 (dd, J = 9.0, 5.4 Hz, 1H), 2.62-2.55 (m, 1H), 2.52-2.31 (m, 3H), 1.32 (s, 9H). ^{13}C-NMR (125 MHz, $CDCl_3$): δ (ppm) 152.3, 126.8, 126.7, 126.1, 118.7, 61.2, 35.5, 34.8, 31.4, 15.4. HRMS (ESI): m/z calculated for: $[C_{14}H_{18}ClNNa]^+$ $[M+Na]^+$: 258.1020; found 258.1021.

4-Chloro-4-(4-(trifluoromethyl)phenyl)butanenitrile (7a)

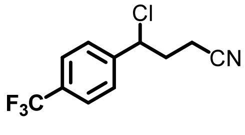

4-Trifluoromethylstyrene (**7**, 220 mg, 1.28 mmol, 1.0 eq), $ClCH_2CN$ (**1a**, 145 mg, 1.92 mmol, 1.5 eq) and **[Cu(Me$_6$tren)](ClO$_4$)$_2$** (63 mg, 0.13 mmol, 0.1 eq) in 50 mL of anhydrous CH_3CN were used. The reaction was stopped after 11 h of electrolysis at -900 mV vs $Fc^{0/+}$. The residue was purified by column chromatography

(Silica gel, 15%-30% EtOAc in pet. ether) to provide **7a** as a pale-yellow oil (253 mg, 80%). ^{1}H-NMR (500 MHz, $CDCl_3$): δ (ppm) 7.71-7.60 (m, 2H), 7.60-7.49 (m, 2H), 5.06-4.97 (m, 1H), 2.70-2.59 (m, 1H), 2.59-2.48 (m, 1H), 2.48-2.28 (m, 2H). ^{13}C-NMR (125 MHz, $CDCl_3$): δ (ppm) 143.7, 131.4, 127.4, 126.2, 124.9, 122.8, 118.3, 60.2, 35.5, 15.5. HRMS m/z calculated for: $[C_{11}H_{10}ClF_3N]^+$ $[M+H]^+$: 248.0449; found 248.0451.

4-Chloro-4-(4-chlorophenyl)butanenitrile (8a)

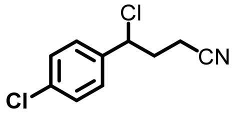

4-Chlorostyrene (**8**, 236 mg, 1.70 mmol, 1.0 eq), $ClCH_2CN$ (**1a**, 193 mg, 2.55 mmol, 1.5 eq) and **[Cu(Me$_6$tren)](ClO$_4$)$_2$** (84 mg, 0.17 mmol, 0.1 eq) in 50 mL of anhydrous CH_3CN were used. The reaction was stopped after 12 h of electrolysis at -900 mV vs $Fc^{0/+}$. The residue was purified by column chromatography (Silica gel, 50%-70% DCM in pet. ether) to provide **8a** as a clear oil (351 mg, 96%). ^{1}H-NMR (500 MHz, $CDCl_3$): δ (ppm) 7.38-7.32 (m, 4H), 4.97-4.94 (dd, *J* = 9.2, 5.3 Hz, 1H), 2.64-2.57 (m, 1H), 2.52-2.46 (m, 1H), 2.43-2.28 (m, 2H). ^{13}C-NMR (125 MHz, $CDCl_3$): δ (ppm) 138.4, 135.0, 129.4, 128.4, 118.4, 60.4, 35.5, 15.4. HRMS (ESI): m/z calculated for: $[C_{10}H_{10}Cl_2N]^+$ $[M+H]^+$: 214.0185; found 214.0183.

4-(4-Bromophenyl)-4-chlorobutanenitrile (9a)

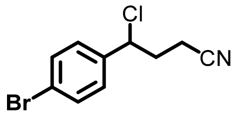

4-Bromostyrene (**9**, 215 mg, 1.18 mmol, 1.0 eq), $ClCH_2CN$ (**1a**, 134 mg, 1.77 mmol, 1.5 eq) and **[Cu(Me$_6$tren)](ClO$_4$)$_2$** (58 mg, 0.12 mmol, 0.1 eq) in 50 mL of anhydrous CH_3CN were used. The reaction was stopped after 12 h of electrolysis at -900 mV vs $Fc^{0/+}$. The residue was purified by column chromatography (Silica gel, 10%-30% EtOAc in pet. ether) to provide **9a** as a yellow oil (267 mg, 88%). ^{1}H-NMR (500 MHz, $CDCl_3$): δ (ppm) 7.54-7.51 (m, 2H), 7.29-7.26 (m, 2H), 4.96-4.93 (dd, *J* = 9.2, 5.5 Hz, 1H), 2.64-2.57 (m, 1H), 2.52-2.46 (m, 1H), 2.42-2.27 (m, 2H). ^{13}C-NMR (125 MHz, $CDCl_3$): δ (ppm) 138.9, 132.3, 128.6, 123.1, 118.4, 60.4, 35.5, 15.4. HRMS (ESI): m/z calculated for: $[C_{10}H_9ClBrNNa]^+$ $[M+Na]^+$: 279.9500; found: 279.9490.

4-(3-Bromophenyl)-4-chlorobutanenitrile (10a)

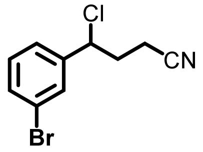

3-bromostyrene (**10**, 240 mg, 1.31 mmol, 1.0 eq), $ClCH_2CN$ (**1a**, 160 mg, 2.00 mmol, 1.5 eq) and **[Cu(Me$_6$tren)](ClO$_4$)$_2$** (62 mg, 0.13 mmol, 0.1 eq) in 50 mL of anhydrous CH_3CN were used. The reaction was stopped after 12 h of electrolysis at -900 mV vs $Fc^{0/+}$. The residue was purified by column chromatography (Silica gel,) to provide **10a** as a clear oil (280 mg , 83%). ^{1}H-NMR (500 MHz, $CDCl_3$): δ (ppm) 7.56-7.55 (m, 1H), 7.51-7.47 (m, 1H), 7.34-7.31 (m, 1H), 7.28-7.24 (m, 1H), 4.94-4.91 (m, 1H), 2.65-2.58 (m, 1H), 2.54-2.47 (m, 1H), 2.42-2.92 (m, 2H). ^{13}C-NMR (125 MHz, $CDCl_3$): δ (ppm) 142.1, 132.3, 130.7, 130.1, 125.7, 123.1, 118.4, 60.2, 35.5, 15.3. HRMS (ESI): m/z calculated for: $[C_{10}H_{10}ClBrN]^+$ 257.9680, found: 257.9681.

4-(2-Bromophenyl)-4-chlorobutanenitrile (11a)

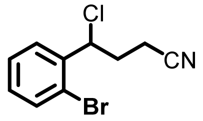

2-bromostyrene (**11**, 240 mg, 1.31 mmol, 1.0 eq), $ClCH_2CN$ (**1a**, 160 mg, 2.00 mmol, 1.5 eq) and **[Cu(Me$_6$tren)](ClO$_4$)$_2$** (62 mg, 0.13 mmol, 0.1 eq) in 50 mL of anhydrous CH_3CN were used. The reaction was stopped after 12 h of electrolysis at -900 mV vs $Fc^{0/+}$. The residue was purified by column chromatography (Silica gel, 5-10% Et_2O in pet. ether) to provide **11a** as a clear oil (270 mg, 80%).^{1}H-NMR (500 MHz, $CDCl_3$): δ (ppm) 7.61-7.56 (m, 2H), 7.41 – 7.36 (m, 1H), 7.22-7.17 (m, 1H), 5.47 (dd, J = 7.5, 3.6 Hz, 1H), 2.63 – 2.59 (m, 2H), 2.43 – 2.28 (m, 2H). ^{13}C-NMR (125 MHz, $CDCl_3$): δ (ppm) 138.9, 133.3, 130.3, 128.6, 128.4, 122.8, 118.5, 60.0, 34.9, 15.2. HRMS (ESI): m/z calculated for: $[C_{10}H_{10}ClBrN]^+$ 257.9680, found: 257.9679.

4-Chloro-4-(naphthalen-2-yl)butanenitrile (12a)

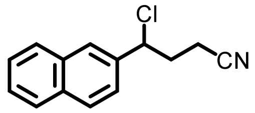

2-Vinylnaphthalene (**12**, 42 mg, 0.27 mmol, 1.0 eq), $ClCH_2CN$ (**1a**, 17.2 mg, 0.55 mmol, 2.0 eq) and **[Cu(Me$_6$tren)](ClO$_4$)$_2$** (13 mg, 0.27 mmol, 0.1 eq) in 50 mL of anhydrous CH_3CN were used. The reaction was stopped after 12 h of electrolysis at -920 mV vs $Fc^{0/+}$. The residue was purified by column chromatography (Silica gel, 15%-25% EtOAc in pet. ether) to provide **12a** as colorless crystals (52 mg, 84%). m.p. 122.6˚C-123.1˚C. ^{1}H-NMR (500 MHz, $CDCl_3$): δ (ppm) 7.90-7.83 (m, 4H), 7.55-7.50 (m, 3H), 5.18-5.15 (dd, J = 8.6, 5.5 Hz, 1H), 2.67-2.42 (m, 4H). ^{13}C-NMR (125 MHz, $CDCl_3$): δ (ppm) 137.0, 133.5, 133.1, 129.4, 128.3, 127.9, 127.0, 126.9, 126.3, 124.1, 118.6, 61.6, 35.5, 15.4.

HRMS (ESI): m/z calculated for: $[C_{14}H_{13}ClN]^+$ $[M+H]^+$: 230.0732; found 230.0729, $[C_{14}H_{12}ClNNa]^+$ $[M+Na]^+$: 252.0551; found 252.0549.

4-Chlorodecanenitrile (13a)

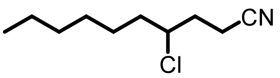

1-Octene (**13**, 200 mg, 1.78 mmol, 1.0 eq), $ClCH_2CN$ (**1a**, 202 mg, 2.67 mmol, 1.5 eq) and **[Cu(Me$_6$tren)](ClO_4)$_2$** (87 mg, 0.18 mmol, 0.1 eq) in 50 mL of anhydrous CH_3CN were used. The reaction was stopped after 14 h of electrolysis at -920 mV vs $Fc^{0/+}$. The residue was purified by column chromatography (Silica gel, 30%-60% DCM in pet. ether and then 5% EtOAc in pet. ether) to provide **13a** as a pale-yellow oil (135 mg, 41%). ^{1}H-NMR (500 MHz, $CDCl_3$): δ (ppm) 4.00-3.95 (m, 1H), 2.61-2.58 (m, 2H), 2.16-2.10 (m, 1H), 2.00-1.92 (m, 1H), 1.78-1.73 (m, 2H), 1.57-1.49 (m, 1H), 1.47-1.38 (m, 1H), 1.27-1.25 (m, 6H), 0.91-0.88 (m, 3H).^{13}C-NMR (125 MHz, $CDCl_3$): δ (ppm) 119.1, 61.6, 38.4, 34.2, 31.8, 28.8, 26.5, 22.7, 15.0, 14.2. HRMS (ESI): m/z calculated for: $[C_{10}H_{19}ClN]^+$ $[M+H]^+$: 188.1201; found 188.1204, $[C_{10}H_{18}ClNNa]^+$ $[M+Na]^+$: 210.1020; found 210.1024.

Methyl 2-chloro-4-cyano-2-methylbutanoate (14a)

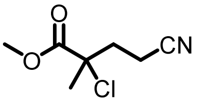

Methyl methacrylate (**14**, 213.3 mg, 2.13 mmol, 1.0 eq), $ClCH_2CN$ (**1a**, 241 mg, 3.20 mmol, 1.5 eq) and **[Cu(Me$_6$tren)](ClO_4)$_2$** (100 mg, 0.20 mmol, 0.09 eq) in 50 mL of anhydrous CH_3CN were used. The reaction was stopped after 14 h of electrolysis at -920 mV vs $Fc^{0/+}$. The residue was purified by column chromatography (Silica gel, 40%-60% EtOAc in pet. ether and then 100% DCM) to provide **14a** as a pale-yellow oil (250 mg, 67%). ^{1}H-NMR (500 MHz, $CDCl_3$): δ (ppm) 3.82 (s, 3H), 2.61-2.57, 2.53-2.47 (m, 1H), 2.29-2.23 (m, 1H), 1.80 (s, 3H). ^{13}C-NMR (125 MHz, $CDCl_3$): 170.7, 118.8, 67.0, 65.7, 37.5, 28.4, 13.6. HRMS (ESI): m/z calculated for: $[C_7H_{11}ClNO_2]^+$ $[M+H]^+$: 176.0473; found 176.0466, $[C_7H_{10}ClNO_2Na]^+$ $[M+Na]^+$: 198.0292; found 198.0285.

2-Methoxyethyl 2-chloro-4-cyanobutanoate (15a)

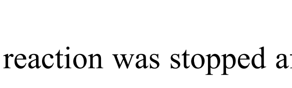

2-(Methoxyethyl) acrylate (**15**, 220 mg, 1.69 mmol, 1.0 eq), $ClCH_2CN$ (**1a**, 190 mg, 2.52 mmol, 1.5 eq) and **[Cu(Me$_6$tren)](ClO_4)$_2$** (78 mg, 0.16 mmol, 0.1 eq) in 50 mL of anhydrous CH_3CN were used. The reaction was stopped after 12 h of electrolysis at -920 mV vs $Fc^{0/+}$. The residue was purified by

column chromatography (Silica gel, 5%-25% EtOAc in pet. ether) to provide **15a** as a pale-yellow oil (245 mg, 71%).^{1}H-NMR (500 MHz, $CDCl_3$): δ (ppm) 4.47-4.44 (m, 1H), 4.39-4.29 (m, 2H), 3.60 (t, 2H), 3.37 (s, 3H), 2.60 (t, 2H), 2.42-2.35 (m, 1H), 2.31-2.25 (m, 1H). ^{13}C-NMR (125 MHz, $CDCl_3$): 168.3, 118.2, 70.0, 65.4, 59.1, 55.1, 30.5, 14.1. HRMS (ESI): m/z calculated for: $[C_8H_{13}ClNO_3]^+$ $[M+H]^+$: 206.0579; found 206.0583.

2-(2-Chlorocyclooctyl)acetonitrile (16a)

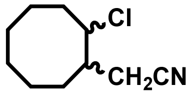

Cis-cyclooctene (**16**, 220 mg, 2.00 mmol, 1.0 eq), $ClCH_2CN$ (**1a**, 240 mg, 3.00mmol, 1.5 eq) and **[Cu(Me$_6$tren)](ClO$_4$)$_2$** (80 mg, 0.16 mmol, 0.08 eq) were used. The residue was purified by column chromatography (Silica gel, 20% Et_2O in pet. ether) to provide a mixture of **16a** as a colorless oil (172 mg, 46%). ^{1}H-NMR (500 MHz, $CDCl_3$, mixture of isomers 1:8): δ (ppm) 4.32-4.28 (m, 1H), 4.01-3.97 (m, 8H), 2.72-2.68 (m, 8H), 2.60-2.55 (m, 10H), 2.45-2.37 (m, 2H), 2.26-2.13 (m, 18H), 2.09-2.02 (m, 10H), 1.90-1.40 (m, 78H). ^{13}C-NMR (125 MHz, $CDCl_3$, mixture of *syn* and *anti* isomers): δ (ppm) 119.0, 118.6, 66.5, 65.5, 42.4, 38.2, 34.7, 32.7, 30.1, 28.0, 27.9, 27.8, 26.4, 26.2, 25.2, 25.1, 24.5, 23.7, 23.4, 23.2. HRMS (ESI): m/z calculated for: $[C_{10}H_{17}ClN]^+$ $[M+H]^+$: 186.1045; found 186.1044.

4-Bromo-4-phenylbutanenitrile (2b)

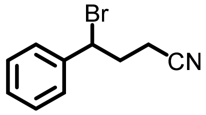

Styrene (**2**, 208 mg, 2.0 mmol, 1.0 eq), $BrCH_2CN$ (**1b**, 360 mg, 3.0 mmol, 1.5 eq) and **[Cu(Me$_6$tren)](ClO$_4$)$_2$** (75 mg, 0.15 mmol, 0.075 eq) in 50 mL of anhydrous CH_3CN were used. The reaction was stopped after 7 h of electrolysis at -900 mV vs $Fc^{0/+}$. The residue was purified by column chromatography (Silica gel, 20%-40% Et_2O in pet.) to provide **2b** as a pale-yellow oil (322 mg, 72%). ^{1}H-NMR (500 MHz, $CDCl_3$): δ (ppm) 7.42-7.32 (m, 5H), 5.07-5.02 (m, 1H), 2.62-2.39 (m, 4H). ^{13}C-NMR (125 MHz, $CDCl_3$): δ (ppm) 140.3, 129.2, 127.3, 118.4, 52.3, 35.5, 16.6. LRMS (ESI) $[M+H]^+$: 224.08. Spectroscopic data matched the literature.[16]

4-Bromo-4-(*p*-tolyl)butanenitrile (3b)

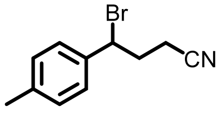

4-Methylstyrene (**3**, 260 mg, 2.20 mmol, 1.0 eq), $BrCH_2CN$ (**1b**, 394 mg, 3.30 mmol, 1.5 eq) and **[Cu(Me$_6$tren)](ClO$_4$)$_2$** (98 mg, 0.20 mmol, 0.1 eq) in 50 mL of anhydrous CH_3CN were used. The reaction was stopped after 7 h of

electrolysis at -900 mV vs $Fc^{0/+}$. The residue was purified by column chromatography (Silica gel, 10%-25% EtOAc in pet. ether) to provide **3b** as a colorless oil (334 mg, 83%). ^{1}H-NMR (500 MHz, $CDCl_3$): δ (ppm) 7.31-7-26 (m, 2H), 5.05-5.01 (m, 1H), 2.61-2.39 (m, 4H), 2.36 (s, 3H). ^{13}C-NMR (125 MHz, $CDCl_3$): δ (ppm) 139.2, 137.3, 129.8, 127.2, 118.5, 52.5, 35.5, 21.3, 16.6. LRMS (ESI) $[M+H]^+$: 238.08. Spectroscopic data matched the literature.[16]

4-Bromo-4-(4-*Tert*-butylphenyl)butanenitrile (6b)

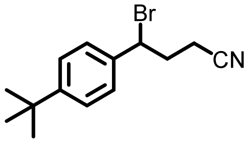

4-*Tert-butyl* styrene (**6**, 238 mg, 1.48 mmol, 1.0 eq), $BrCH_2CN$ (**1b**, 168 mg, 2.22 mmol, 1.5 eq) and **$[Cu(Me_6tren)](ClO_4)_2$** (73 mg, 0.15 mmol, 0.1 eq) in 50 mL of anhydrous CH_3CN were used. The reaction was stopped after 8 h of electrolysis at -900 mV vs $Fc^{0/+}$. The residue was purified by column chromatography (Silica gel, 10%-20% EtOAc in pet. ether) to provide **6b** as a clear oil (267 mg, 82%). ^{1}H-NMR (500 MHz, $CDCl_3$): 7.42-7.37 (m, 2H), 7.34 – 7.26 (m, 2H), 4.86-4.79 (m, 1H), 2.59-2.49 (m, 1H), 2.46-2.39 (m, 1H), 2.14-1.99 (m, 2H). ^{13}C-NMR (125 MHz, $CDCl_3$): δ (ppm) 151.5, 140.1, 125.9, 125.6, 119.8, 72.4, 34.8, 34.3, 31.5, 14.0. HRMS (ESI): m/z calculated for: $[C_{14}H_{18}ClNH]^+$ $[M+H]^+$: 280.0696; found 280.0699.

4-Bromo-4-(4-chlorophenyl)butanenitrile (8b)

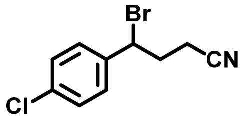

4-Chlorostyrene (**8**, 193 mg, 1.06 mmol, 1.0 eq), $BrCH_2CN$ (**1b**, 190 mg, 1.58 mmol, 1.5 eq) and **$[Cu(Me_6tren)](ClO_4)_2$** (54 mg, 0.11 mmol, 0.1 eq) in 50 mL of anhydrous CH_3CN were used. The reaction was stopped after 8 h of electrolysis at -900 mV vs $Fc^{0/+}$. The residue was purified by column chromatography (Silica gel, 15%-25% EtOAc in pet. ether) to provide **8b** as a colorless solid (189 mg, 59%). m.p. 62.5˚C-63.2˚C. ^{1}H-NMR (500 MHz, $CDCl_3$): δ (ppm) 7.55-7.52 (m, 2H), 7.32-7.29 (m, 2H), 5.03-5.0 (m, 1H), 2.65-2.49 (m, 3H), 2.44-2.36 (m, 1H). ^{13}C-NMR (125 MHz, $CDCl_3$): δ (ppm) 139.4, 132.4, 129.0, 123.2, 118.2, 51.1, 35.4, 16.6. HRMS (ESI): m/z calculated for: $[C_{10}H_{10}BrClN]^+$ $[M+H]^+$: 257.9680; found 257.9673.

4-Bromo-4-(4-bromophenyl)butanenitrile (9b)

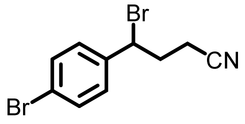

4-Bromostyrene (**9**, 193 mg, 1.06 mmol, 1.0 eq), $BrCH_2CN$ (**1b**, 190 mg, 1.58 mmol, 1.5 eq) and **[Cu(Me$_6$tren)](ClO_4)$_2$** (54 mg, 0.11 mmol, 0.1 eq) in 50 mL of anhydrous CH_3CN were used. The reaction was stopped after 8 h of electrolysis at -900 mV vs $Fc^{0/+}$. The residue was purified by column chromatography (Silica gel, 15%-25% EtOAc in pet. ether) to provide **9b** as a colorless solid (189 mg, 59%). Crystals suitable for XRD were obtained by slow diffusion of hexane into EtOAc. m.p. 62.5˚C-63.2˚C. ^{1}H-NMR (500 MHz, $CDCl_3$): δ (ppm) 7.55-7.52 (m, 2H), 7.32-7.29 (m, 2H), 5.03-5.0 (m, 1H), 2.65-2.49 (m, 3H), 2.44-2.36 (m, 1H). ^{13}C-NMR (125 MHz, $CDCl_3$): δ (ppm) 139.4, 132.4, 129.0, 123.2, 118.2, 51.1, 35.4, 16.6. HRMS (ESI): m/z calculated for: $[C_{10}H_{10}Br_2N]^+$ $[M+H]^+$: 303.9155; found 303.9159.

4-Bromo-4-(naphthalen-2yl)butanenitrile (12b)

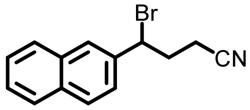

2-Vinylnaphthalene (**12**, 138 mg, 0.89 mmol, 1.0 eq), $BrCH_2CN$ (**1b**, 161 mg, 1.34 mmol, 1.5 eq), and **[Cu(Me$_6$tren)](ClO_4)$_2$** (44 mg, 0.09 mmol, 0.1 eq) in 50 mL of anhydrous CH_3CN were used. The reaction was stopped after 9 h of electrolysis at -900 mV vs $Fc^{0/+}$. The residue was purified by column chromatography (Silica gel, 15%-25% EtOAc in pet. ether) to provide **12b** as colorless crystals (200 mg, 82%). m.p. 115.6°C-116.0°C. ^{1}H-NMR (500 MHz, $CDCl_3$): δ (ppm) 7.89-7.83 (m, 4H), 7.55-7.71 (m, 3H), 5.24-5.21 (dd, J = 9.0, 5.6 Hz, 1H), 2.73-2.49 (m, 4H). ^{13}C-NMR (125 MHz, $CDCl_3$): δ (ppm) 137.4, 133.6, 133.2, 129.4, 128.3, 127.9, 127.1, 127.0, 126.5. 124.6, 118.4, 52.6, 25.4, 16.6. HRMS (ESI): m/z calculated for: $[C_{14}H_{12}BrNNa]^+$ $[M+Na]^+$: 296.0046; found 296.0045.

Methyl 2-bromo-4-cyano-2-methylbutanoate (14b)

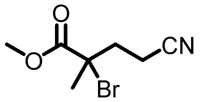

Methyl methacrylate (**14**, 250 mg, 2.50 mmol, 1.0 eq), $BrCH_2CN$ (**1b**, 450 mg, 3.75 mmol, 1.5 eq) and **[Cu(Me$_6$tren)](ClO_4)$_2$** (123 mg, 0.25 mmol, 0.1 eq) in 50 mL of anhydrous CH_3CN were used. The reaction was stopped after 9 h of electrolysis at -920 mV vs $Fc^{0/+}$. The residue was purified by column chromatography (Silica gel, 30%-50% EtOAc in pet. ether) to provide **14b** as a colorless oil (186 mg, 34%).^{1}H-NMR (500 MHz, $CDCl_3$): δ (ppm) 3.82 (s, 3H), 2.69-2.49 (m, 3H), 2.38-2.32 (m, 1H), 2.32-1.95 (s, 3H). ^{13}C-NMR (125 MHz,

$CDCl_3$): δ (ppm) 170.8, 118.8, 58.3, 53.7, 38.0, 28.7, 14.9. HRMS (ESI): m/z calculated for: [$C_7H_{10}BrNO_2Na$] $[M+Na]^+$: 241.9788; found 241.9792.

2-Methoxyethyl 2-bromo-4-cyanobutanoate (15b)

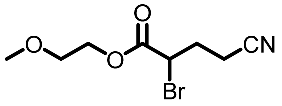

2-Methoxyethyl acrylate (**14**, 218 mg, 1.68 mmol, 1.0 eq), $BrCH_2CN$ (**1b**, 302 mg, 2.52 mmol, 1.5 eq) and **$[Cu(Me_6tren)](ClO_4)_2$** (82 mg, 0.17 mmol, 0.1 eq) in 50 mL of anhydrous CH_3CN were used. The reaction was stopped after 8 h of electrolysis at -920 mV vs $Fc^{0/+}$. The residue was purified by column chromatography (Silica gel, 15%-50% EtOAc in pet. ether) to provide **15b** as a pale-yellow oil (17 mg, 4%). ^{1}H-NMR (500 MHz, $CDCl_3$): δ (ppm) 4.49-4.31 (m, 3H), 3.66-3.59 (m, 2H), 3.39 (s, 3H), 2.66-2.56 (m, 2H), 2.45-2.29 (m, 2H). ^{13}C-NMR (125 MHz, $CDCl_3$): δ (ppm) 68.7, 118.1, 70.1, 65.4, 59.2, 43.1, 30.4, 15.4. HRMS (ESI): m/z calculated for: [$C_8H_{12}BrNO_3Na$] $[M+Na]^+$: 250.0074; found 250.0072.

***Erythro*-4,5-*bis*(4-(*tert*-butyl)phenyl)octanedinitrile (6d)**

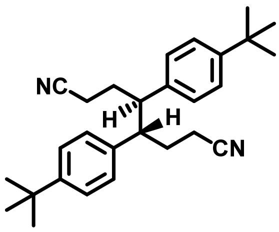

4-*Tert-butyl* styrene (**6**, 240 mg, 1.65 mmol, 1.0 eq), $BrCH_2CN$ (**1b**, 200 mg, 1.98 mmol, 1.2 eq) and **$[Cu(Me_6tren)](ClO_4)_2$** (5 mg, 0.0082 mmol, 0.005 eq) in 50 mL of anhydrous CH_3CN were used. The reaction was stopped after 12 h of electrolysis at -900 mV vs $Fc^{0/+}$. The residue was purified by column chromatography (Silica gel, 5%-50% Et_2O in hexane) to provide a mixture of *erythro* **(6d)** and *threo* **(6d')** isomers as a colorless solid (267 mg, 82%, ratio of 2:1, respectively). The *erythro* isomer was crystallized by vapor diffusion of hexane into an EtOAc solution to afford colorless crystals suitable for XRD. m.p. 224˚C-226˚C ^{1}H-NMR (500 MHz, $CDCl_3$): δ (ppm) 7.41-7.38 (m, 4H), 7.19-7.16 (m, 4H), 2.80 (m, 2H), 2.00-1.96 (m, 1 H), 1.86-1.78 (m, 2H), 1.73-1.65 (m, 2H), 1.63-1.57 (m, 1H), 1.35 (s, 18H). ^{13}C-NMR (125 MHz, $CDCl_3$): δ (ppm) 150.6, 137.8, 126.3, 119.7, 50.4, 34.7, 31.5, 30.6, 16.4, 15.6 HRMS (ESI): m/z calculated for: $[C_{28}H_{37}N_2]^+$ $[M+H]^+$: 401.2952; found 401.2952.

***Erythro*-4,5-*bis*(4-(trifluoromethyl)phenyl)octanedinitrile (7d)**

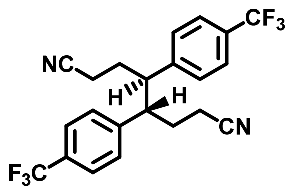

4-Trifluoromethylstyrene (**7**, 220 mg, 1.58 mmol, 1.0 eq), $BrCH_2CN$ (**1a**, 228 mg, 1.90 mmol, 1.2 eq) and **[Cu(Me$_6$tren)](ClO_4)$_2$** (5 mg, 0.010 mmol, 0.0075 eq) in 50 mL of anhydrous CH_3CN were used. The reaction was stopped after 13 h of electrolysis at -900 mV vs $Fc^{0/+}$. The residue was purified by column chromatography (Silica gel, 5%-50% EtOAc in pet. ether) to provide a mixture of *erythro* **(6d)** and *threo* **(6d')** isomers as a colorless solid (280 mg, 83%, ratio of 2:1, respectively). The *erythro* isomer was crystallized by slow diffusion of hexane into an acetone solution to give colorless crystals suitable for XRD. m.p. 217˚C-219˚C. ^{1}H-NMR (500 MHz, $CDCl_3$): δ (ppm) 7.72-7.69 (m, 4H), 7.45-7.43 (m, 4H), 3.09-3.02 (m, 2H), 2.14-2.06 (m, 2H), 1.86-1.78 (m, 2H), 1.72-1.64 (m, 3H). ^{13}C-NMR (125 MHz, CDCl3): δ (ppm) 144.5, 130.7, 130.5, 128.5, 126.7, 118.7, 50.5, 30.2, 15.5. HRMS (ESI): m/z calculated for: $[C_{22}H_{19}F_6N_2]^+$ $[M+H]^+$: 425.1447; found 425.1449.

***Threo*-4,5-*bis*(4-(*tert*-butyl)phenyl)octanedinitrile (8d)**

4-Chlorostyrene (**8**, 240 mg, 1.73 mmol, 1.0 eq), $BrCH_2CN$ (**1b**, 190 mg, 1.58 mmol, 1.5 eq) and **[Cu(Me$_6$tren)](ClO_4)$_2$** (5 mg, 0.01 mmol, 0.0057 eq) in 50 mL of anhydrous CH_3CN were used. The reaction was stopped after 12 h of electrolysis at -900 mV vs $Fc^{0/+}$. The residue was purified by column chromatography (Silica gel, 10%-20% EtOAc in pet. ether) to provide a mixture of *eythro* **(6d)** and *threo* **(6d')** isomers as a colorless solid (217 mg, 70%, ratio of 1:0.85, respectively). The *erythro* isomer was crystallized by vapor diffusion of hexane into an acetone solution to afford colorless crystals suitable for XRD. m.p. 232˚C-233˚C ^{1}H-NMR (500 MHz, $CDCl_3$): δ (ppm) 7.46–7.34 (m, 4H), 7.24–7.19 (m, 4H), 2.92 – 2.82 (m, 2H), 2.07 (m, 2H), 1.82 (m, 2H), 1.69 (m, 2H), 1.60 (m, 2H). ^{13}C-NMR (125 MHz, CDCl3): δ (ppm) 139.0, 133.8, 129.8, 129.3, 118.9, 50.1, 30.2, 15.5. HRMS (ESI): m/z calculated for: $[C_{20}H_{19}Cl_2N_2]^+$ $[M+H]^+$: 357.0920; found 357.0923.

8. NMR Spectra

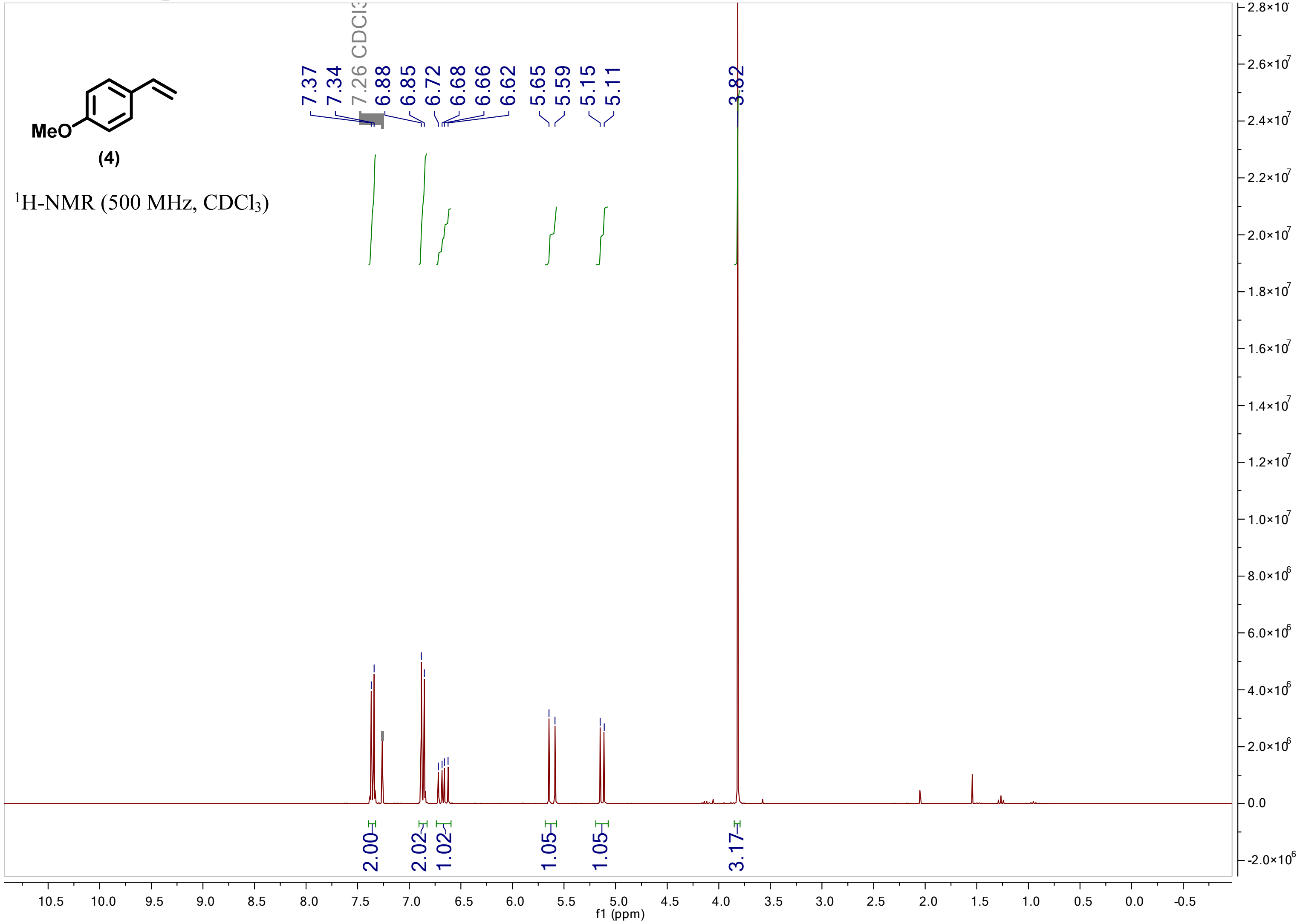

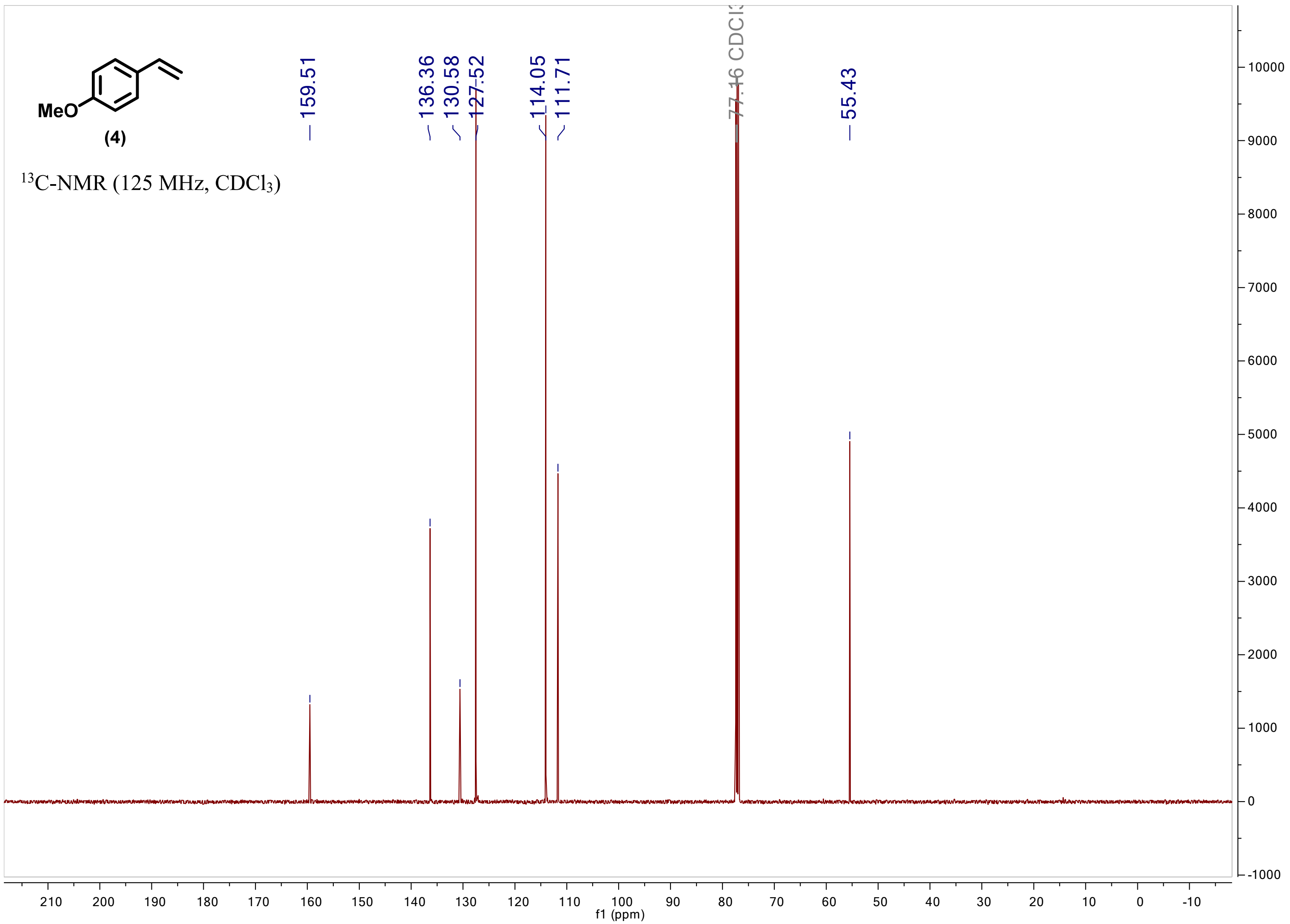

^{13}C-NMR (125 MHz, $CDCl_3$)

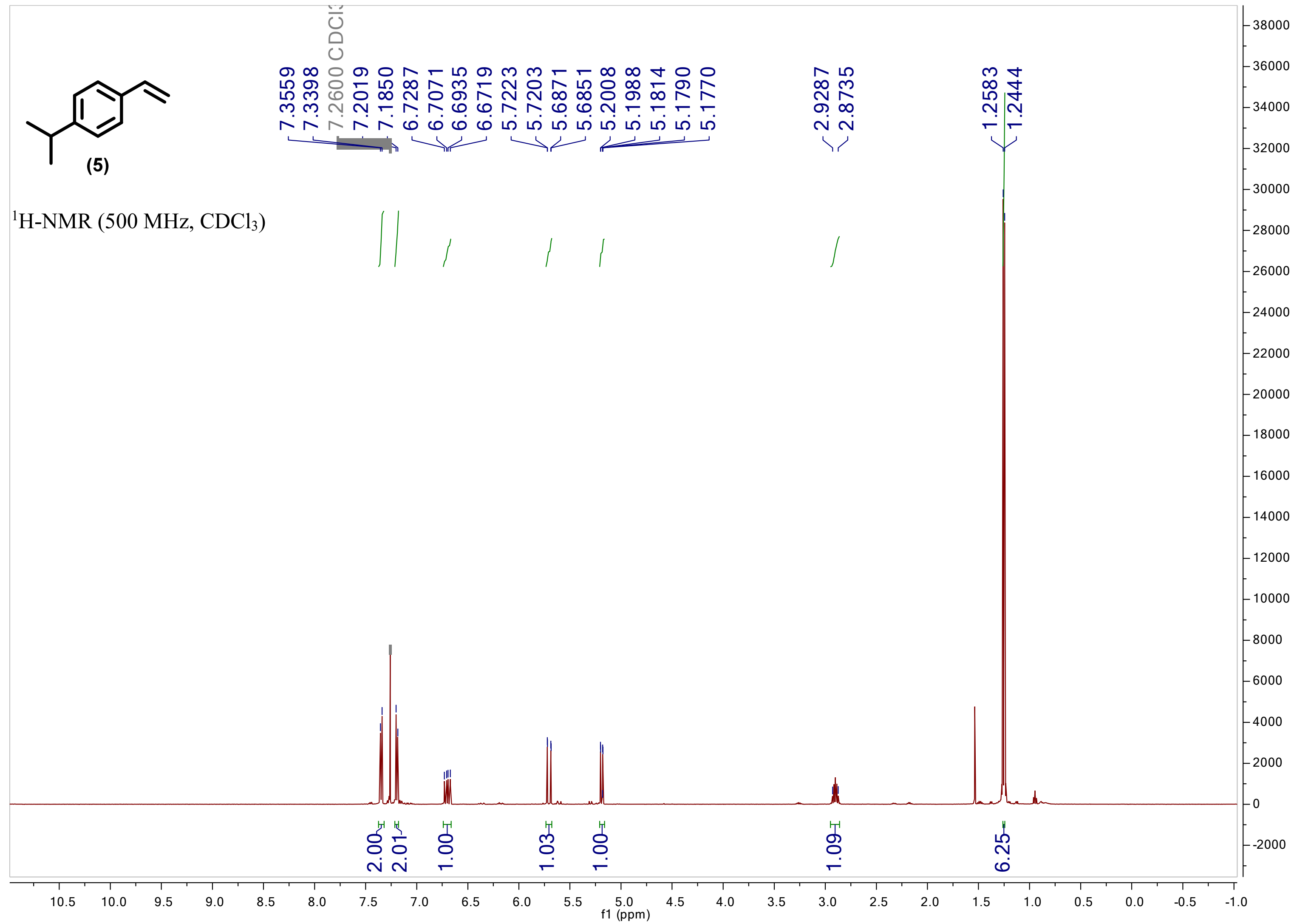

(5)
1H-NMR (500 MHz, CDCl3)
7.3559
7.3398
7.2600 CDCl3
7.2019
7.1850
6.7287
6.7071
6.6935
6.6719
5.7223
5.7203
5.6871
5.6851
5.2008
5.1988
5.1814
5.1790
5.1770
2.9287
2.8735
1.2583
1.2444
2.00
2.01
1.00
1.03
1.00
1.09
6.25
f1 (ppm)

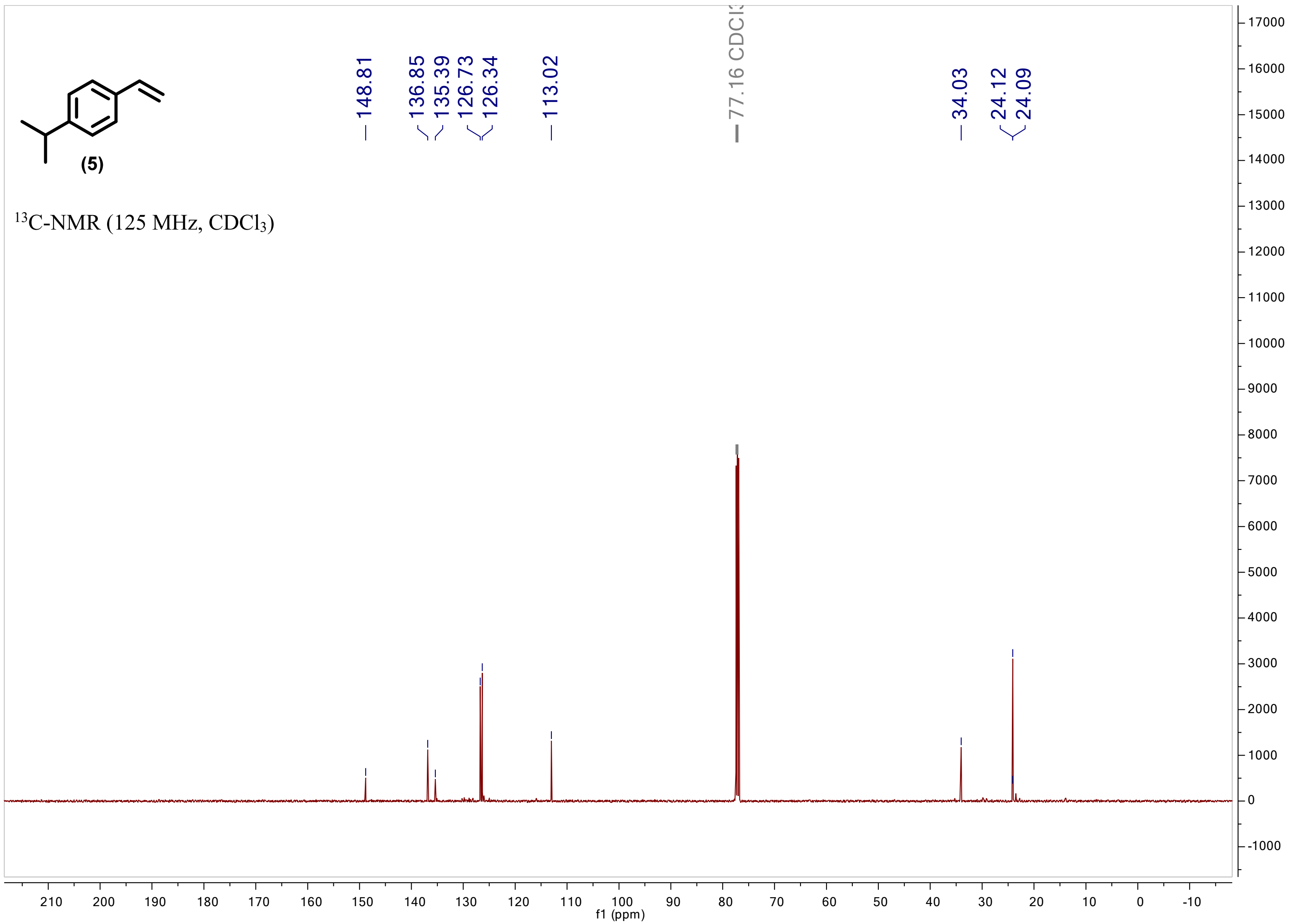

(5)
¹³C-NMR (125 MHz, CDCl₃)
148.81
136.85
135.39
126.73
126.34
113.02
77.16 CDCl3
34.03
24.12
24.09
f1 (ppm)

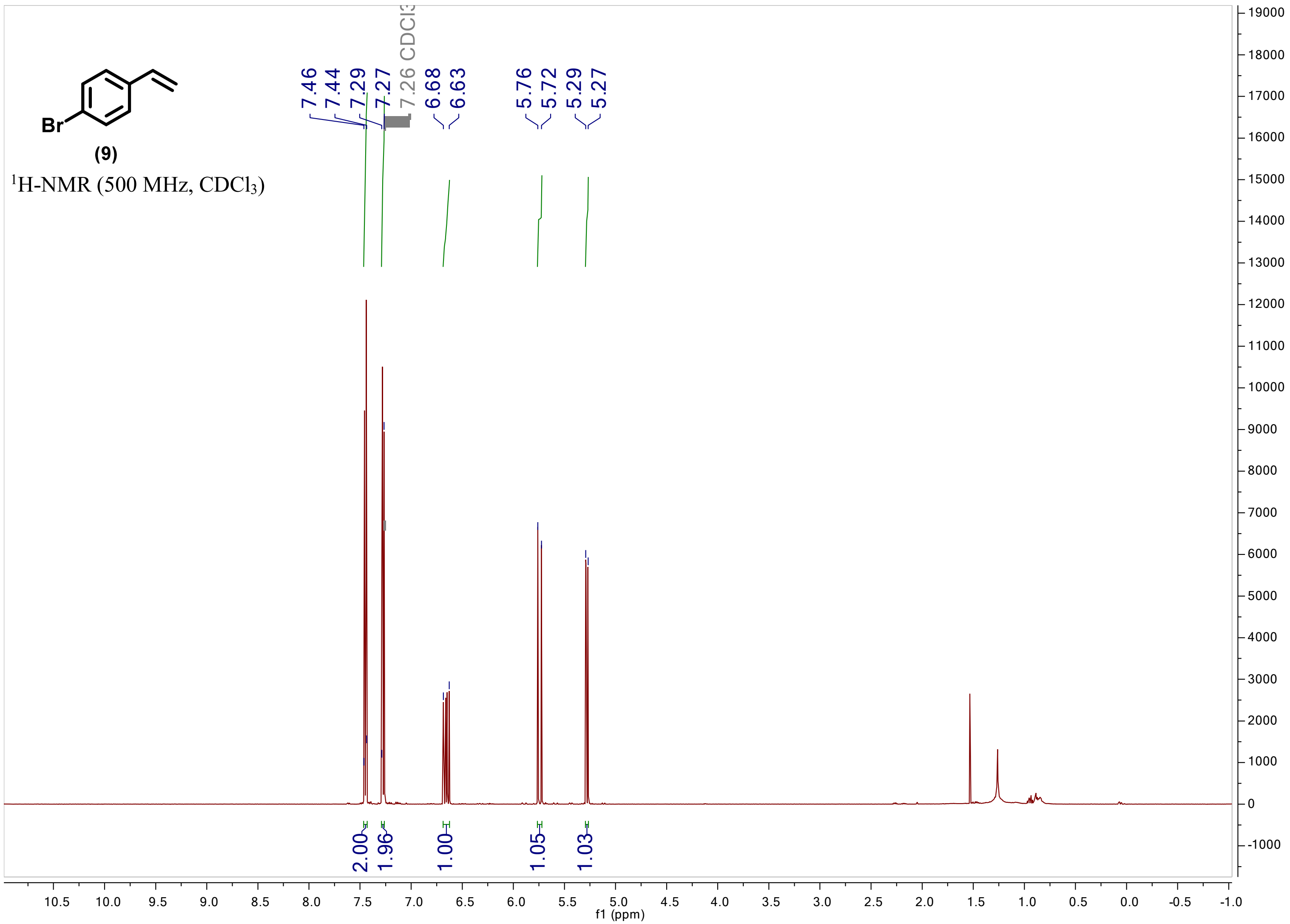

(9)

^{1}H-NMR (500 MHz, $CDCl_3$)

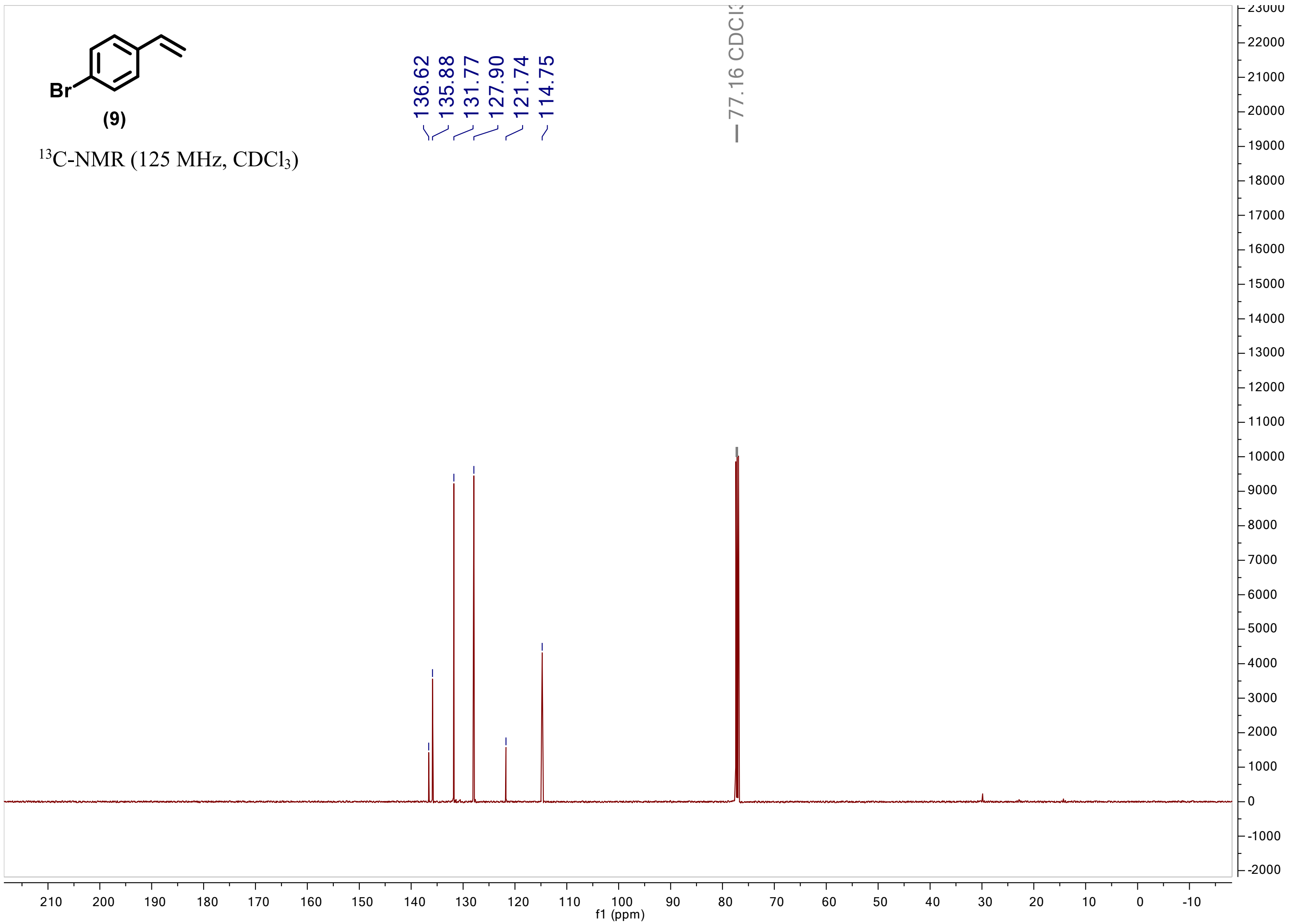

^{13}C-NMR (125 MHz, $CDCl_3$)

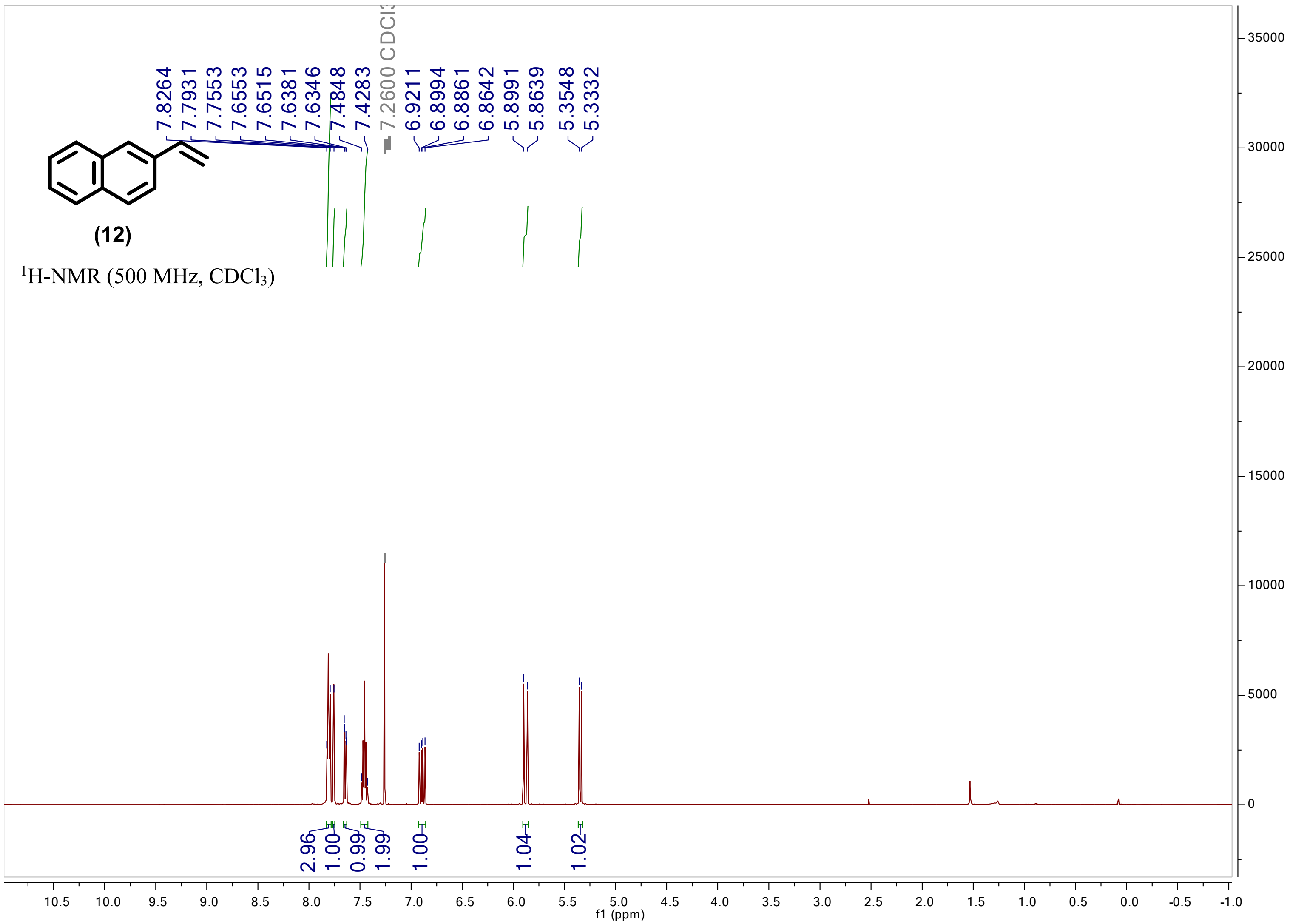

(12)
¹H-NMR (500 MHz, CDCl3)
7.8264
7.7931
7.7553
7.6553
7.6515
7.6381
7.6346
7.4848
7.4283
7.2600 CDCl3
6.9211
6.8994
6.8861
6.8642
5.8991
5.8639
5.3548
5.3332
2.96
1.00
0.99
1.99
1.00
1.04
1.02
f1 (ppm)

(12)

^{13}C-NMR (125 MHz, CDCl$_3$)

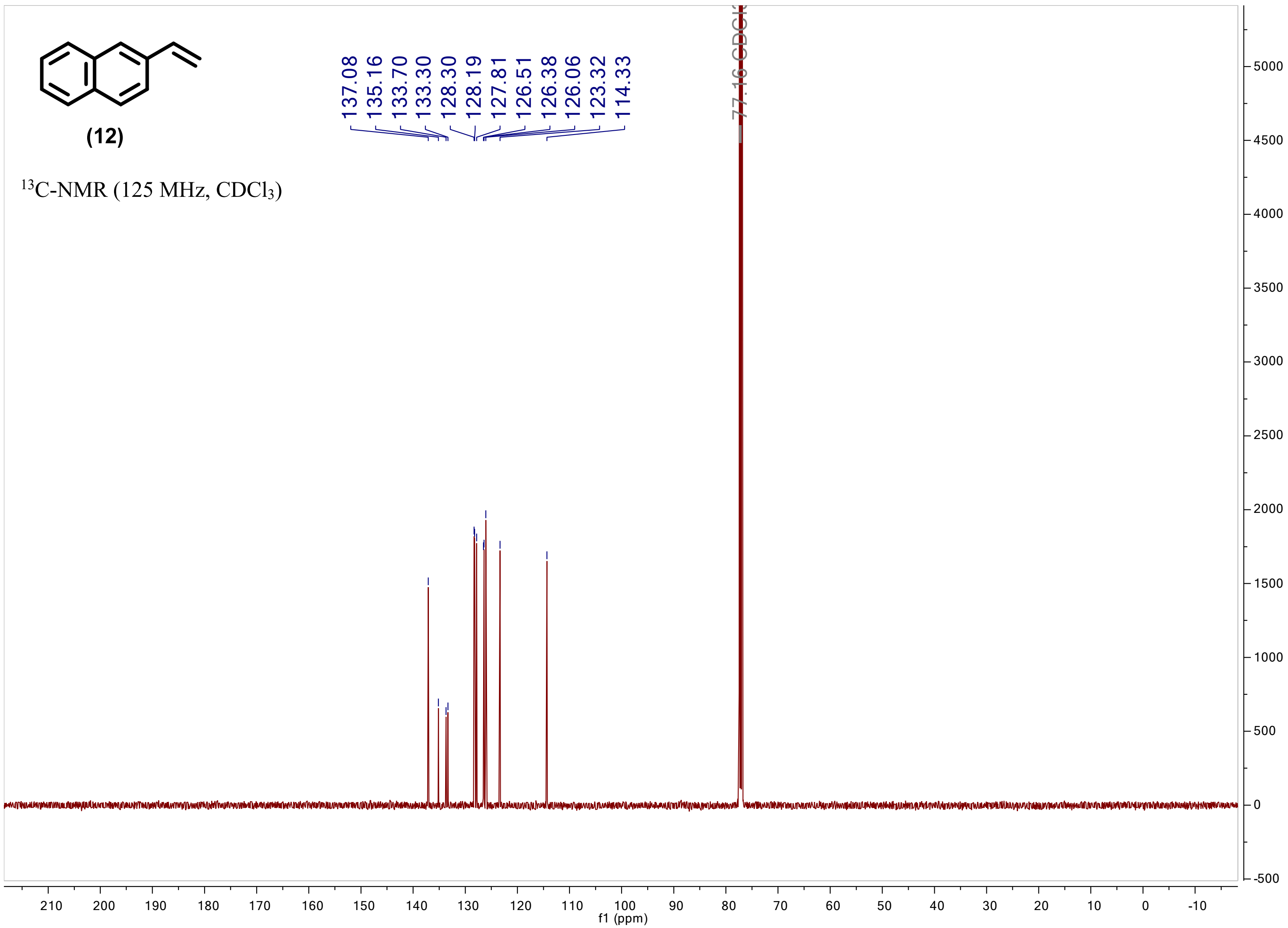

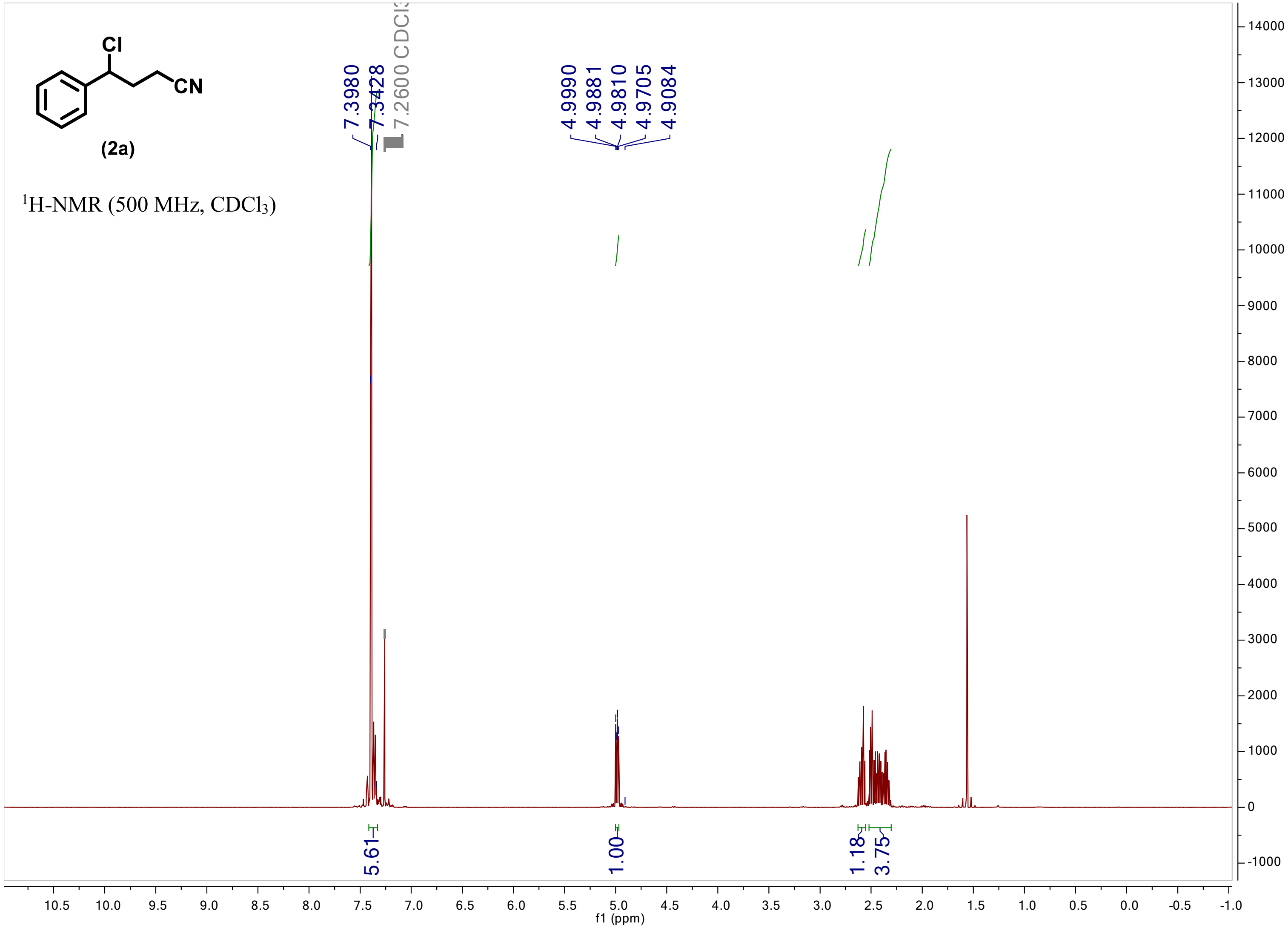
Cl
CN
(2a)
¹H-NMR (500 MHz, CDCl₃)
7.3980
7.3428
7.2600 CDCl3
4.9990
4.9881
4.9810
4.9705
4.9084
5.61
1.00
1.18
3.75
f1 (ppm)

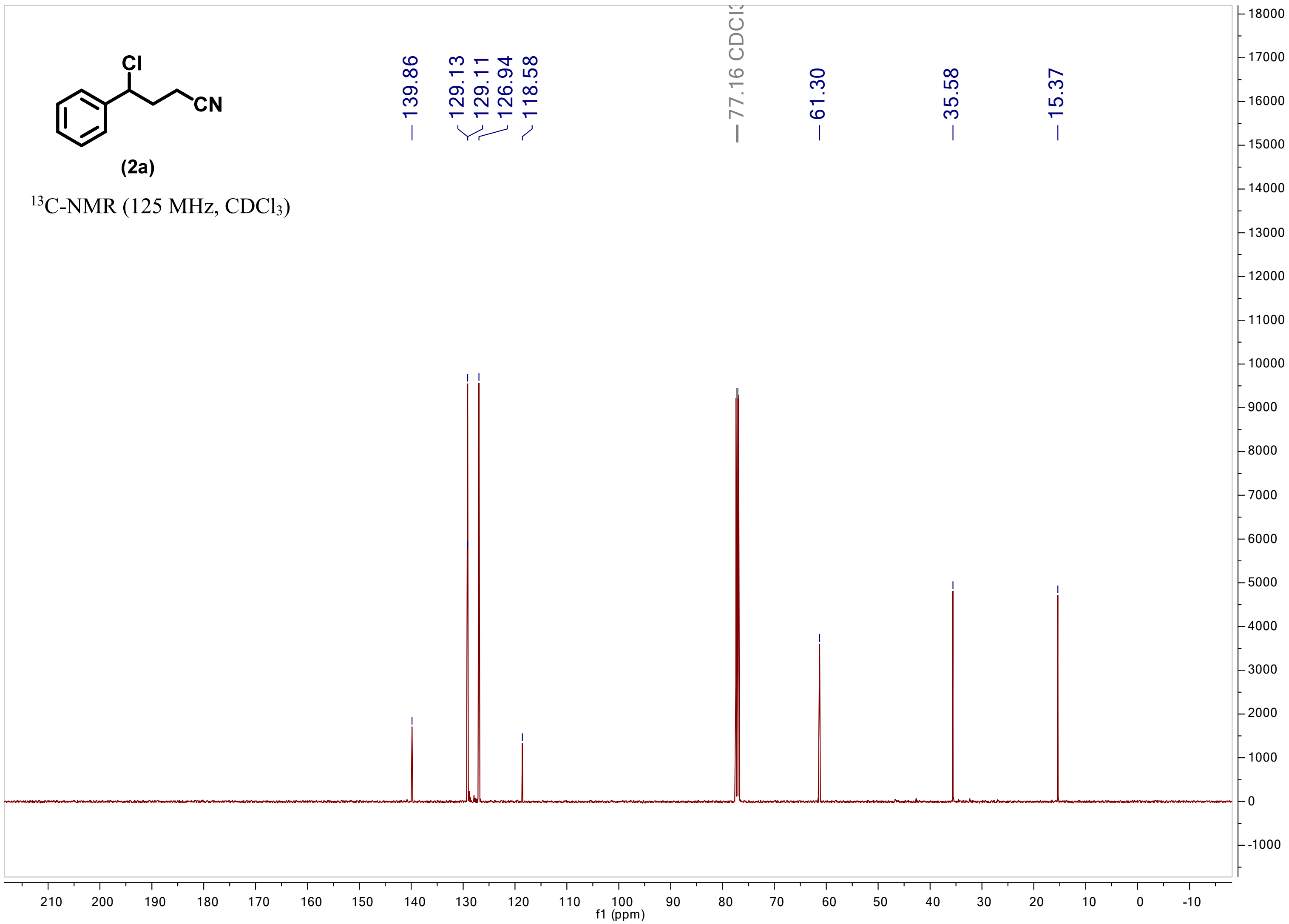

(2a)

^{13}C-NMR (125 MHz, $CDCl_3$)

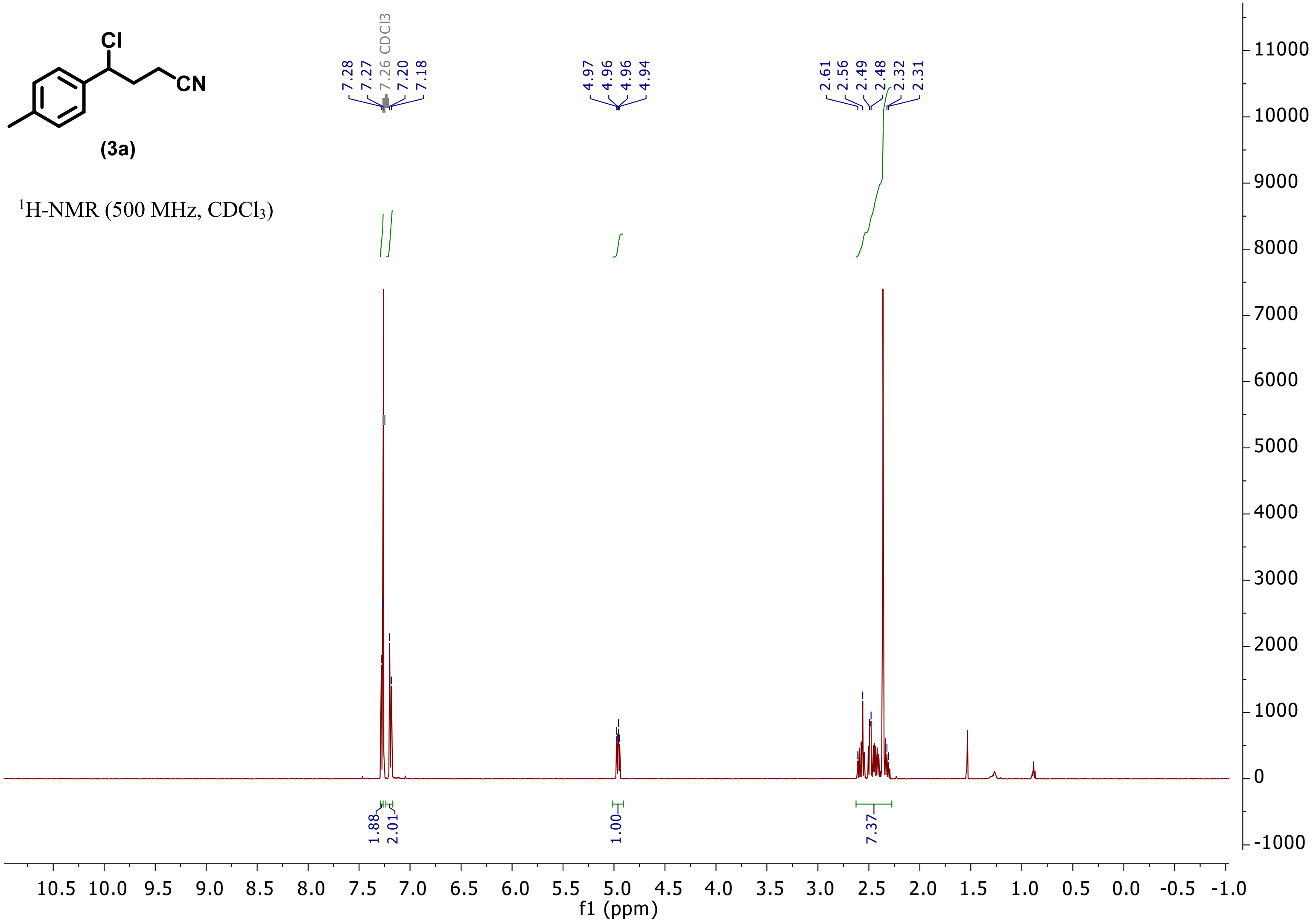

(3a)

^{1}H-NMR (500 MHz, $CDCl_3$)

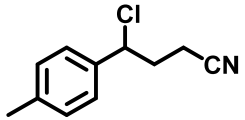

(3a)

^{13}C-NMR (125 MHz, $CDCl_3$)

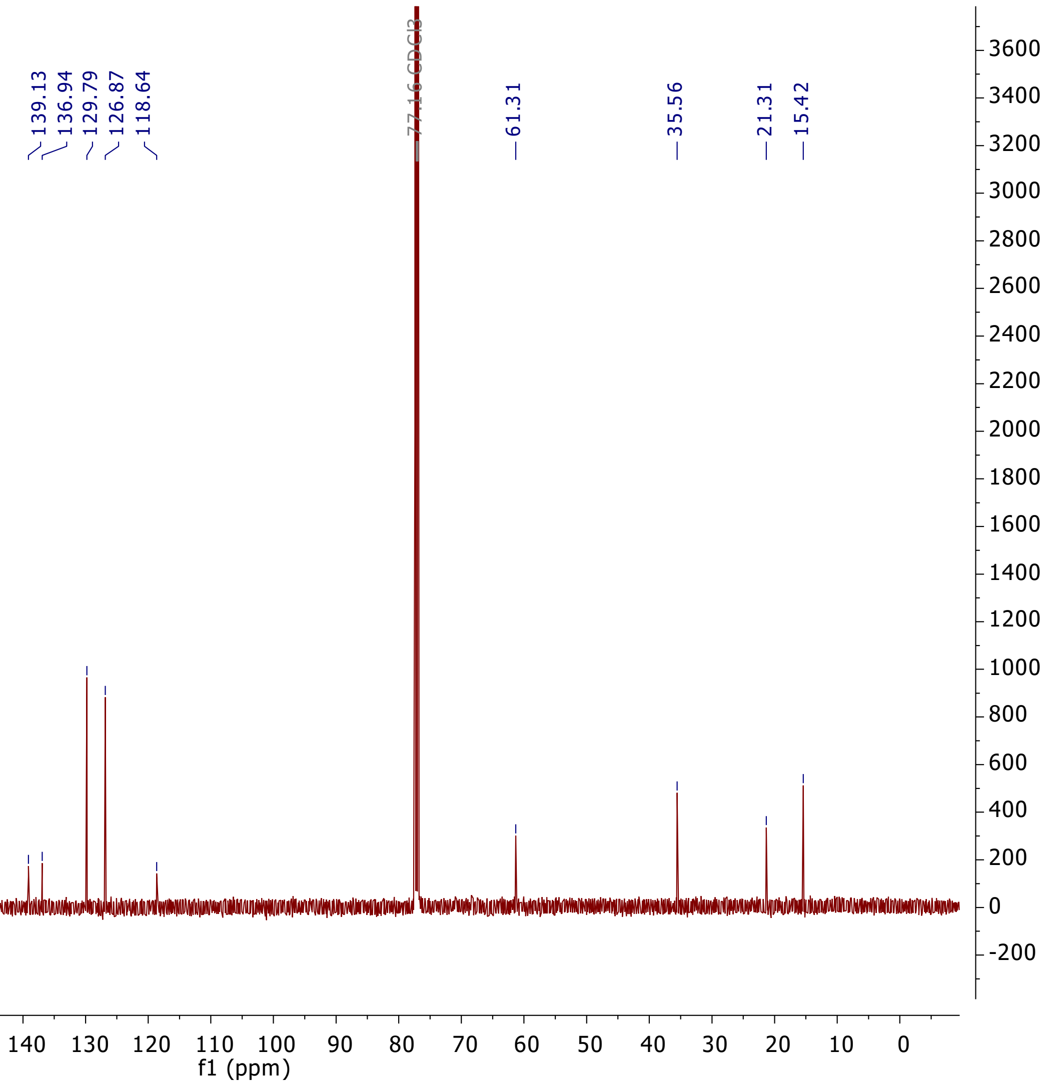

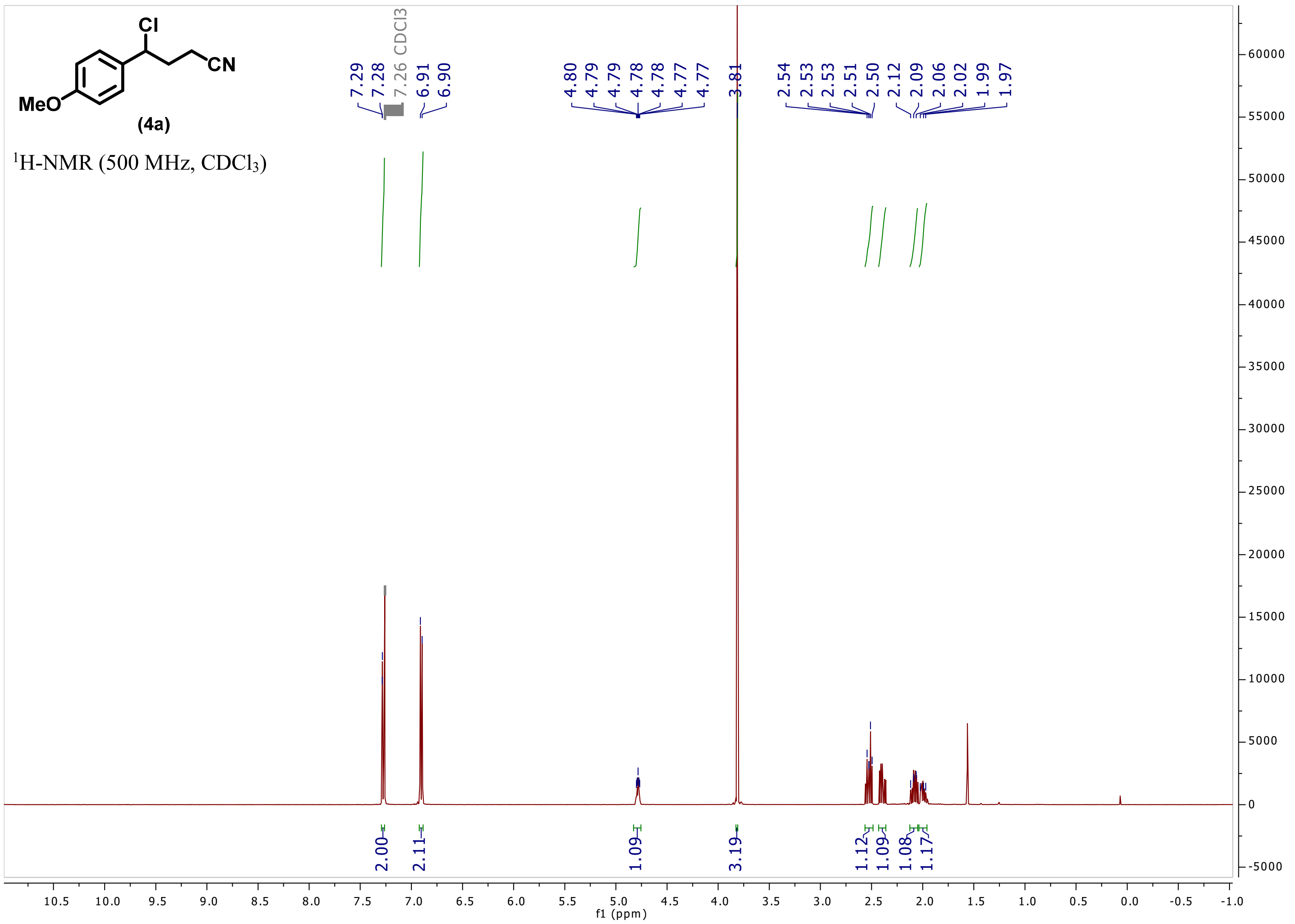
Cl
CN
MeO
(4a)
^{1}H-NMR (500 MHz, $CDCl_3$)
7.29
7.28
7.26 CDCl3
6.91
6.90
4.80
4.79
4.79
4.78
4.78
4.77
4.77
3.81
2.54
2.53
2.53
2.51
2.50
2.12
2.09
2.06
2.02
1.99
1.97
2.00
2.11
1.09
3.19
1.12
1.09
1.08
1.17
f1 (ppm)

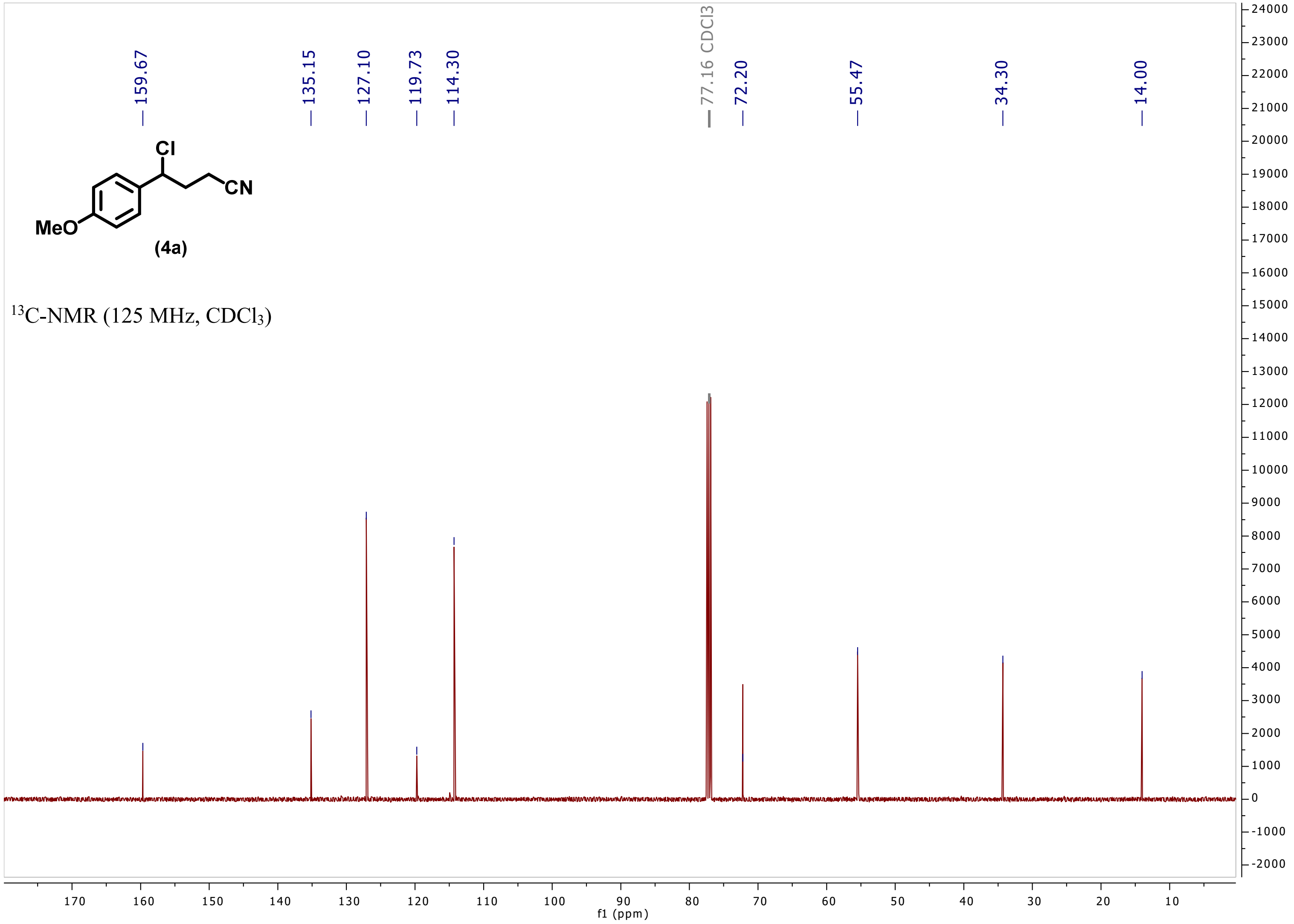

159.67
135.15
127.10
119.73
114.30
77.16 CDCl3
72.20
55.47
34.30
14.00
Cl
CN
MeO
(4a)
^{13}C-NMR (125 MHz, $CDCl_3$)
f1 (ppm)

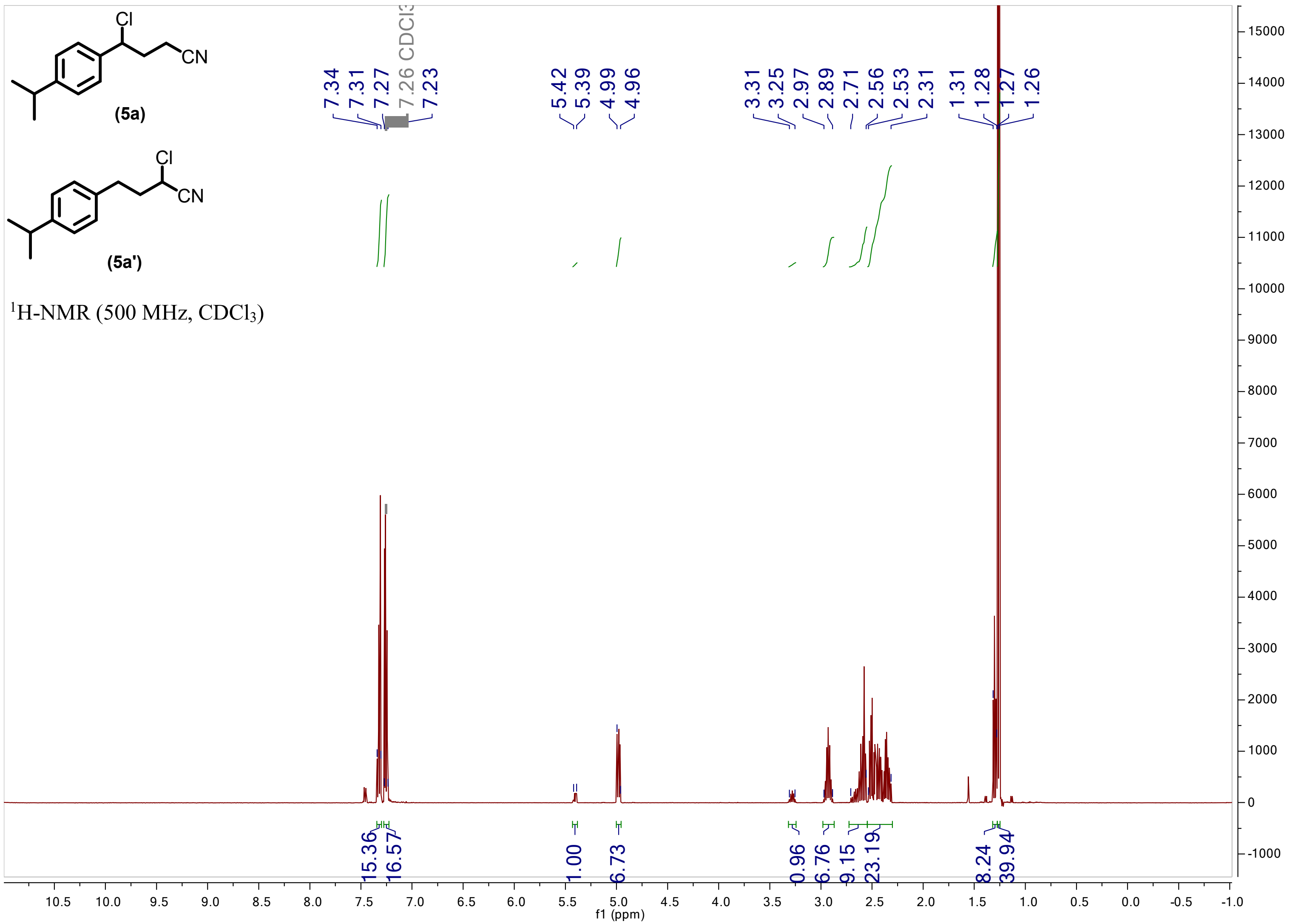

(5a)
(5a')
^{1}H-NMR (500 MHz, CDCl$_3$)
7.34 7.31 7.27 7.26 CDCl3 7.23
5.42 5.39 4.99 4.96
3.31 3.25 2.97 2.89 2.71 2.56 2.53 2.31
1.31 1.28 1.27 1.26
15.36 16.57 1.00 6.73 0.96 6.76 9.15 23.19 8.24 39.94
f1 (ppm)

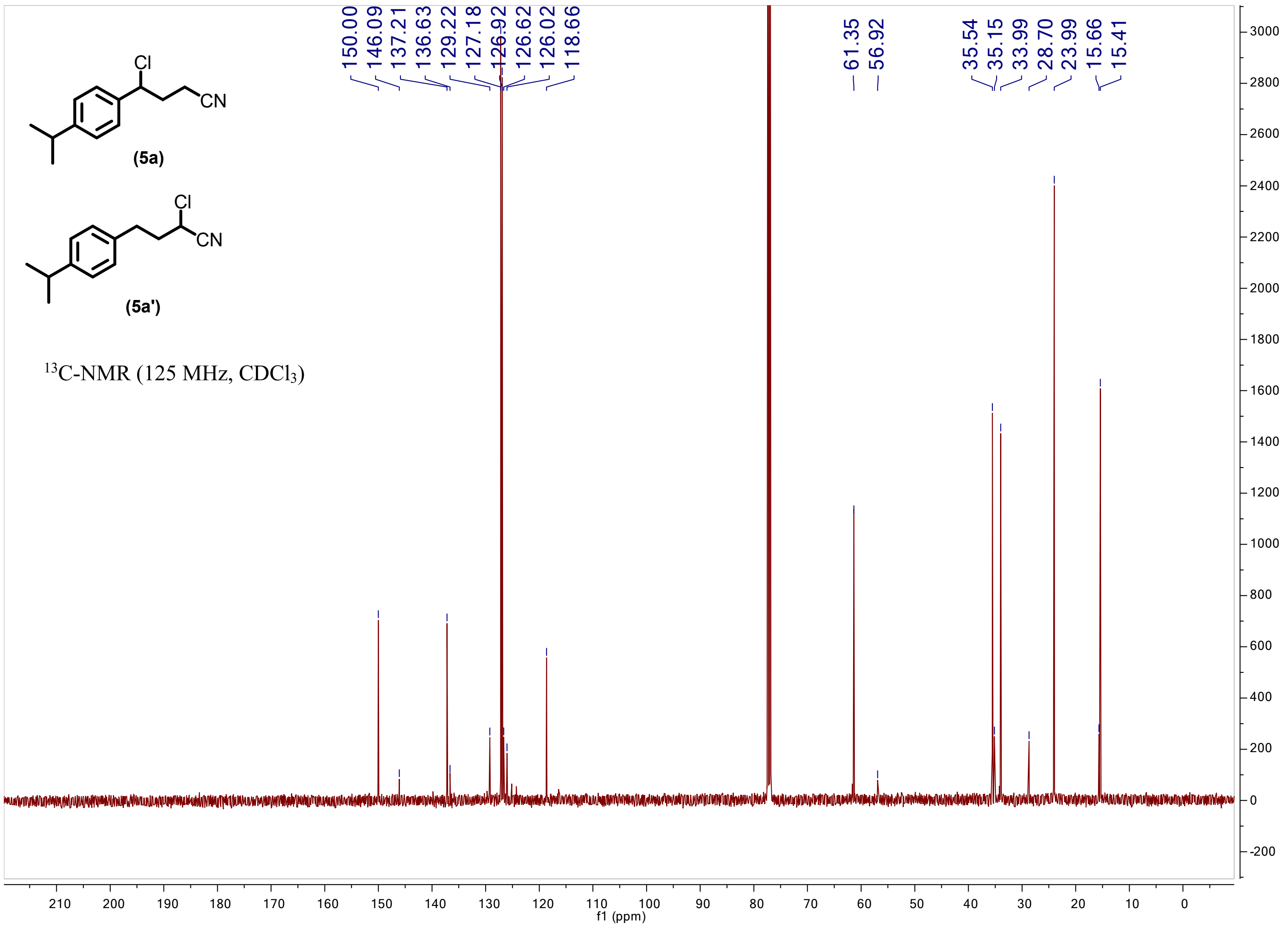

Cl
CN
(5a)
Cl
CN
(5a')
13C-NMR (125 MHz, CDCl3)
150.00
146.09
137.21
136.63
129.22
127.18
126.92
126.62
126.02
118.66
61.35
56.92
35.54
35.15
33.99
28.70
23.99
15.66
15.41
f1 (ppm)

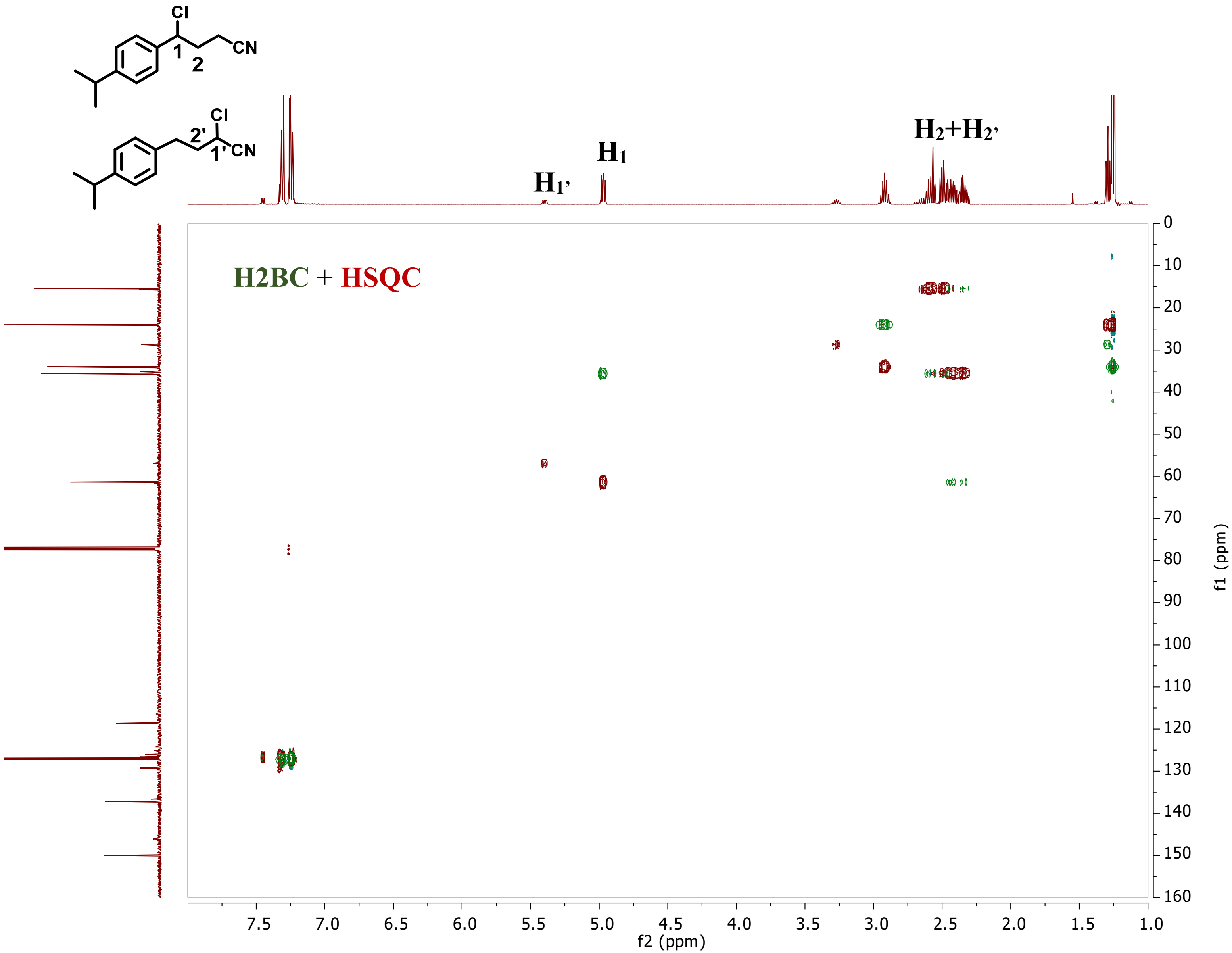

Cl
1
2
CN
Cl
2'
1'
CN
H1'
H1
H2+H2'
H2BC + HSQC
f2 (ppm)
f1 (ppm)

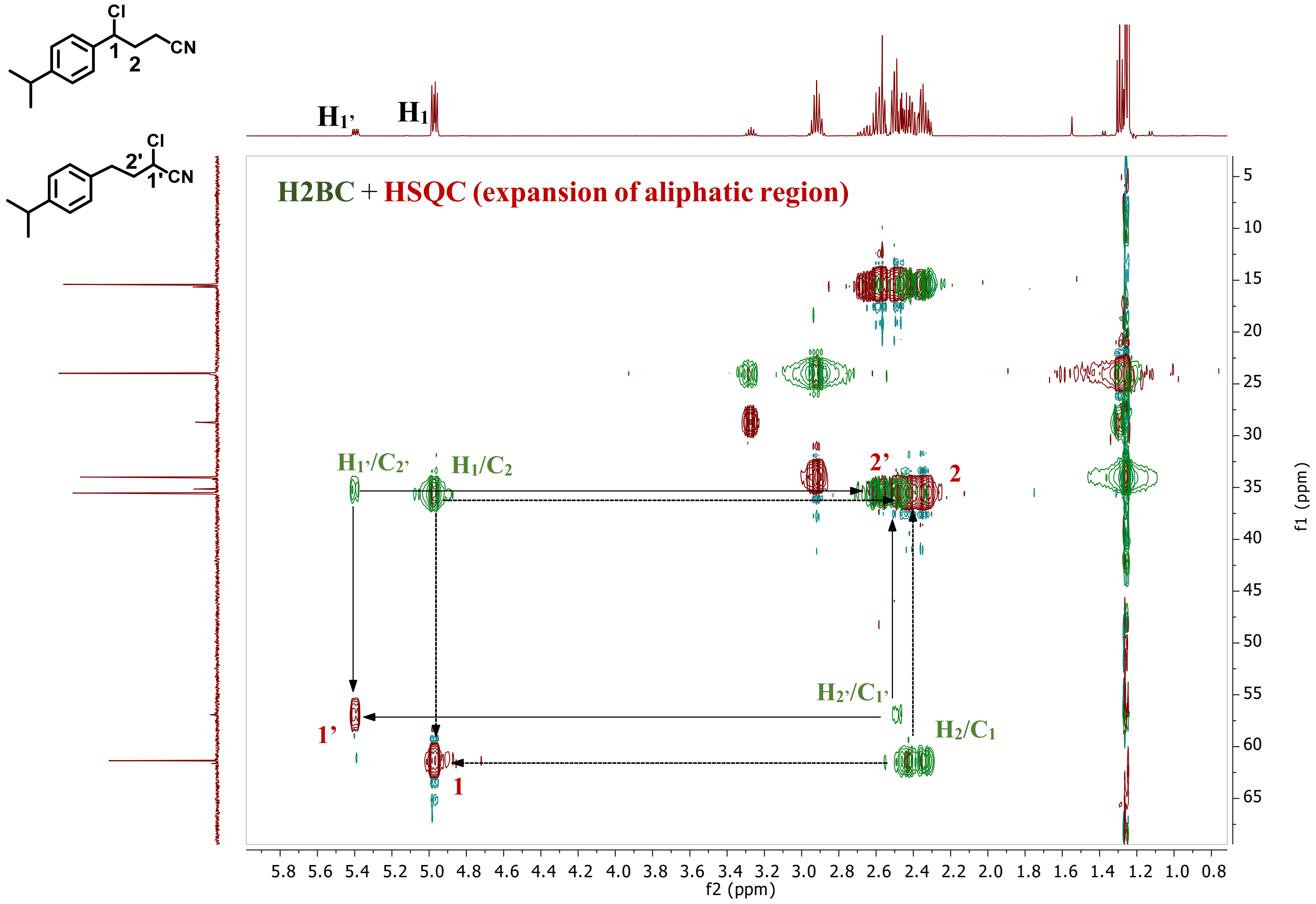

Cl
1
2
CN
Cl
2'
1'
CN
H1'
H1
H2BC + HSQC (expansion of aliphatic region)
H1'/C2'
H1/C2
2'
2
H2'/C1'
H2/C1
1'
1
f2 (ppm)
f1 (ppm)

(6a)

^{1}H-NMR (500 MHz, $CDCl_3$)

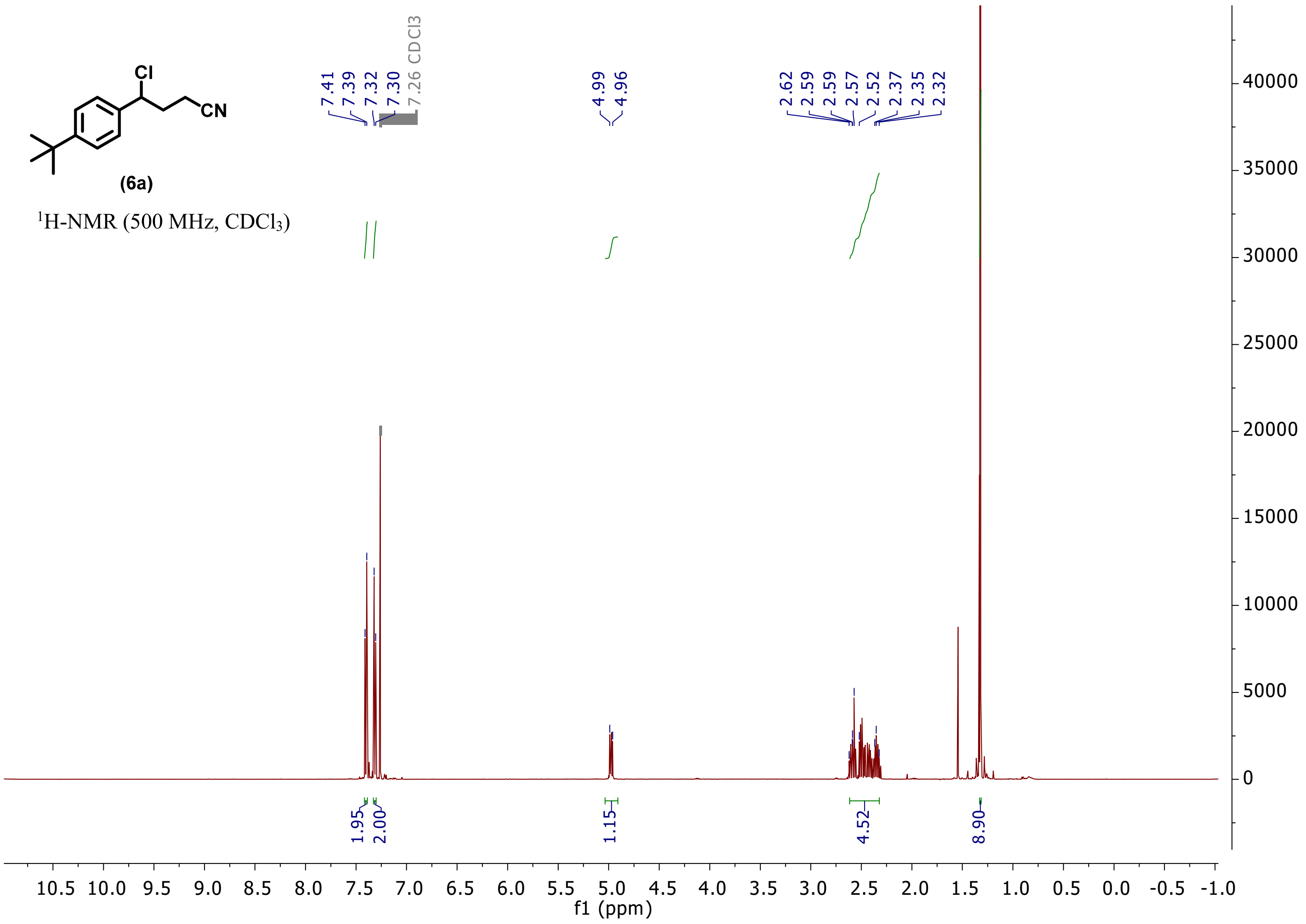

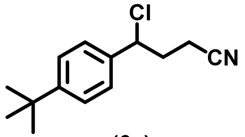

(6a)

^{13}C-NMR (125 MHz, $CDCl_3$)

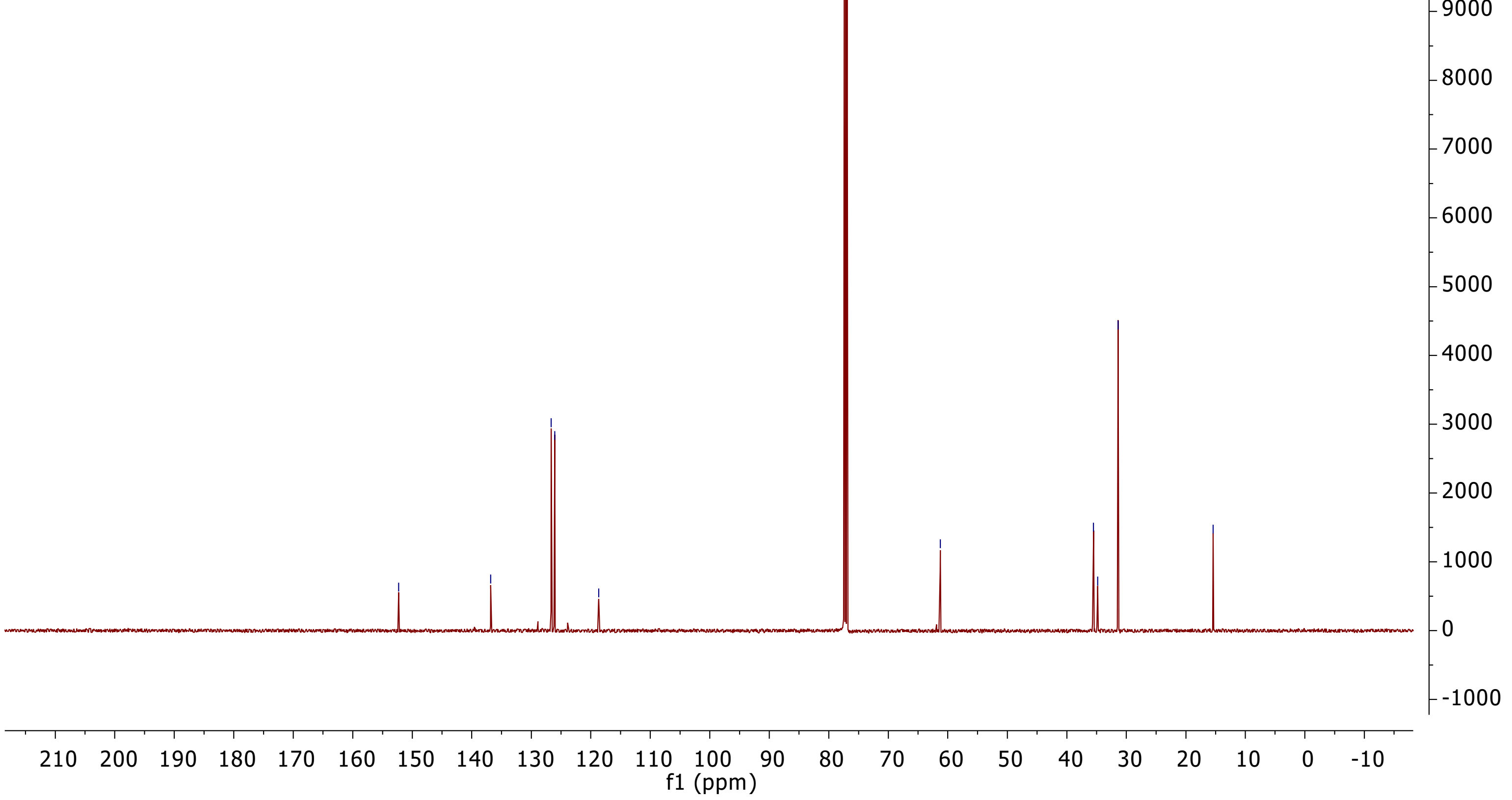

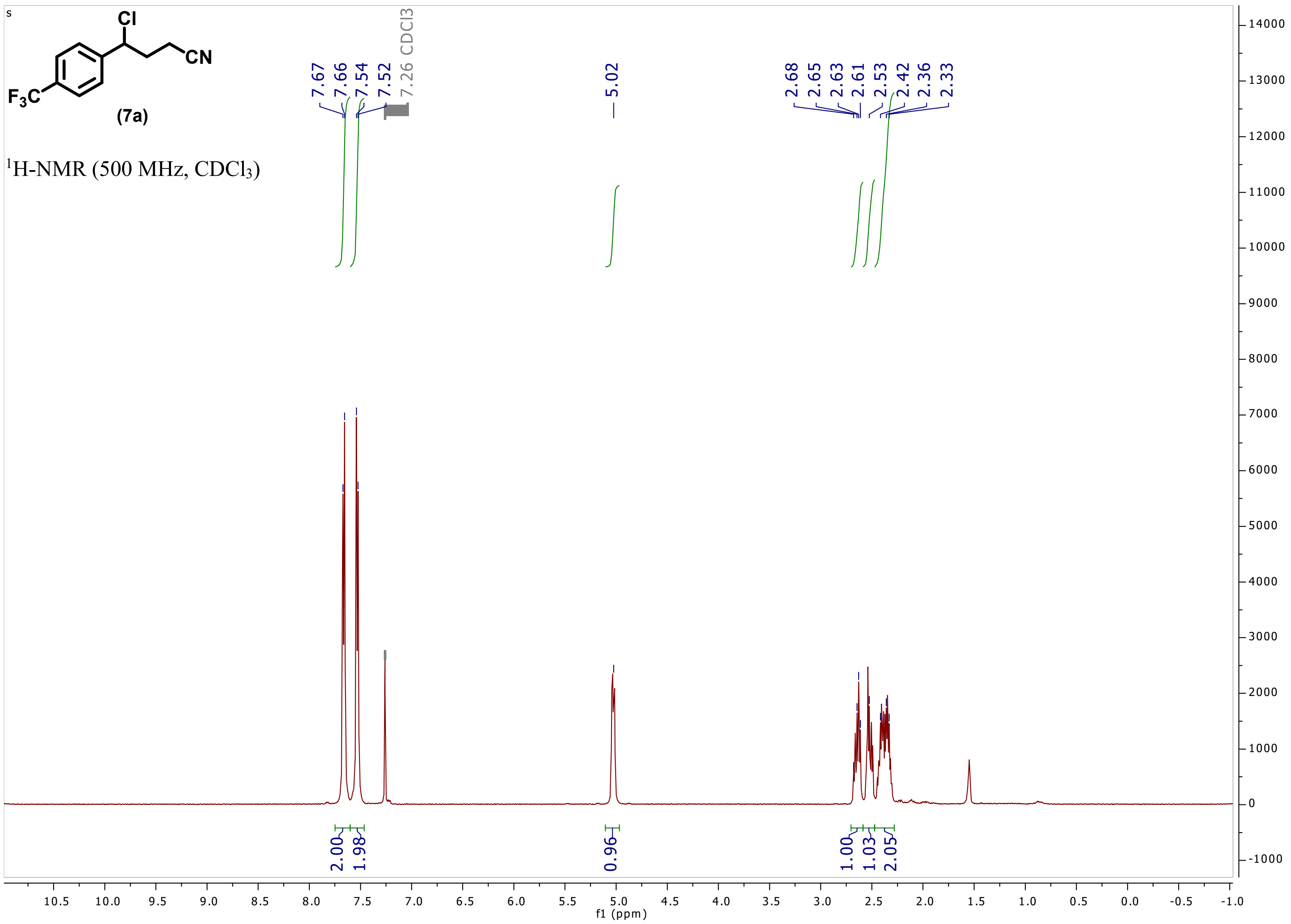
Cl
CN
F3C
(7a)
1H-NMR (500 MHz, CDCl3)
7.67
7.66
7.54
7.52
7.26 CDCl3
5.02
2.68
2.65
2.63
2.61
2.53
2.42
2.36
2.33
2.00
1.98
0.96
1.00
1.03
2.05
f1 (ppm)

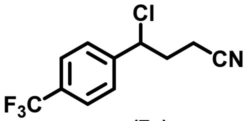

(7a)

^{13}C-NMR (125 MHz, $CDCl_3$)

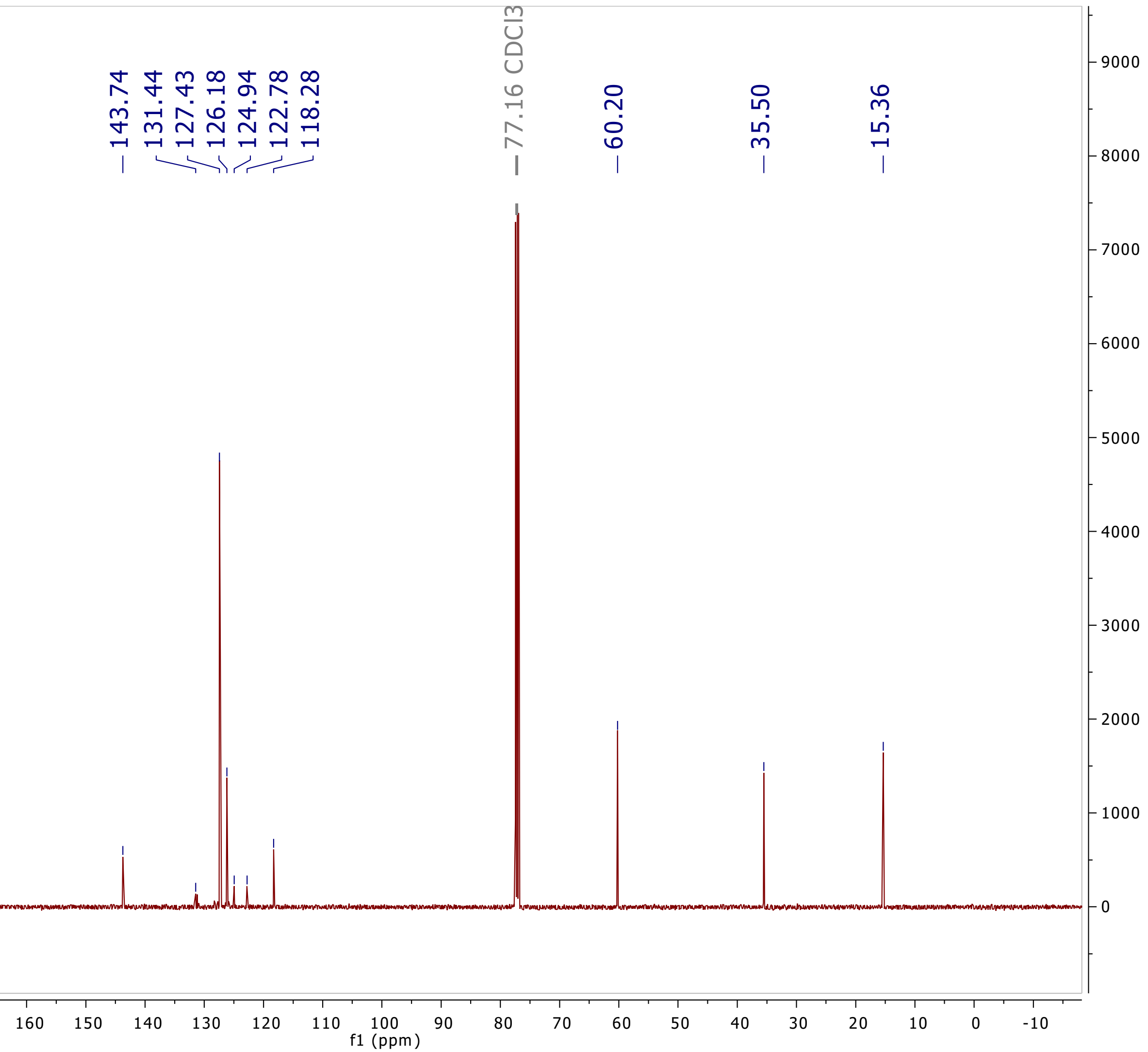

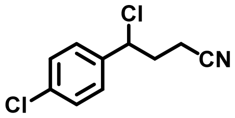

(8a)

^{1}H-NMR (500 MHz, $CDCl_3$)

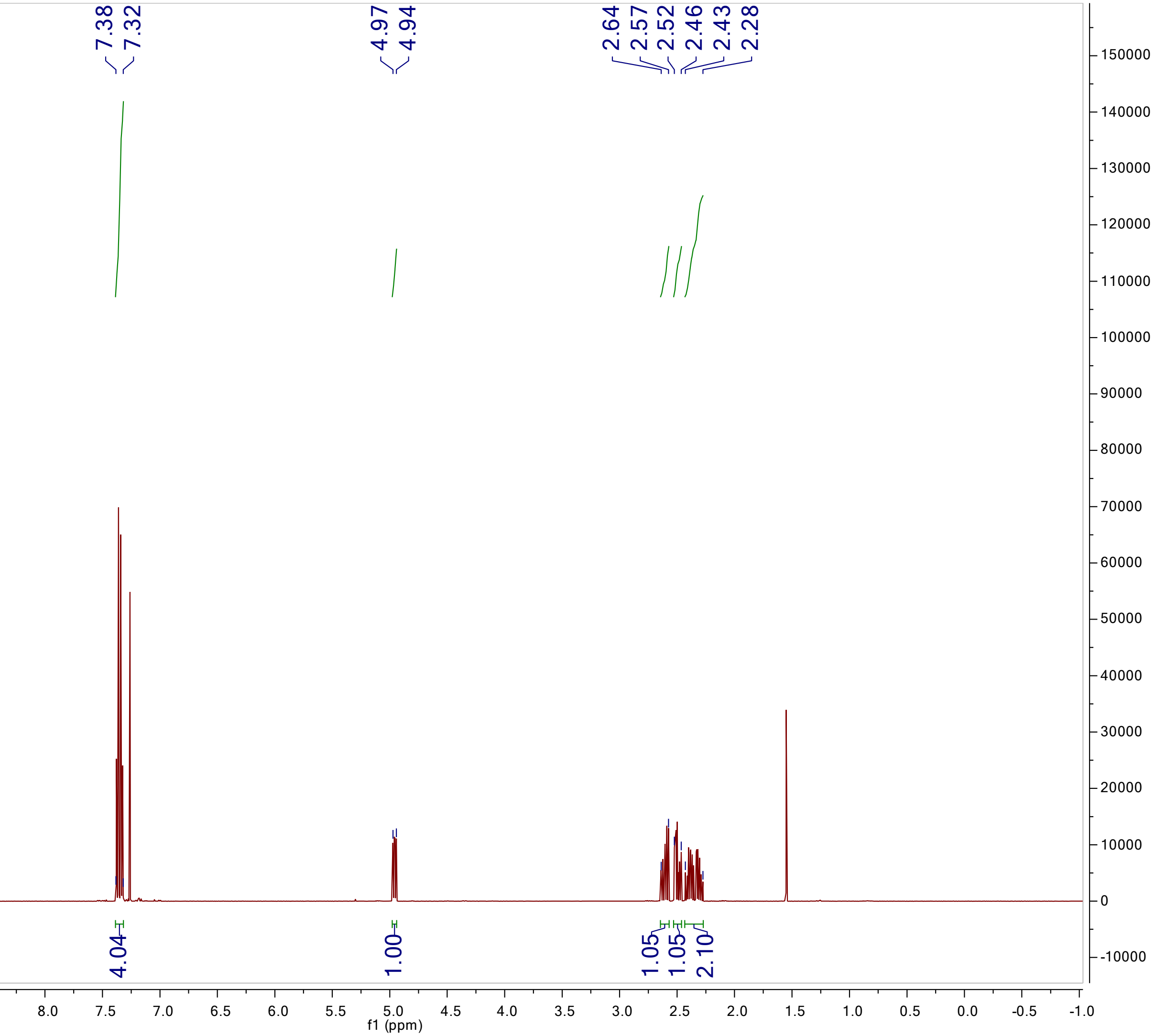

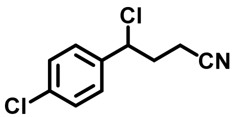

(8a)

^{13}C-NMR (125 MHz, $CDCl_3$)

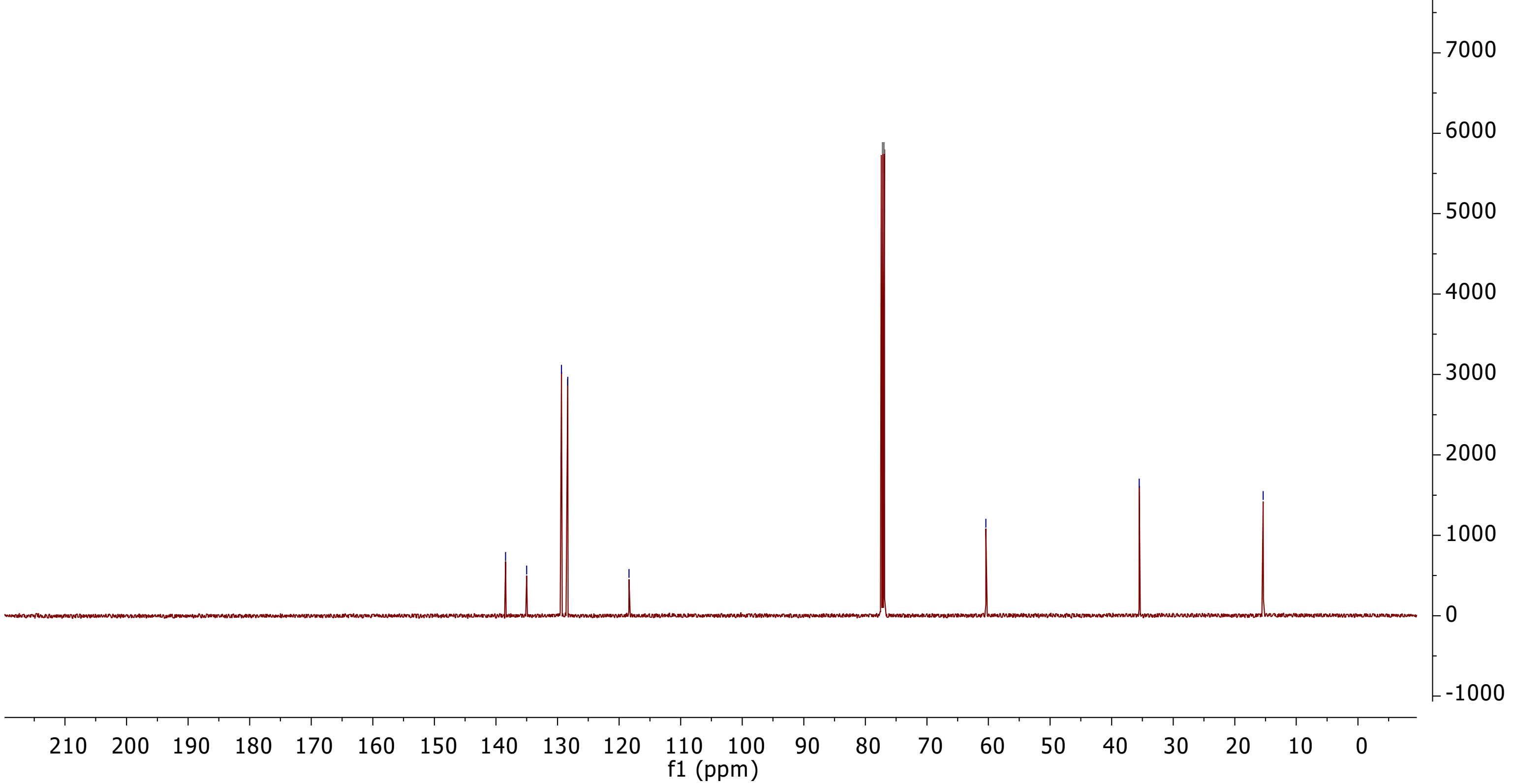

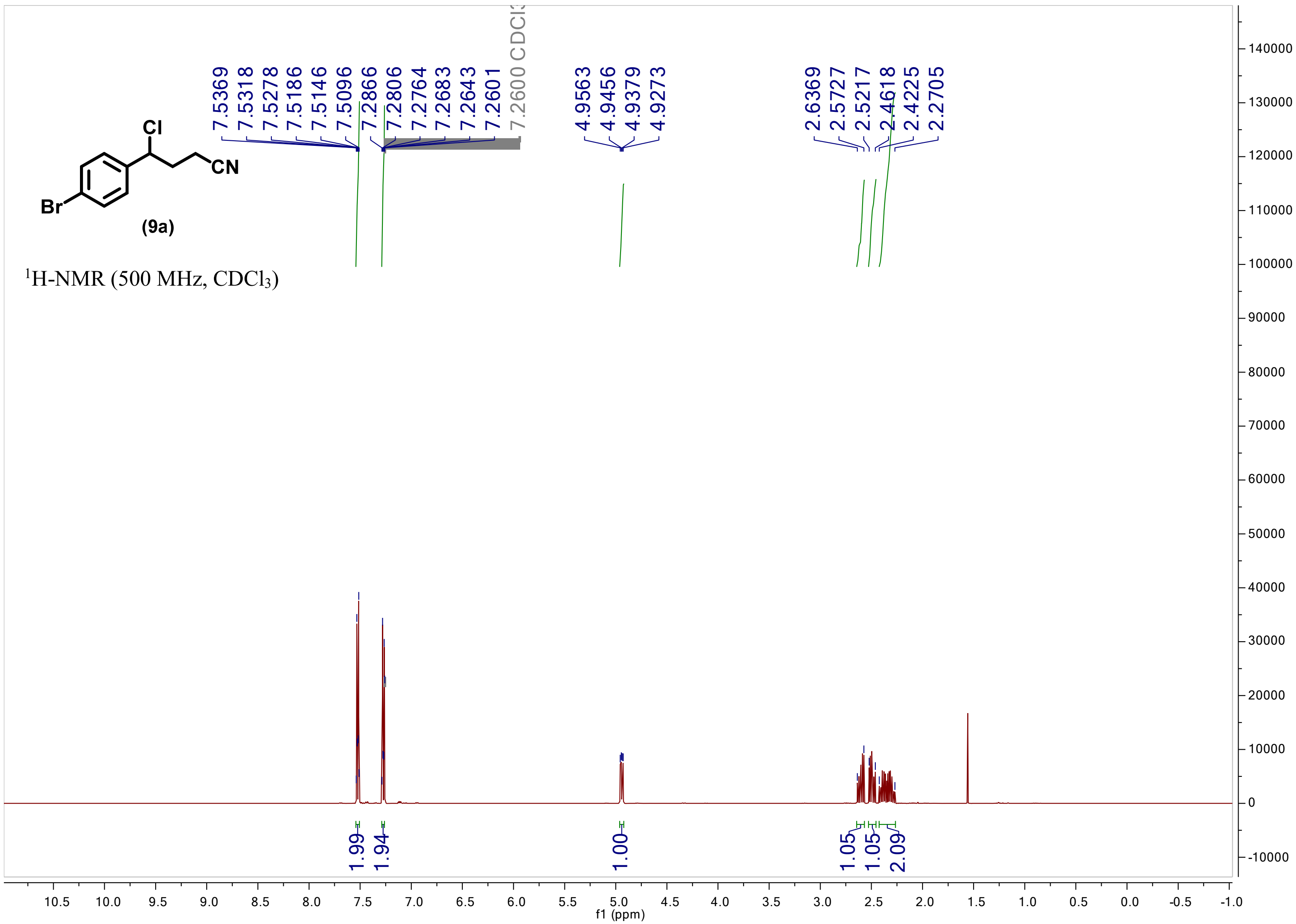

(9a)

^{1}H-NMR (500 MHz, $CDCl_3$)

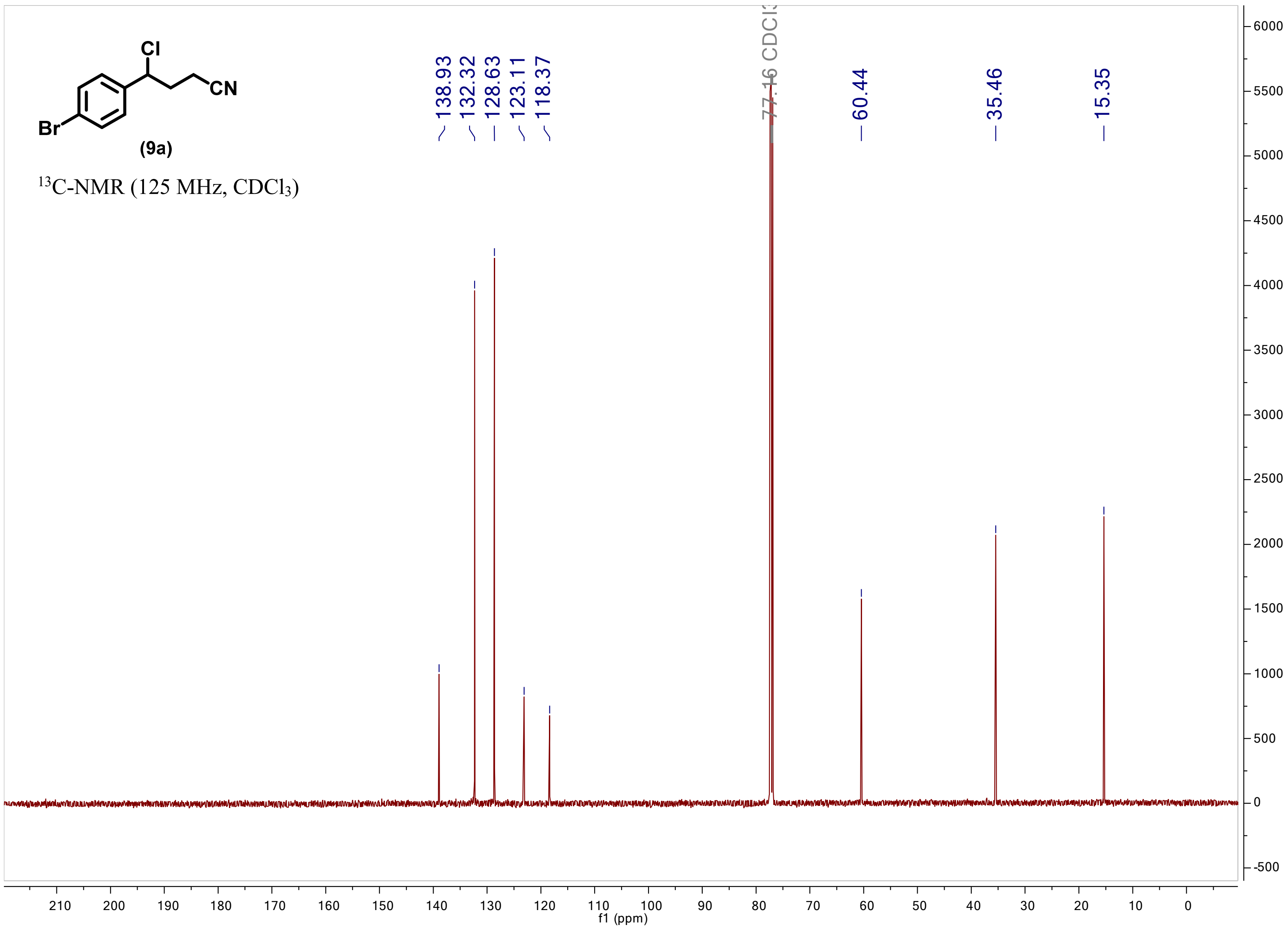
Cl
CN
Br
(9a)
^{13}C-NMR (125 MHz, CDCl$_3$)
138.93
132.32
128.63
123.11
118.37
77.16 CDCl3
60.44
35.46
15.35
f1 (ppm)

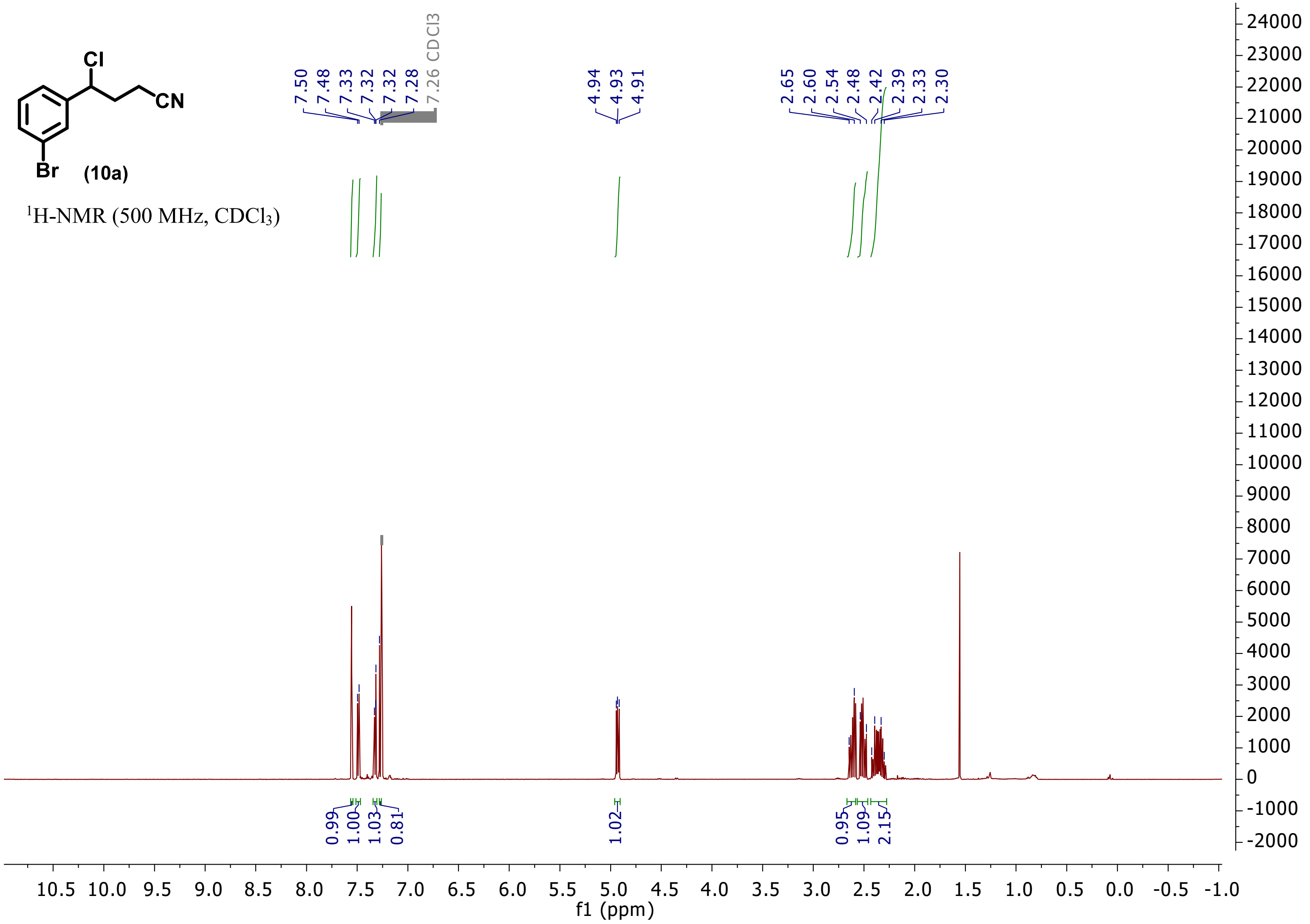

Cl
CN
Br
(10a)
¹H-NMR (500 MHz, CDCl3)
7.50
7.48
7.33
7.32
7.32
7.28
7.26 CDCl3
4.94
4.93
4.91
2.65
2.60
2.54
2.48
2.42
2.39
2.33
2.30
0.99
1.00
1.03
0.81
1.02
0.95
1.09
2.15
f1 (ppm)

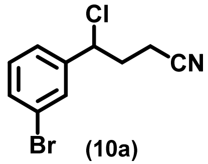

(10a)

^{13}C-NMR (125 MHz, $CDCl_3$)

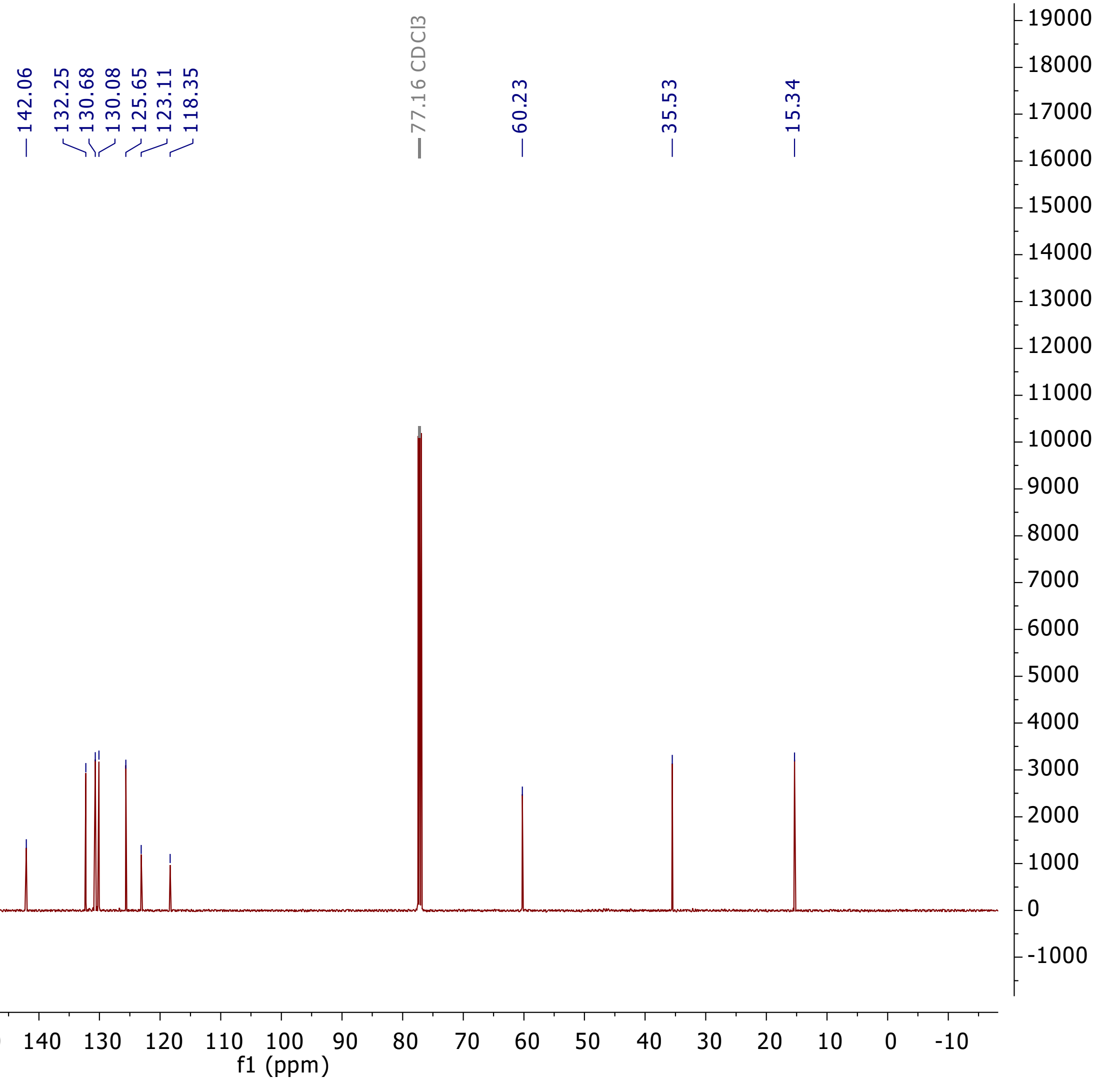

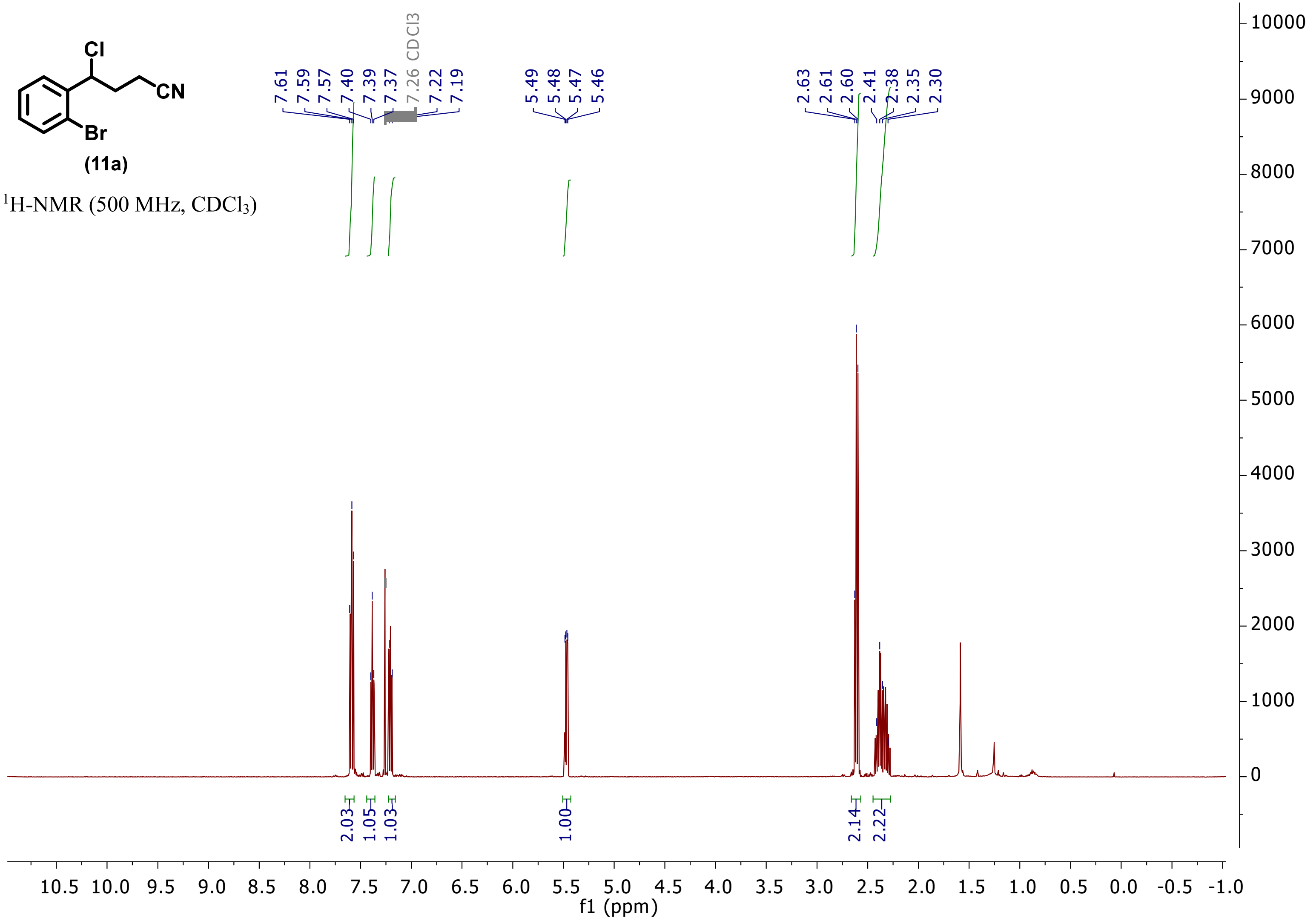

^{1}H-NMR (500 MHz, $CDCl_3$)

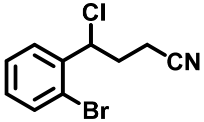

(11a)

^{13}C-NMR (125 MHz, $CDCl_3$)

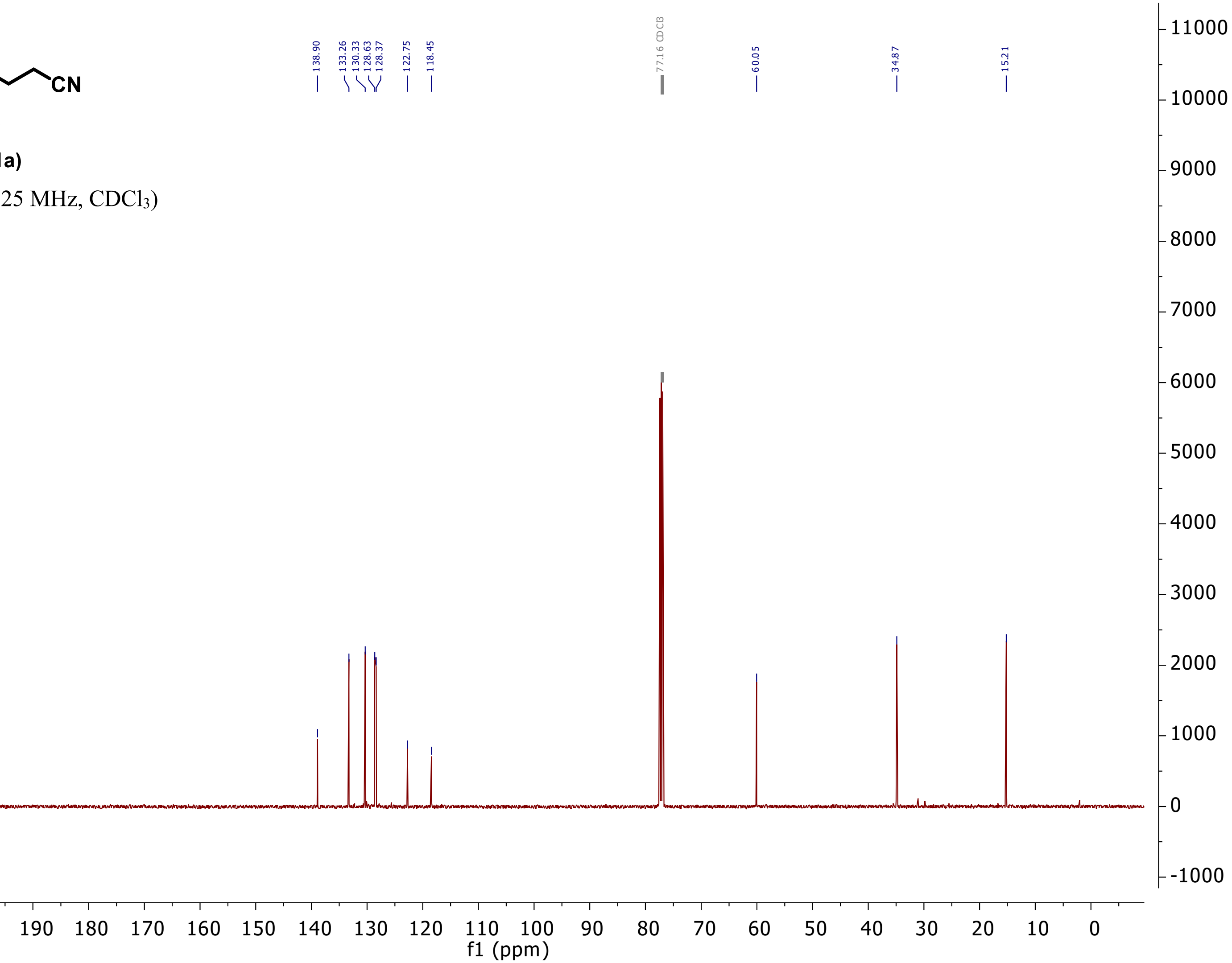

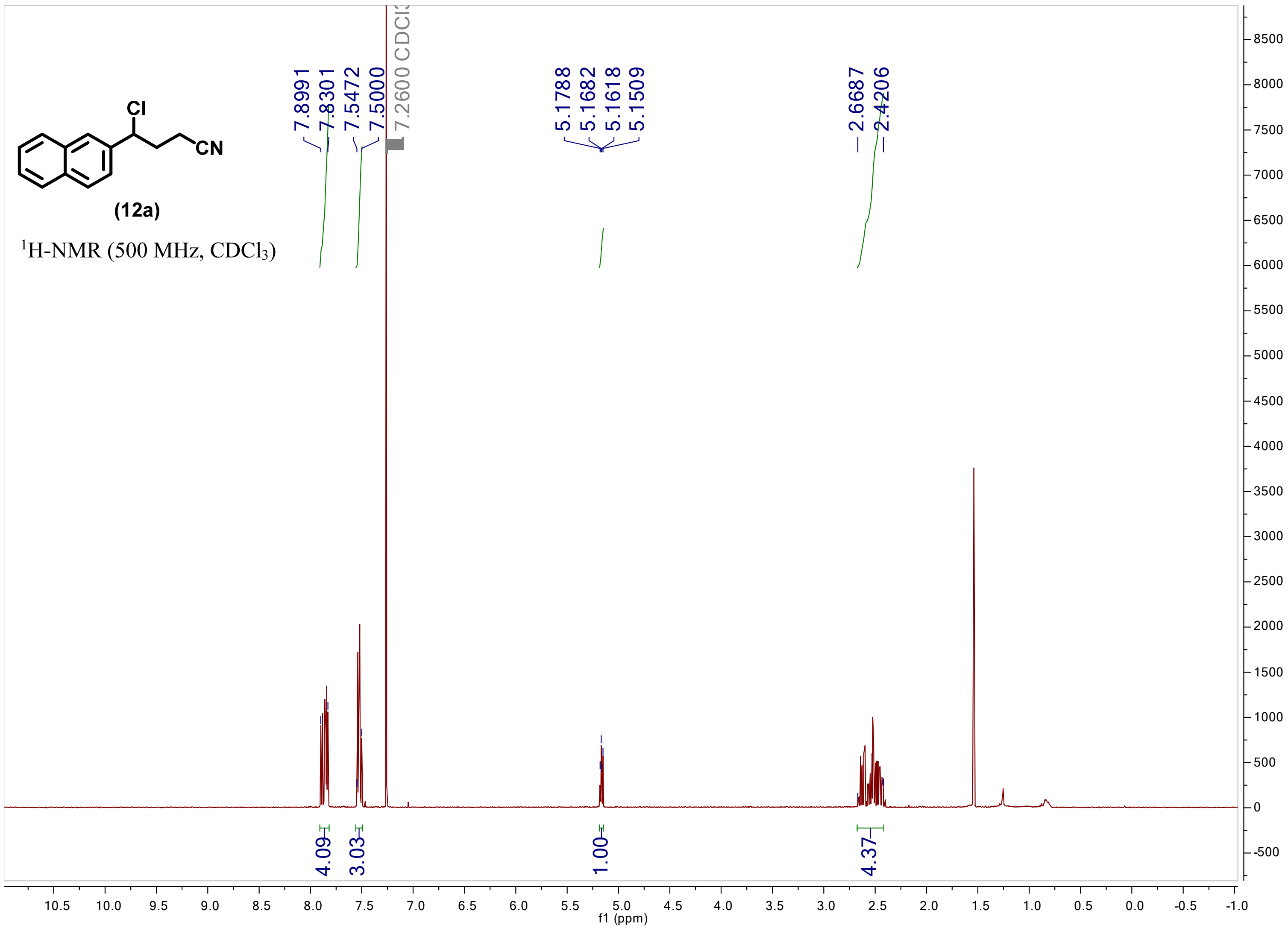

Cl
CN
(12a)
1H-NMR (500 MHz, CDCl3)
7.8991
7.8301
7.5472
7.5000
7.2600 CDCl3
5.1788
5.1682
5.1618
5.1509
2.6687
2.4206
4.09
3.03
1.00
4.37
f1 (ppm)

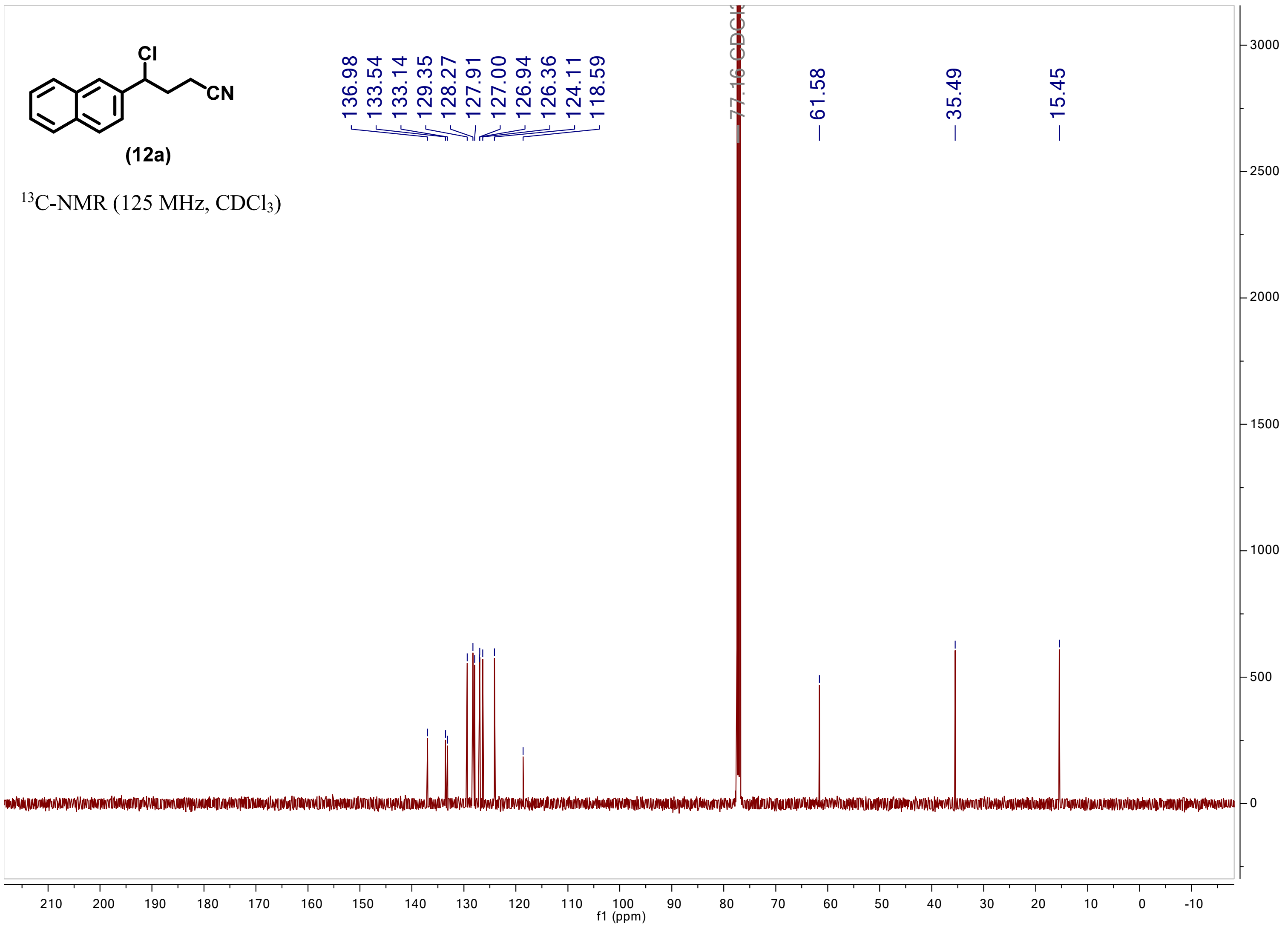

Cl
CN
(12a)
^{13}C-NMR (125 MHz, CDCl$_3$)
136.98
133.54
133.14
129.35
128.27
127.91
127.00
126.94
126.36
124.11
118.59
77.16 CDCl3
61.58
35.49
15.45
f1 (ppm)

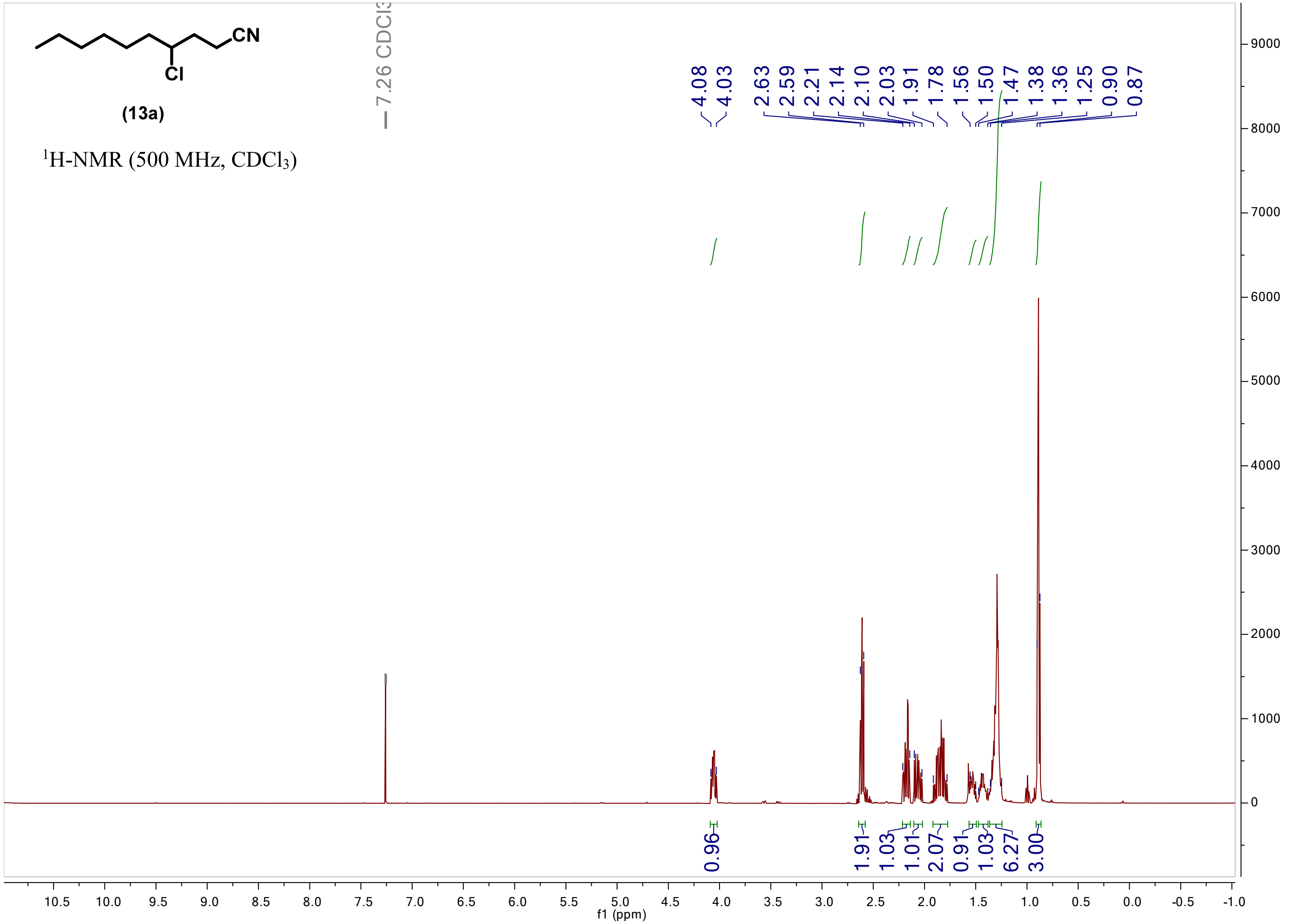
CN
Cl
(13a)
¹H-NMR (500 MHz, CDCl₃)
7.26 CDCl3
4.08
4.03
2.63
2.59
2.21
2.14
2.10
2.03
1.91
1.78
1.56
1.50
1.47
1.38
1.36
1.25
0.90
0.87
0.96
1.91
1.03
1.01
2.07
0.91
1.03
6.27
3.00
f1 (ppm)

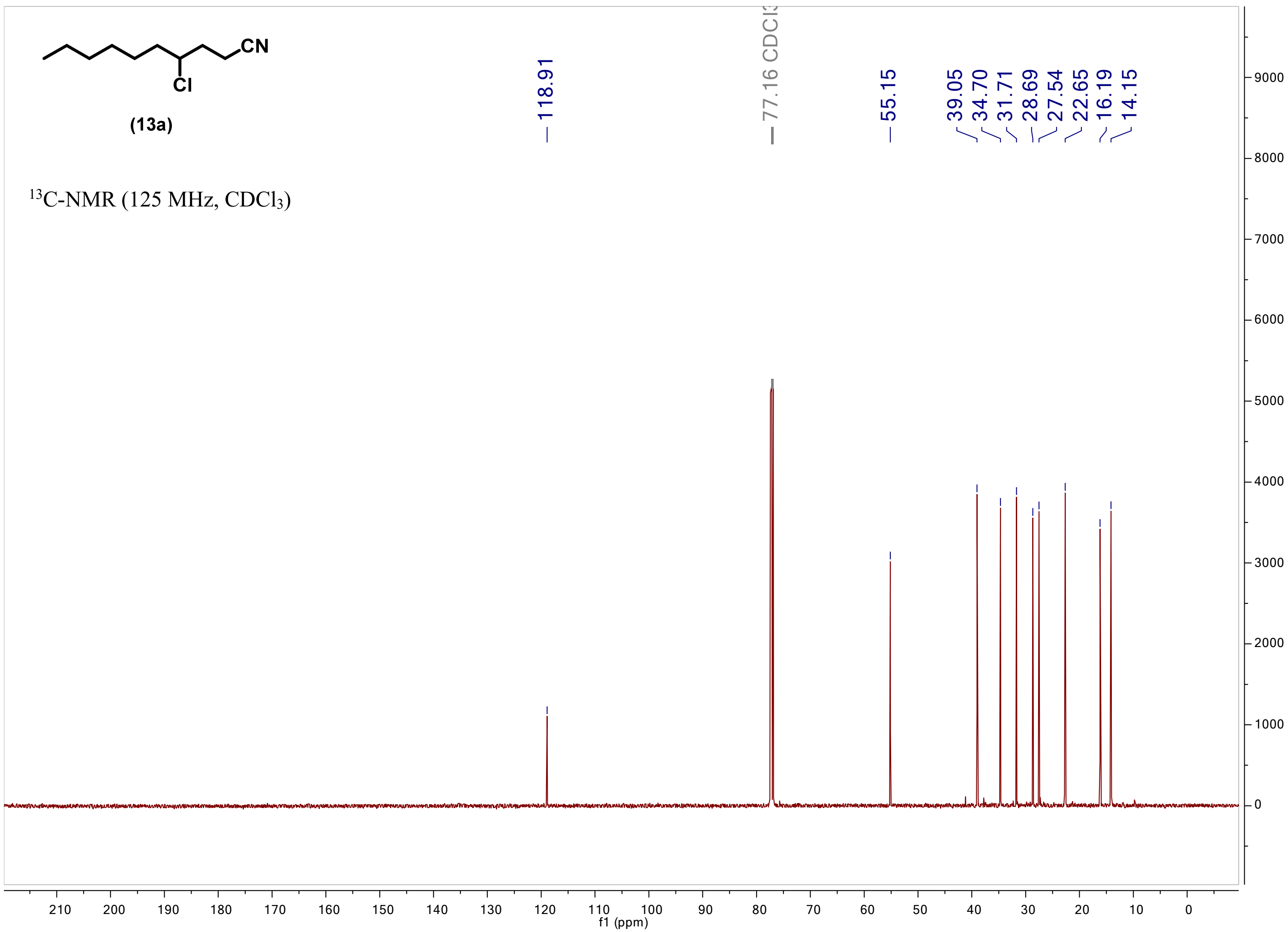
CN
Cl
(13a)
^{13}C-NMR (125 MHz, CDCl$_3$)
118.91
77.16 CDCl3
55.15
39.05
34.70
31.71
28.69
27.54
22.65
16.19
14.15
9000
8000
7000
6000
5000
4000
3000
2000
1000
0
210
200
190
180
170
160
150
140
130
120
110
100
90
80
70
60
50
40
30
20
10
0
f1 (ppm)

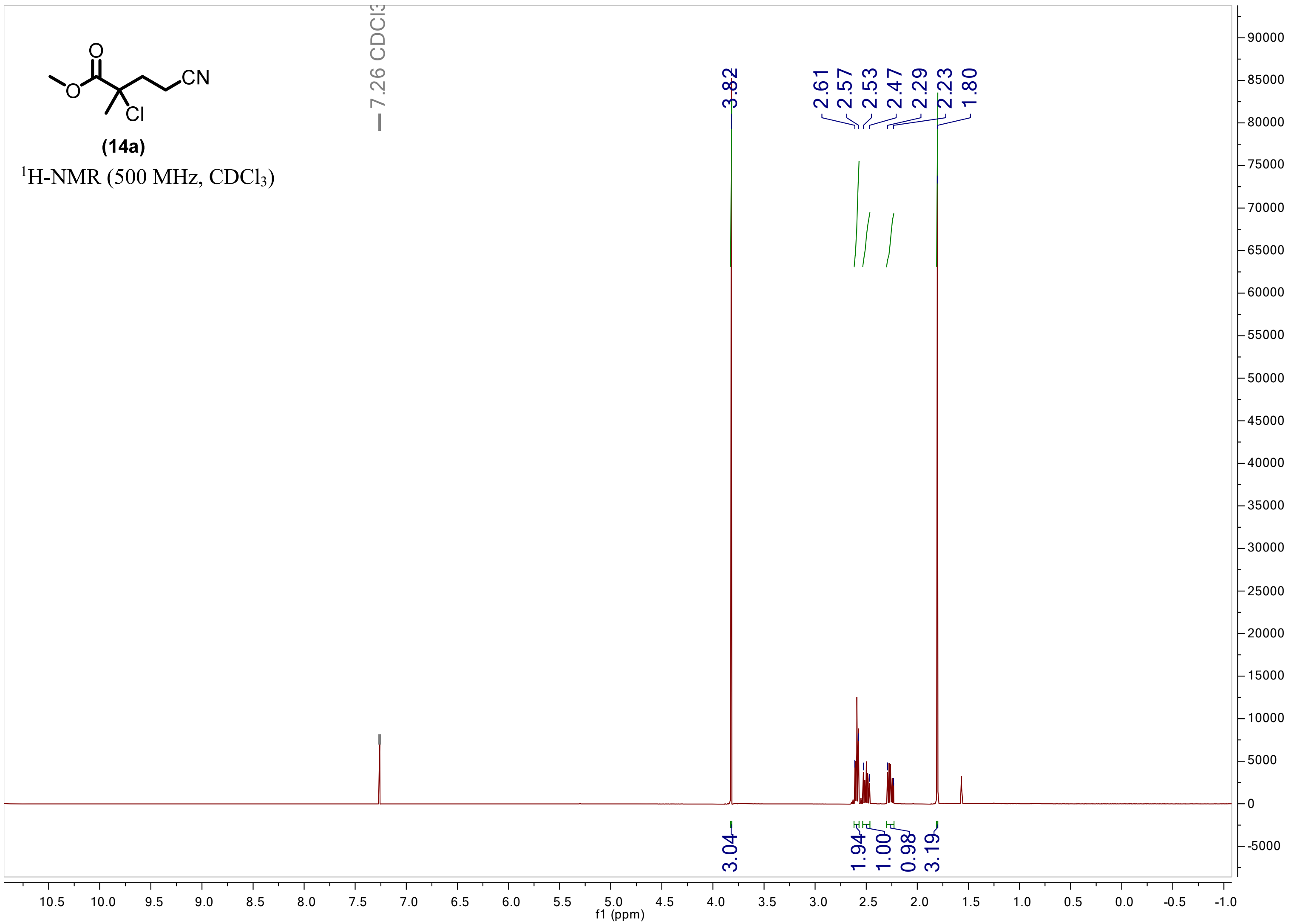

^{1}H-NMR (500 MHz, $CDCl_3$)

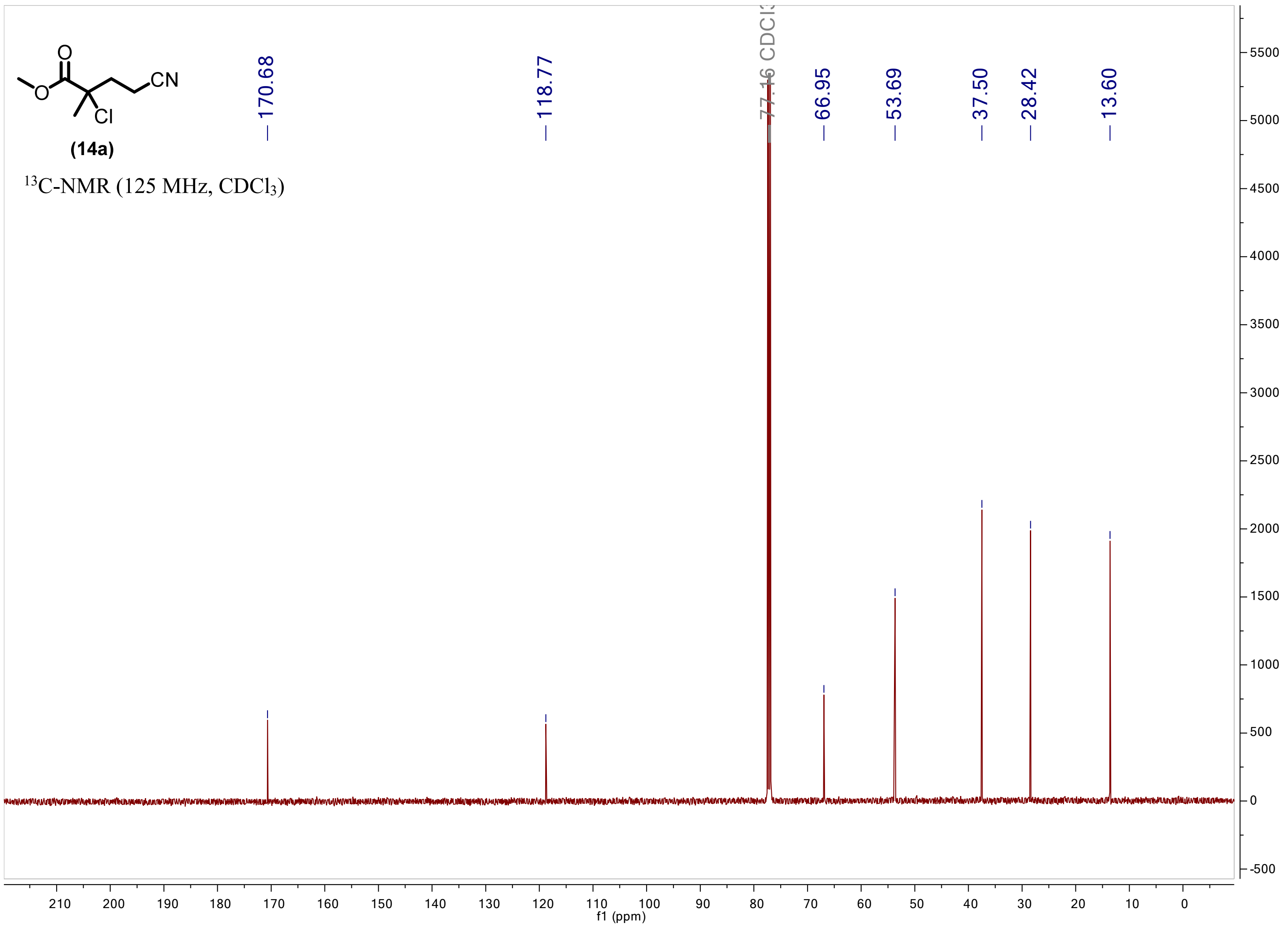
(14a)
^{13}C-NMR (125 MHz, CDCl$_3$)
170.68
118.77
77.16 CDCl3
66.95
53.69
37.50
28.42
13.60
f1 (ppm)

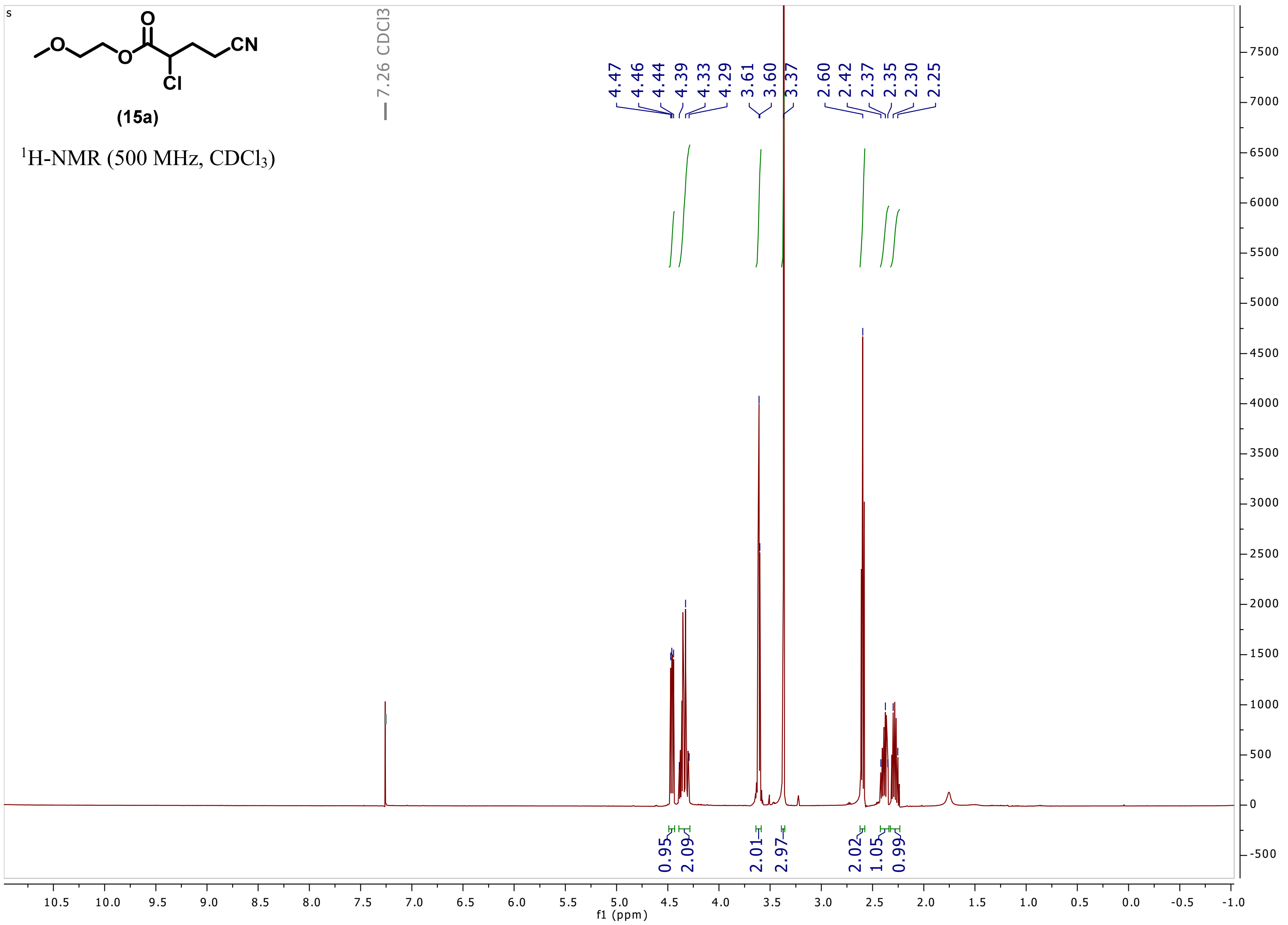

(15a)
^{1}H-NMR (500 MHz, $CDCl_3$)
7.26 CDCl3
4.47 4.46 4.44 4.39 4.33 4.29 3.61 3.60 3.37 2.60 2.42 2.37 2.35 2.30 2.25
0.95 2.09 2.01 2.97 2.02 1.05 0.99
f1 (ppm)

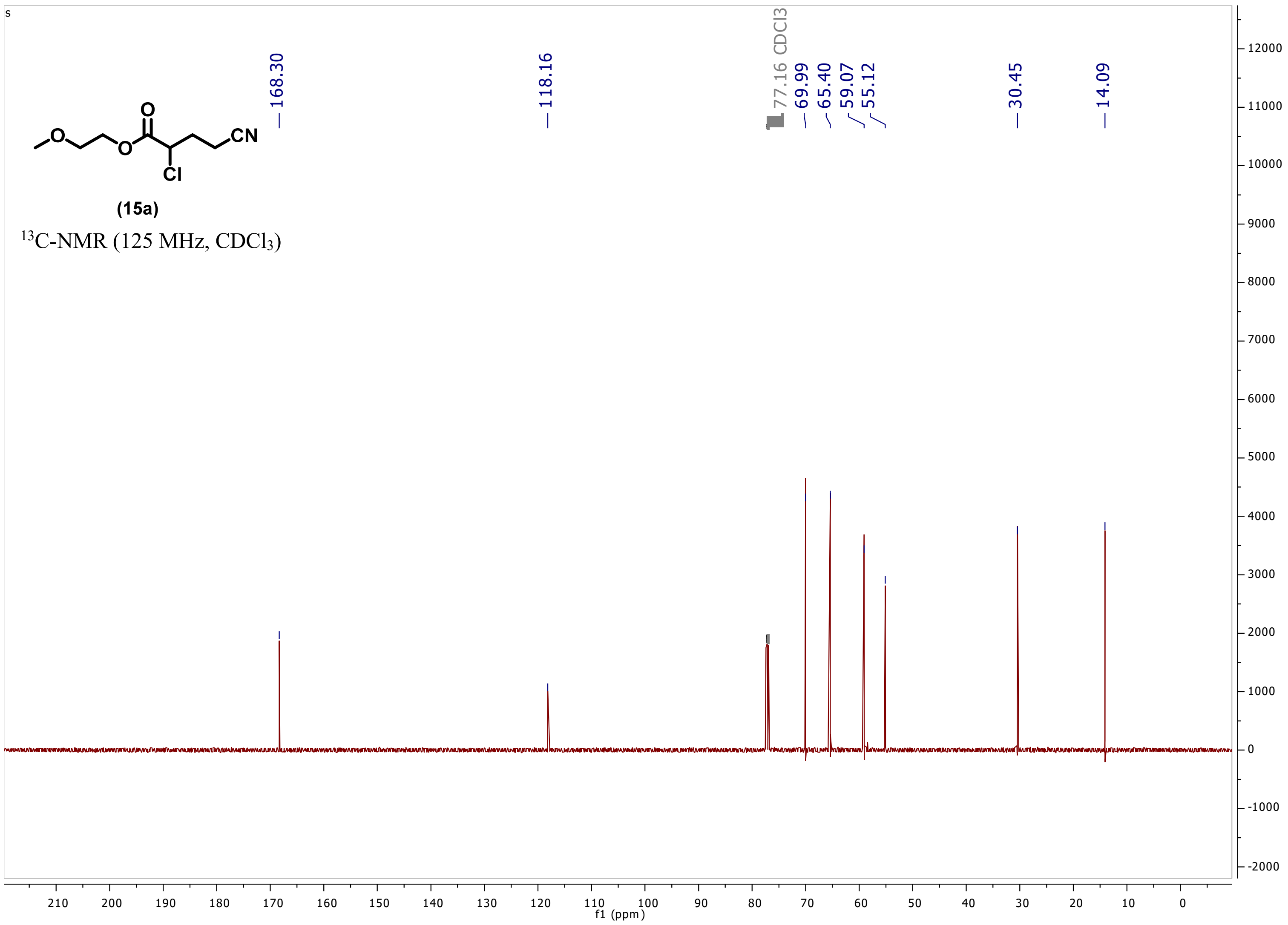

(15a)

^{13}C-NMR (125 MHz, $CDCl_3$)

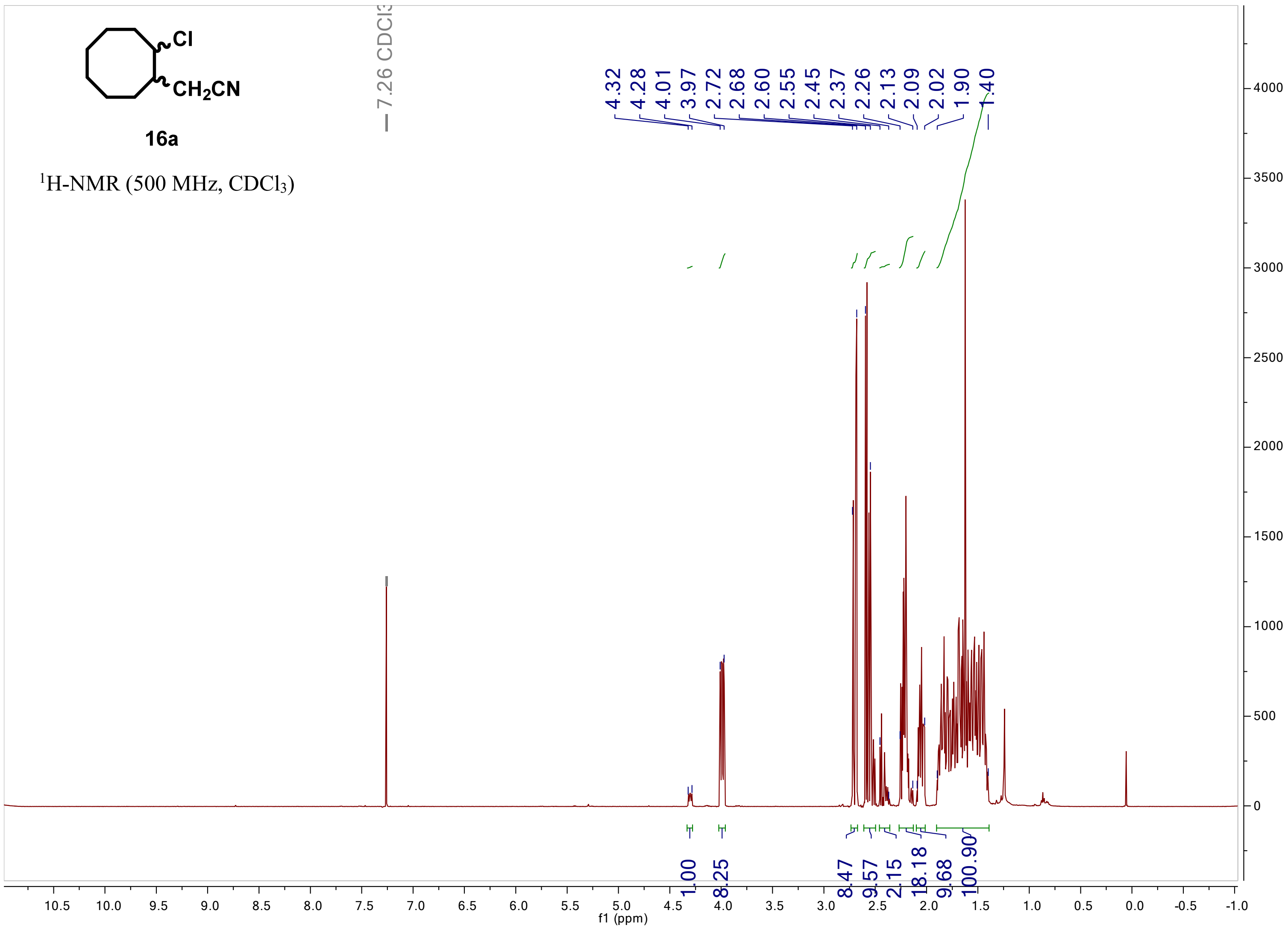

Cl
CH2CN
16a
1H-NMR (500 MHz, CDCl3)
7.26 CDCl3
4.32
4.28
4.01
3.97
2.72
2.68
2.60
2.55
2.45
2.37
2.26
2.13
2.09
2.02
1.90
1.40
1.00
8.25
8.47
9.57
2.15
18.18
9.68
100.90
f1 (ppm)

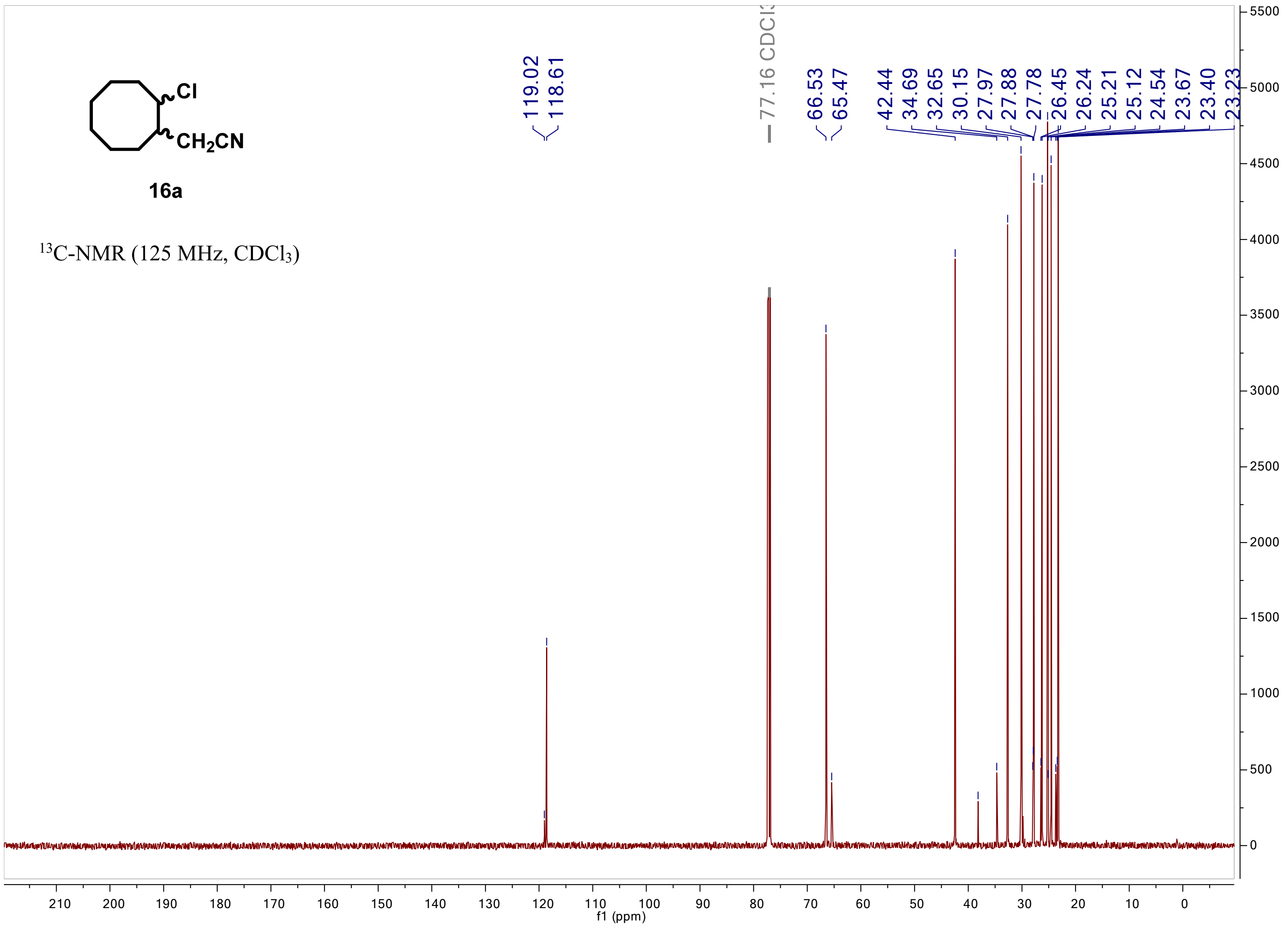
Cl
CH2CN
16a
13C-NMR (125 MHz, CDCl3)
119.02
118.61
77.16 CDCl3
66.53
65.47
42.44
34.69
32.65
30.15
27.97
27.88
27.78
26.45
26.24
25.21
25.12
24.54
23.67
23.40
23.23
f1 (ppm)

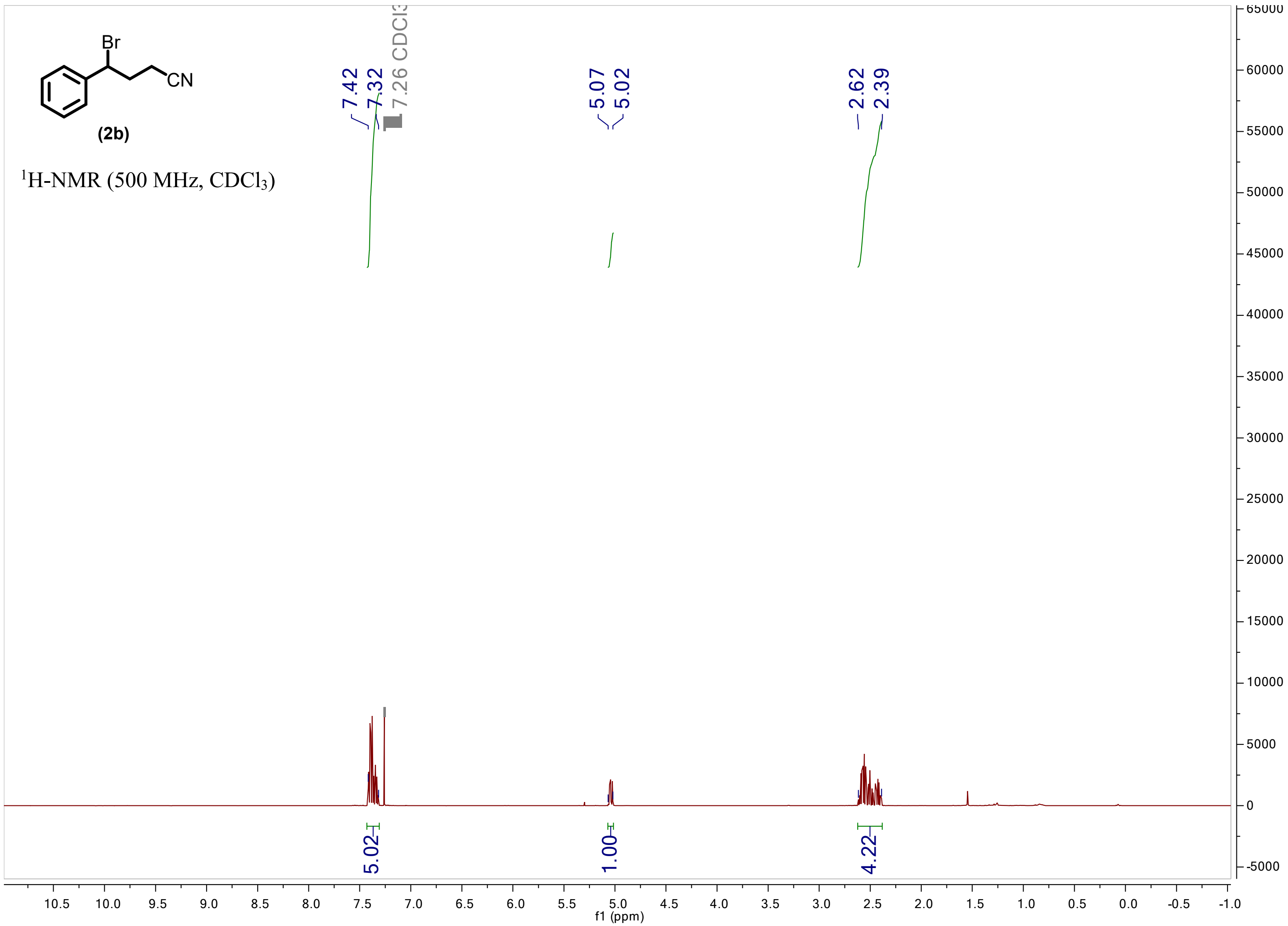
Br
CN
(2b)
¹H-NMR (500 MHz, CDCl₃)
7.42
7.32
7.26 CDCl3
5.07
5.02
2.62
2.39
5.02
1.00
4.22
f1 (ppm)

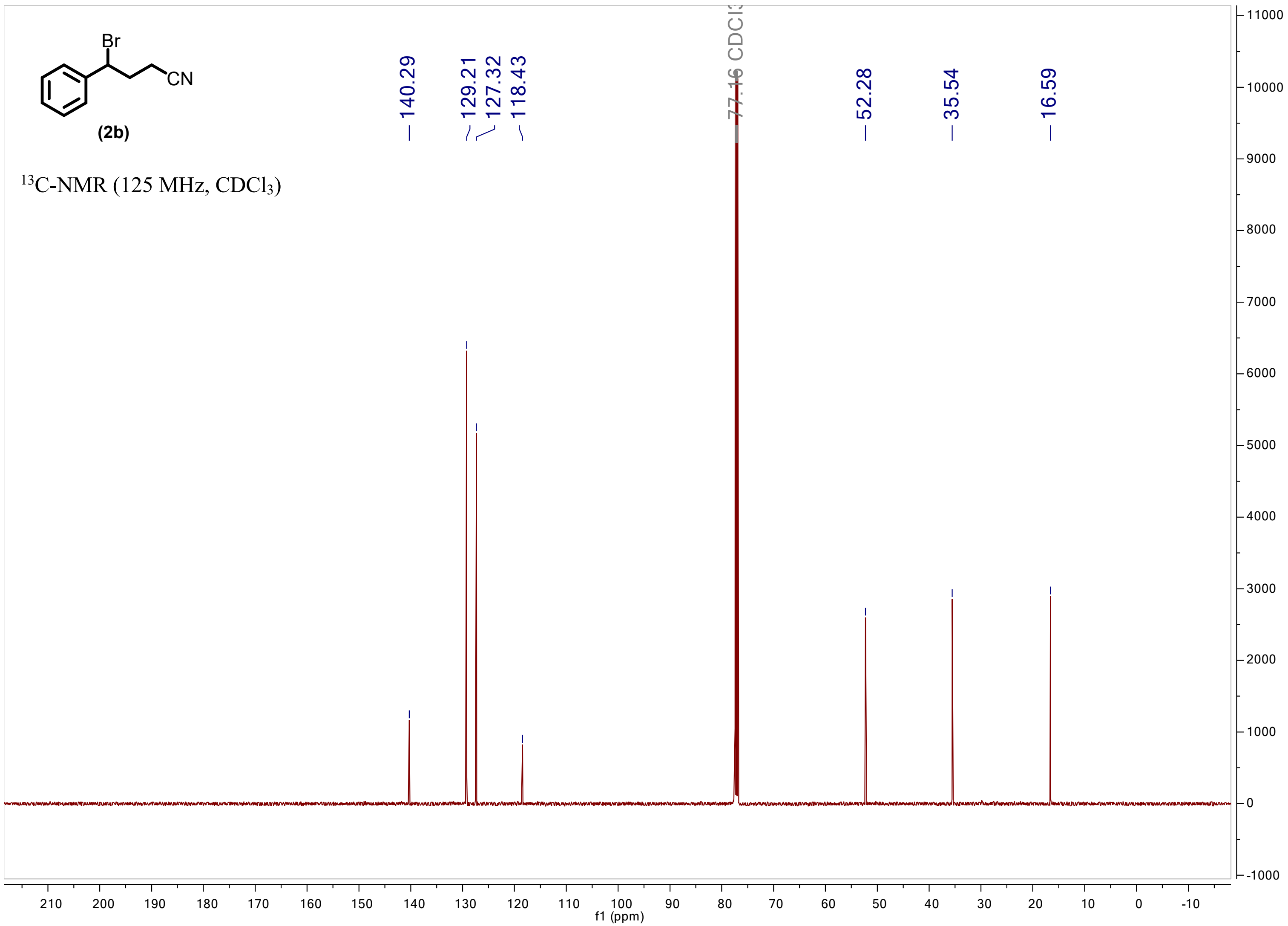

Br
CN
(2b)
^{13}C-NMR (125 MHz, $CDCl_3$)
140.29
129.21
127.32
118.43
77.16 CDCl3
52.28
35.54
16.59
11000
10000
9000
8000
7000
6000
5000
4000
3000
2000
1000
0
-1000
210
200
190
180
170
160
150
140
130
120
110
100
90
80
70
60
50
40
30
20
10
0
-10
f1 (ppm)

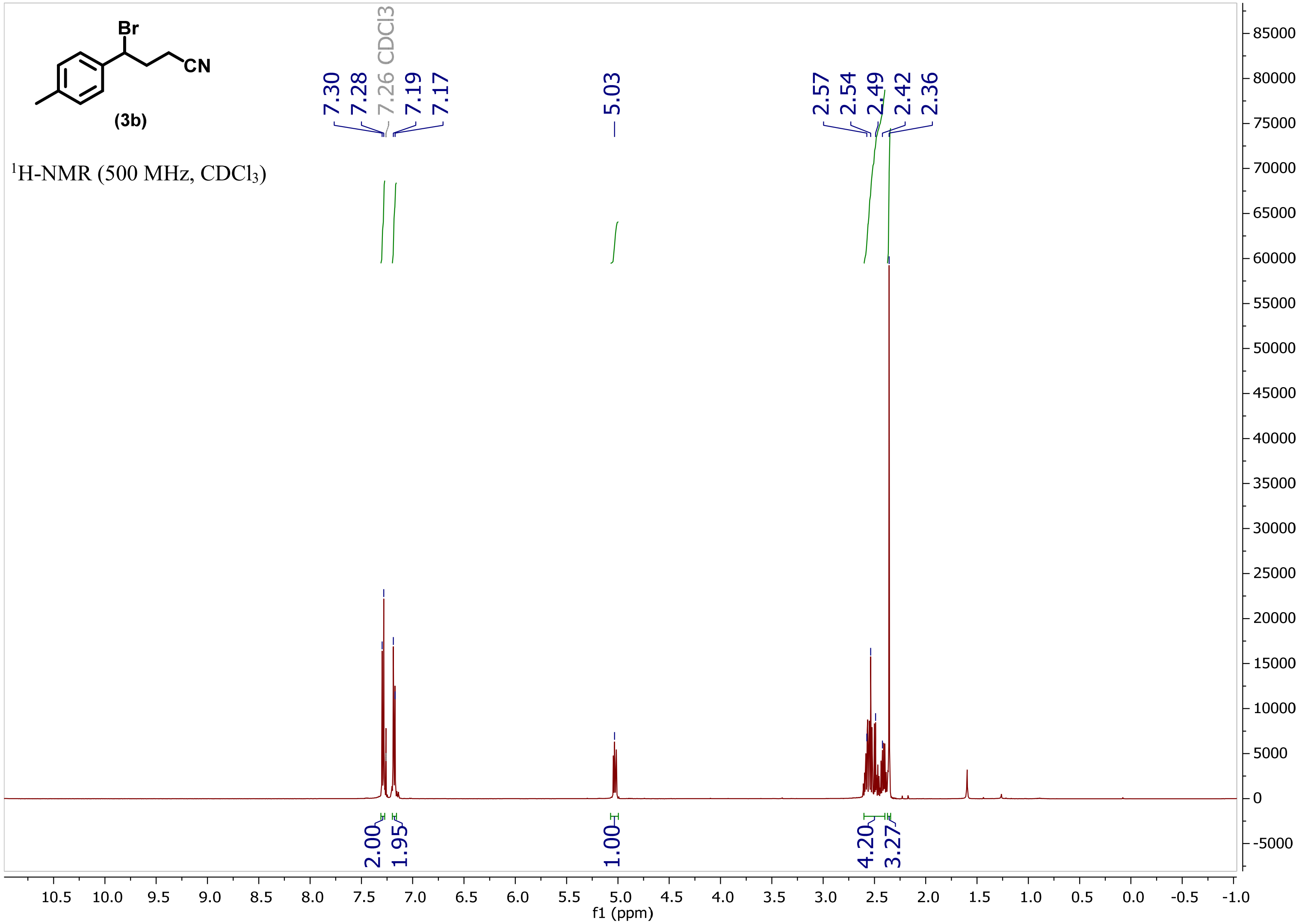
Br
CN
(3b)
^{1}H-NMR (500 MHz, $CDCl_3$)
7.30
7.28
7.26 CDCl3
7.19
7.17
5.03
2.57
2.54
2.49
2.42
2.36
2.00
1.95
1.00
4.20
3.27
f1 (ppm)

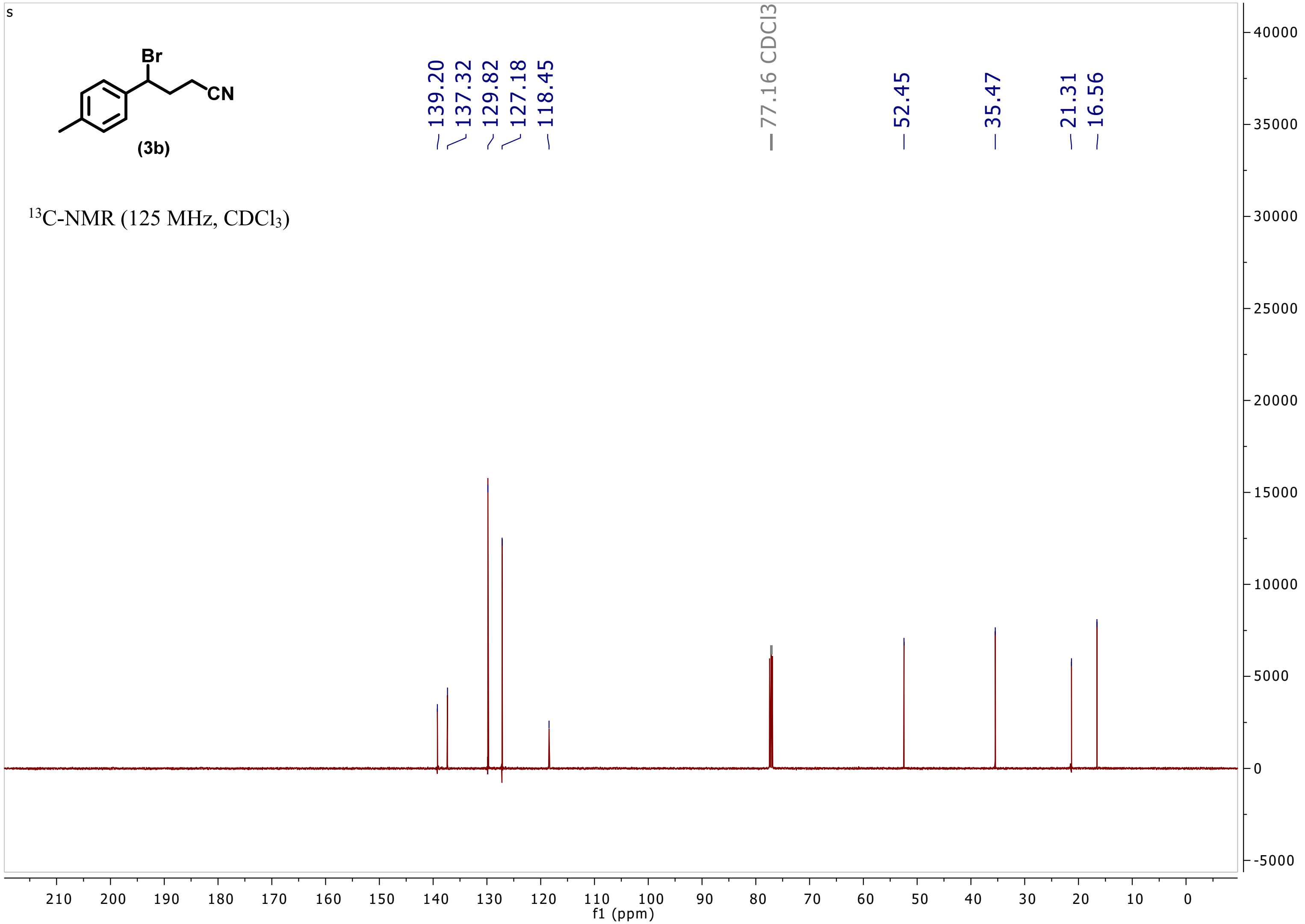

^{13}C-NMR (125 MHz, $CDCl_3$)

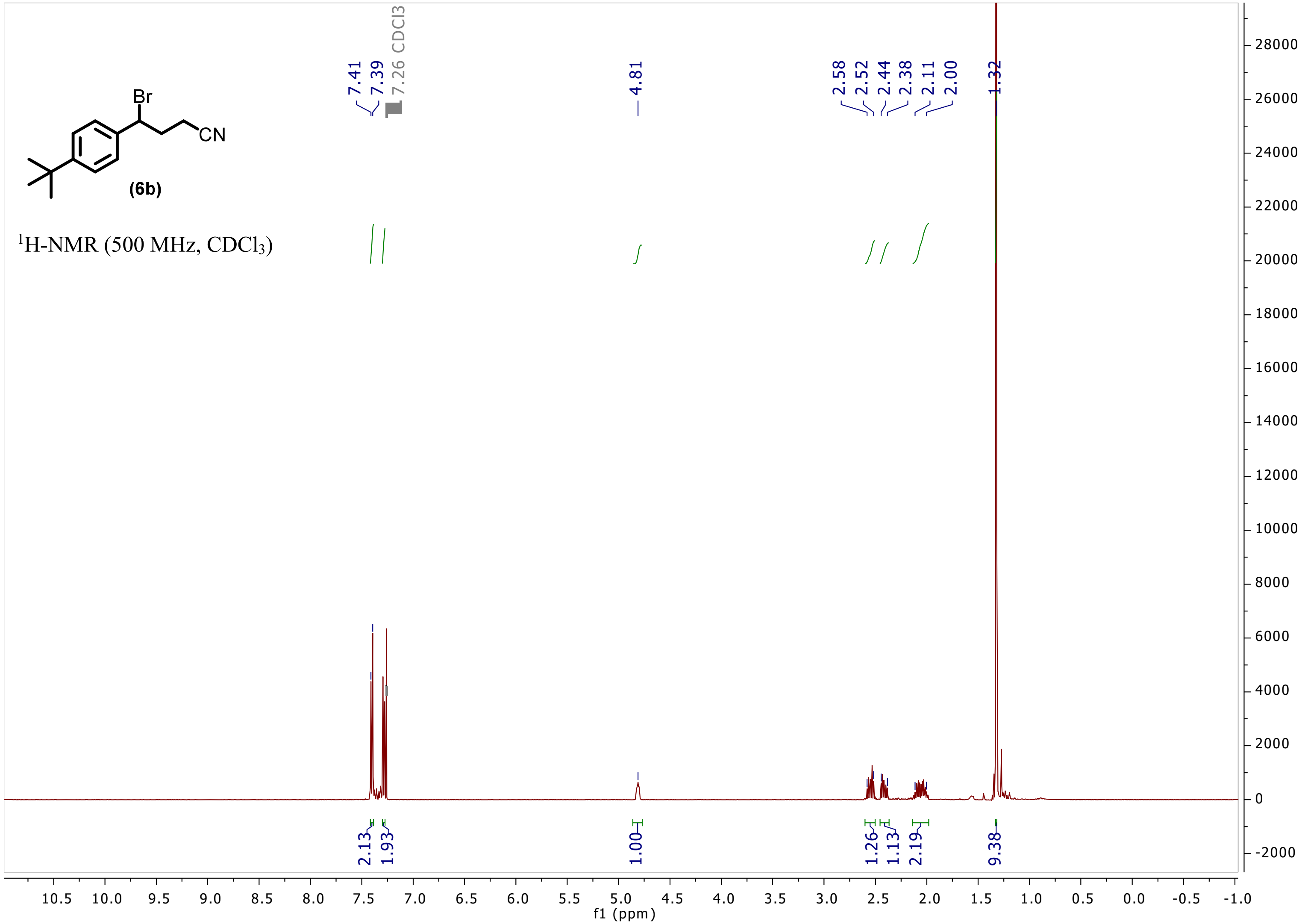

Br
CN
(6b)
^{1}H-NMR (500 MHz, CDCl$_3$)
7.41
7.39
7.26 CDCl3
4.81
2.58
2.52
2.44
2.38
2.11
2.00
1.32
2.13
1.93
1.00
1.26
1.13
2.19
9.38
f1 (ppm)

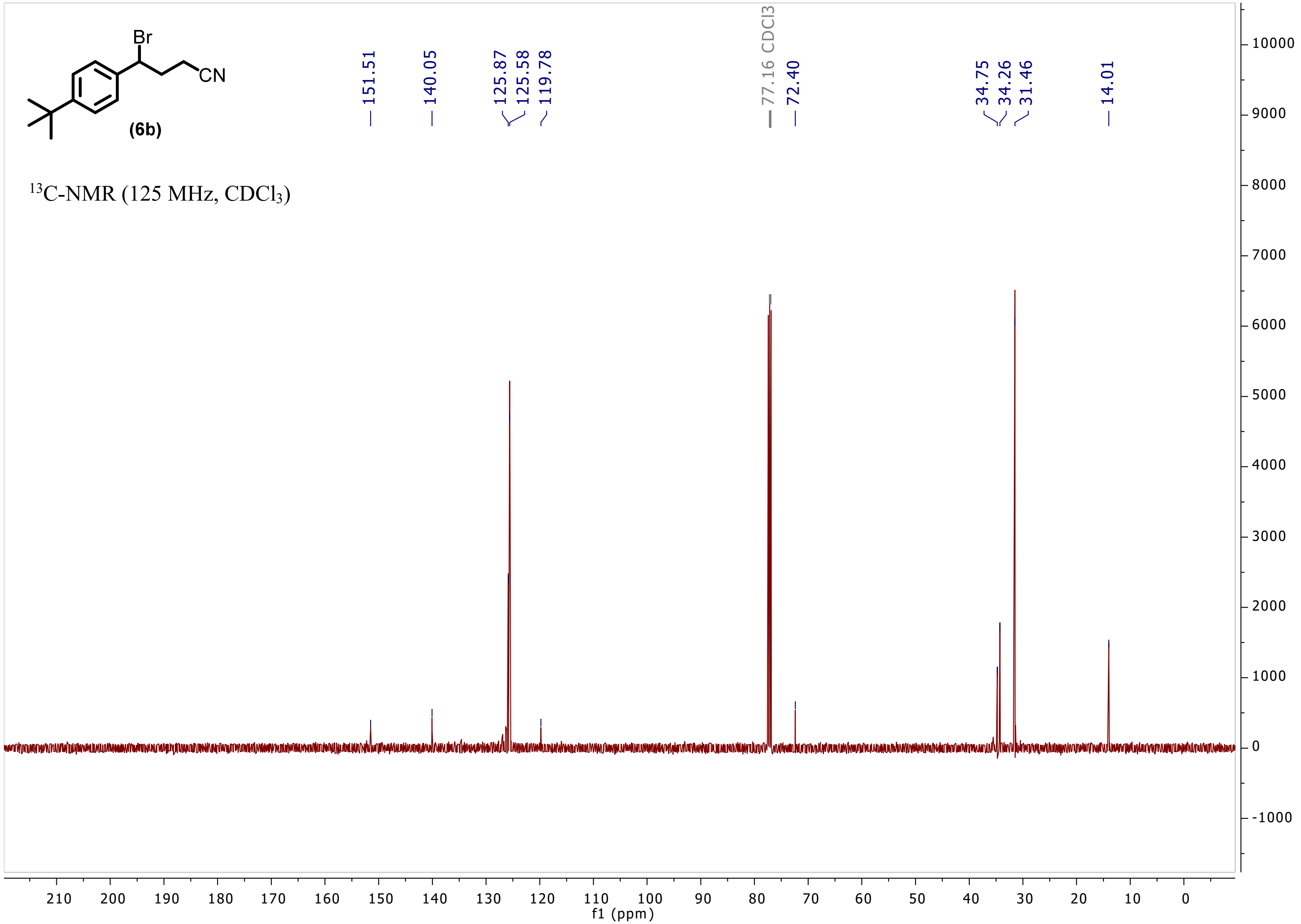
Br
CN
(6b)
13C-NMR (125 MHz, CDCl3)
151.51
140.05
125.87
125.58
119.78
77.16 CDCl3
72.40
34.75
34.26
31.46
14.01
10000
9000
8000
7000
6000
5000
4000
3000
2000
1000
0
-1000
210 200 190 180 170 160 150 140 130 120 110 100 90 80 70 60 50 40 30 20 10 0
f1 (ppm)

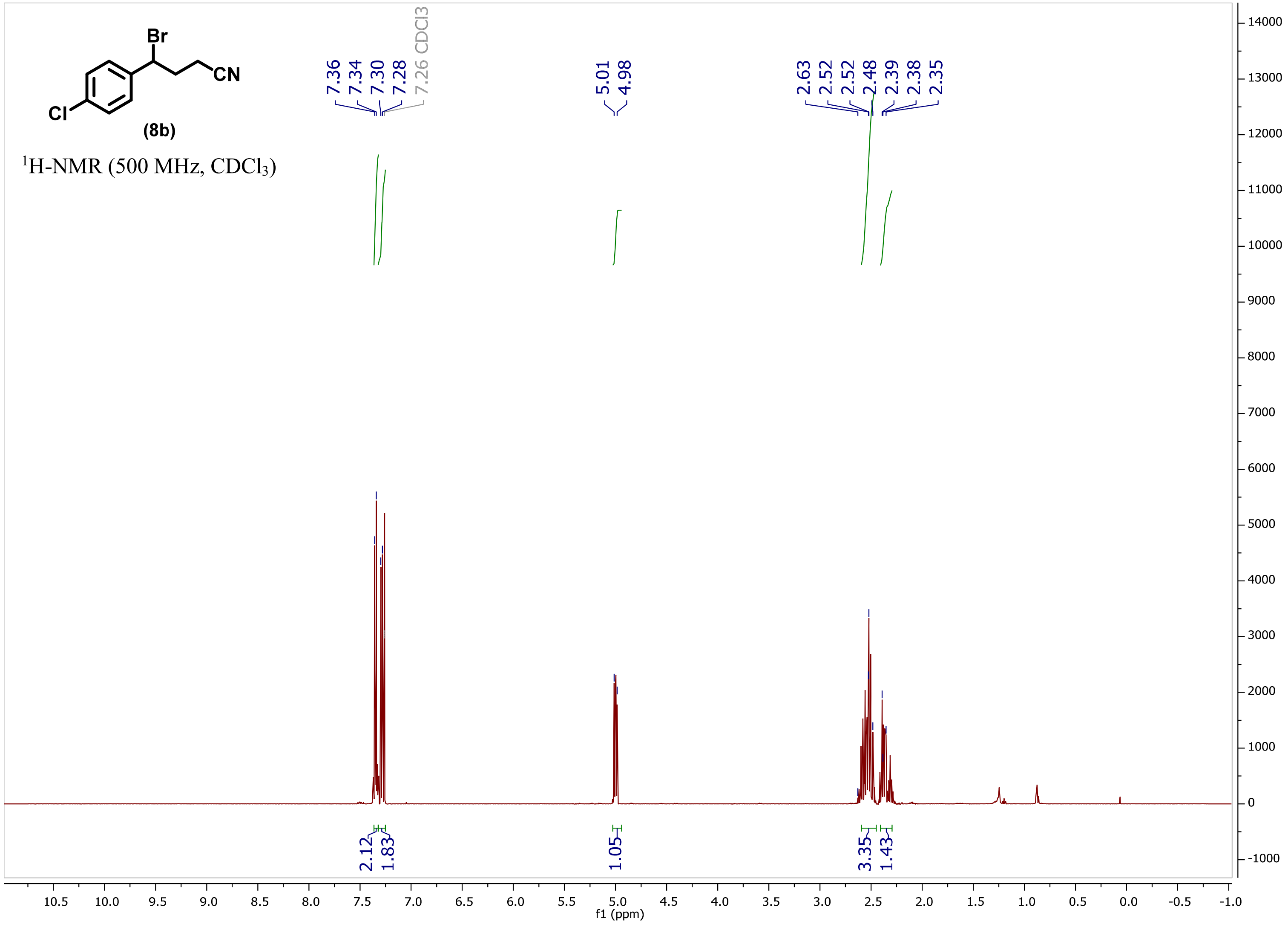
Br
CN
Cl
(8b)
^{1}H-NMR (500 MHz, CDCl$_3$)
7.36
7.34
7.30
7.28
7.26 CDCl3
5.01
4.98
2.63
2.52
2.52
2.48
2.39
2.38
2.35
2.12
1.83
1.05
3.35
1.43
f1 (ppm)

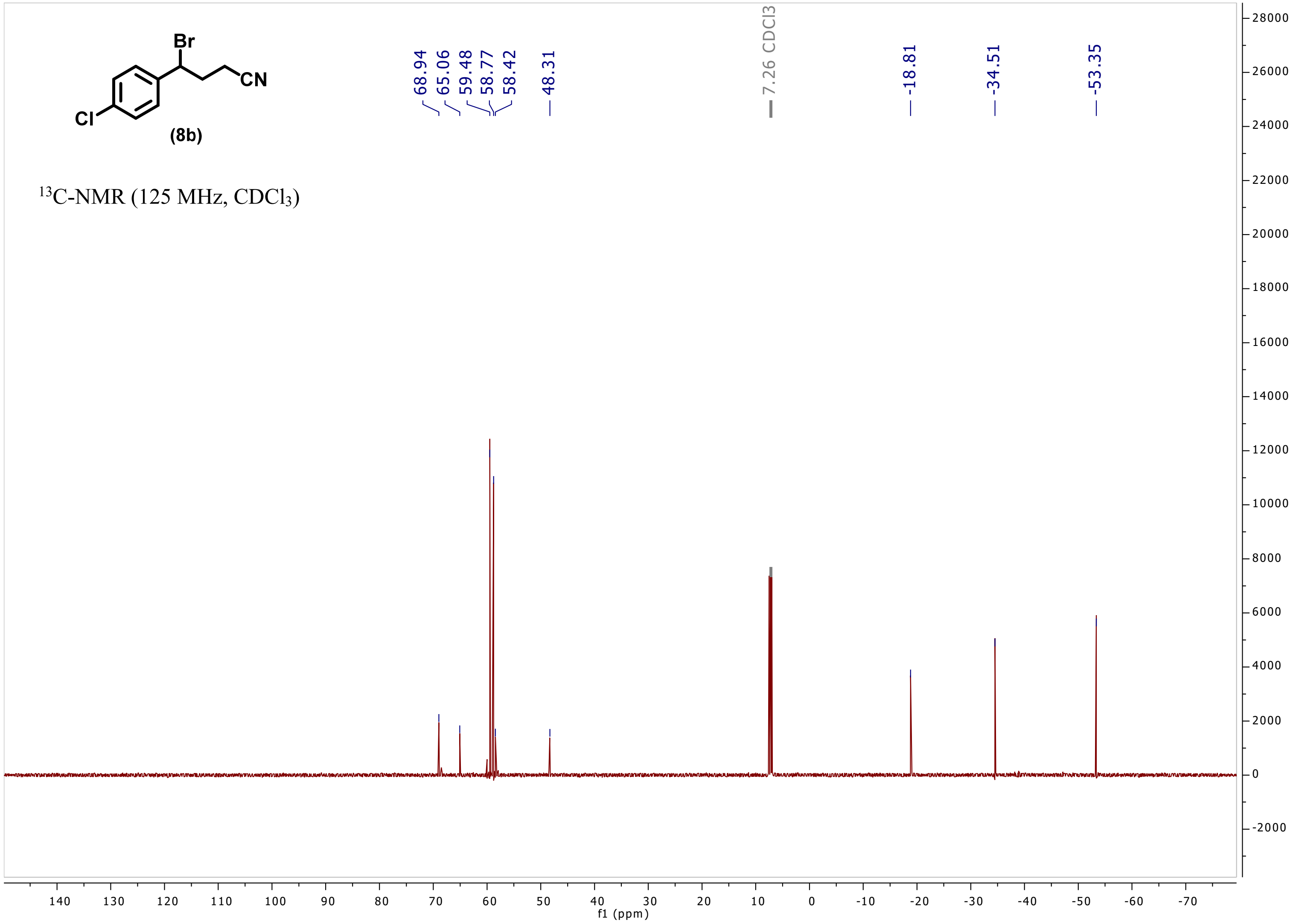

(8b)

^{13}C-NMR (125 MHz, $CDCl_3$)

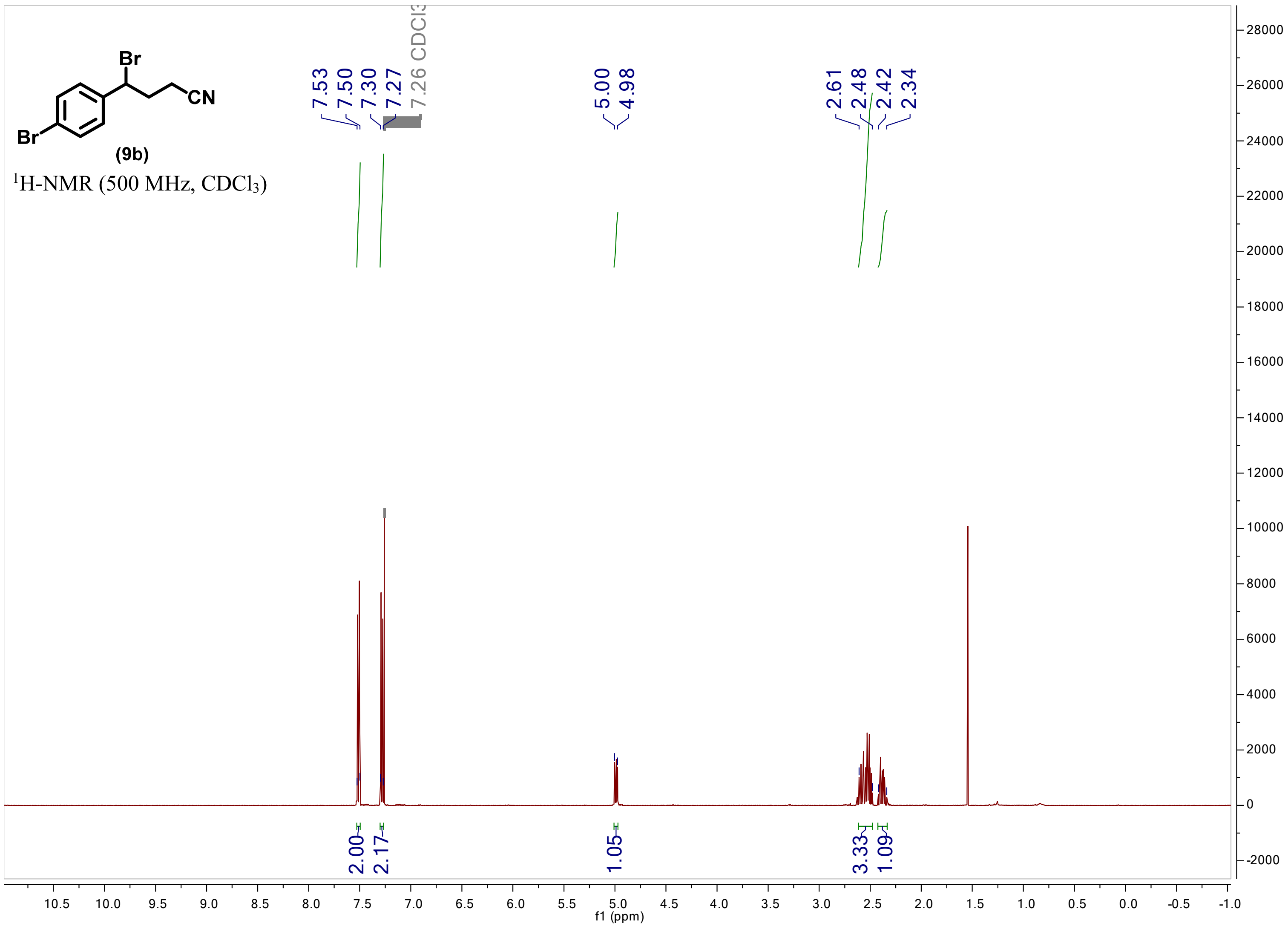
(9b)
1H-NMR (500 MHz, CDCl3)
7.53
7.50
7.30
7.27
7.26 CDCl3
5.00
4.98
2.61
2.48
2.42
2.34
2.00
2.17
1.05
3.33
1.09
f1 (ppm)

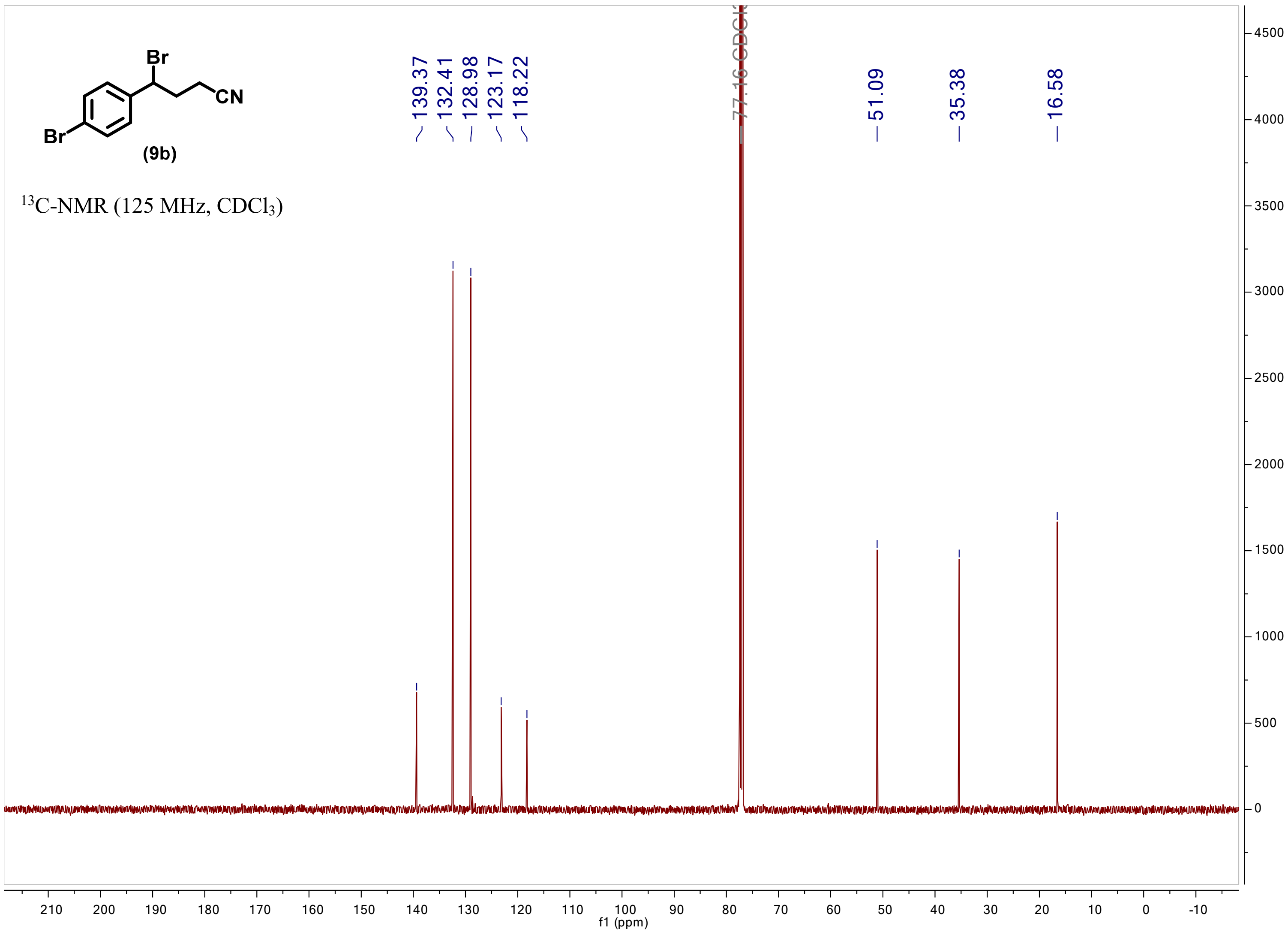

^{13}C-NMR (125 MHz, $CDCl_3$)

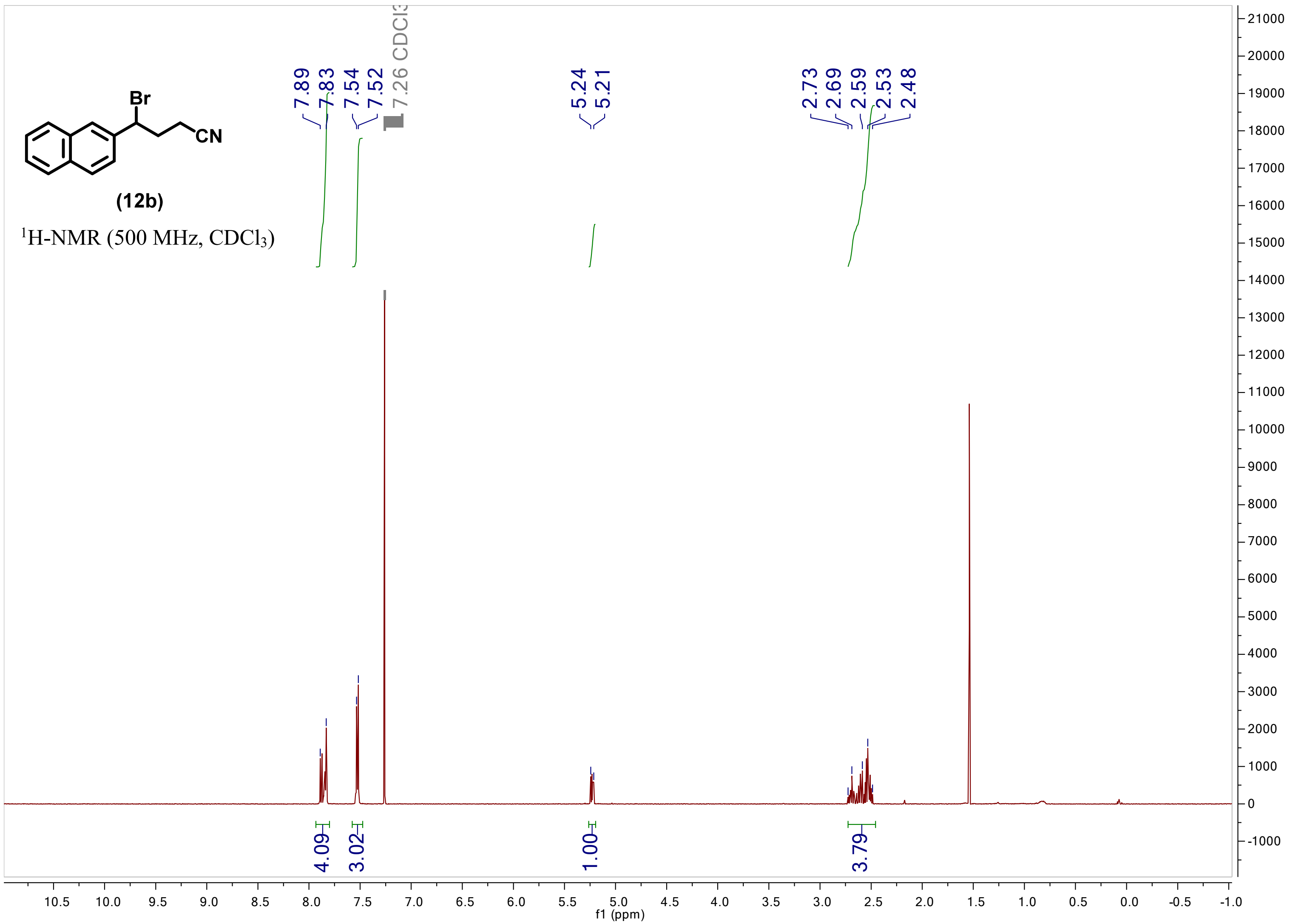
Br
CN
(12b)
¹H-NMR (500 MHz, CDCl₃)
7.89
7.83
7.54
7.52
7.26 CDCl3
5.24
5.21
2.73
2.69
2.59
2.53
2.48
4.09
3.02
1.00
3.79
f1 (ppm)

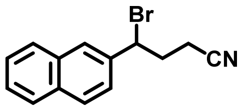

(12b)

^{13}C-NMR (125 MHz, $CDCl_3$)

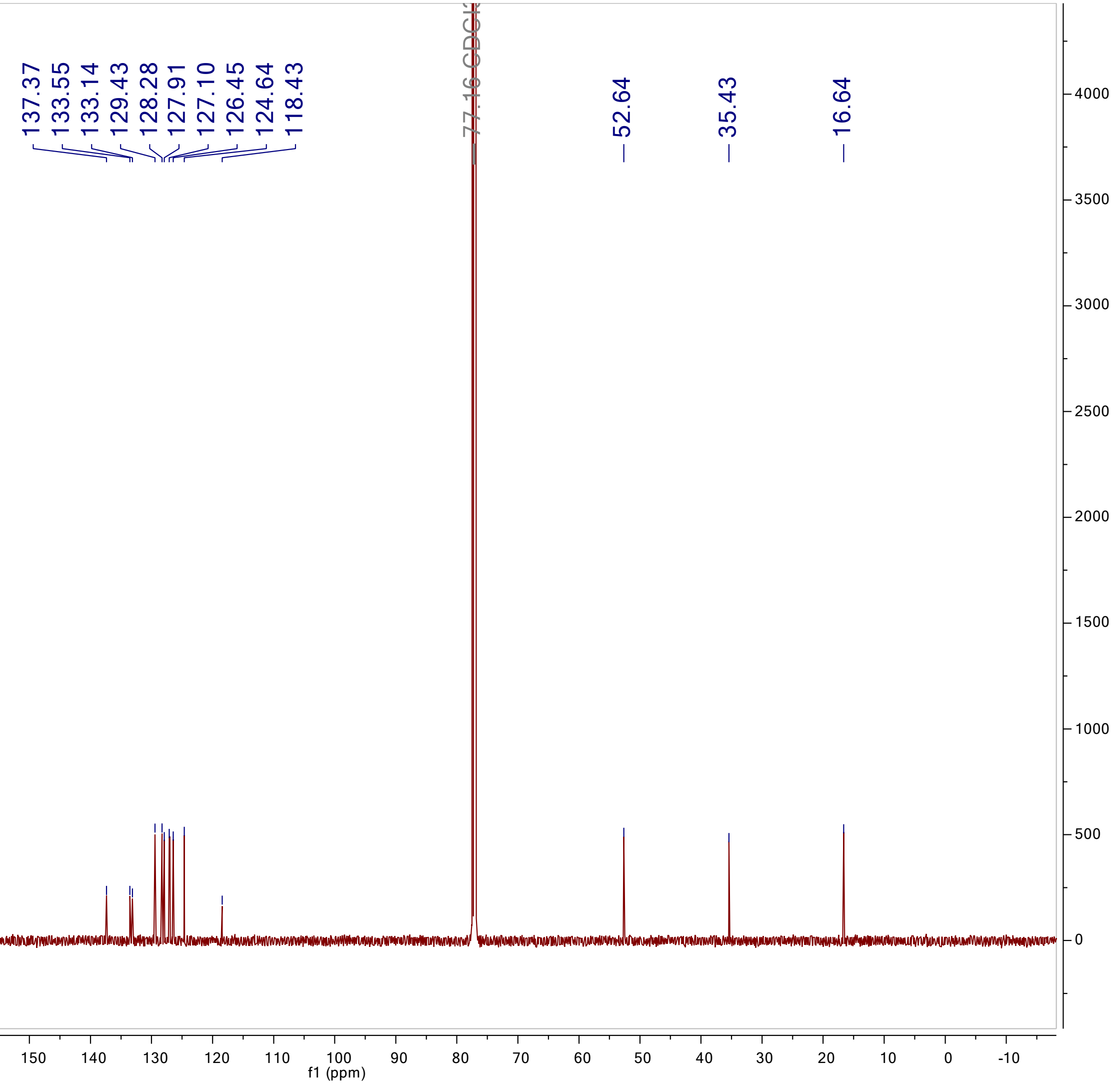

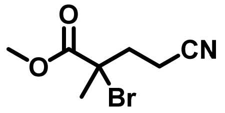

(14b)

^{1}H-NMR (500 MHz, $CDCl_3$)

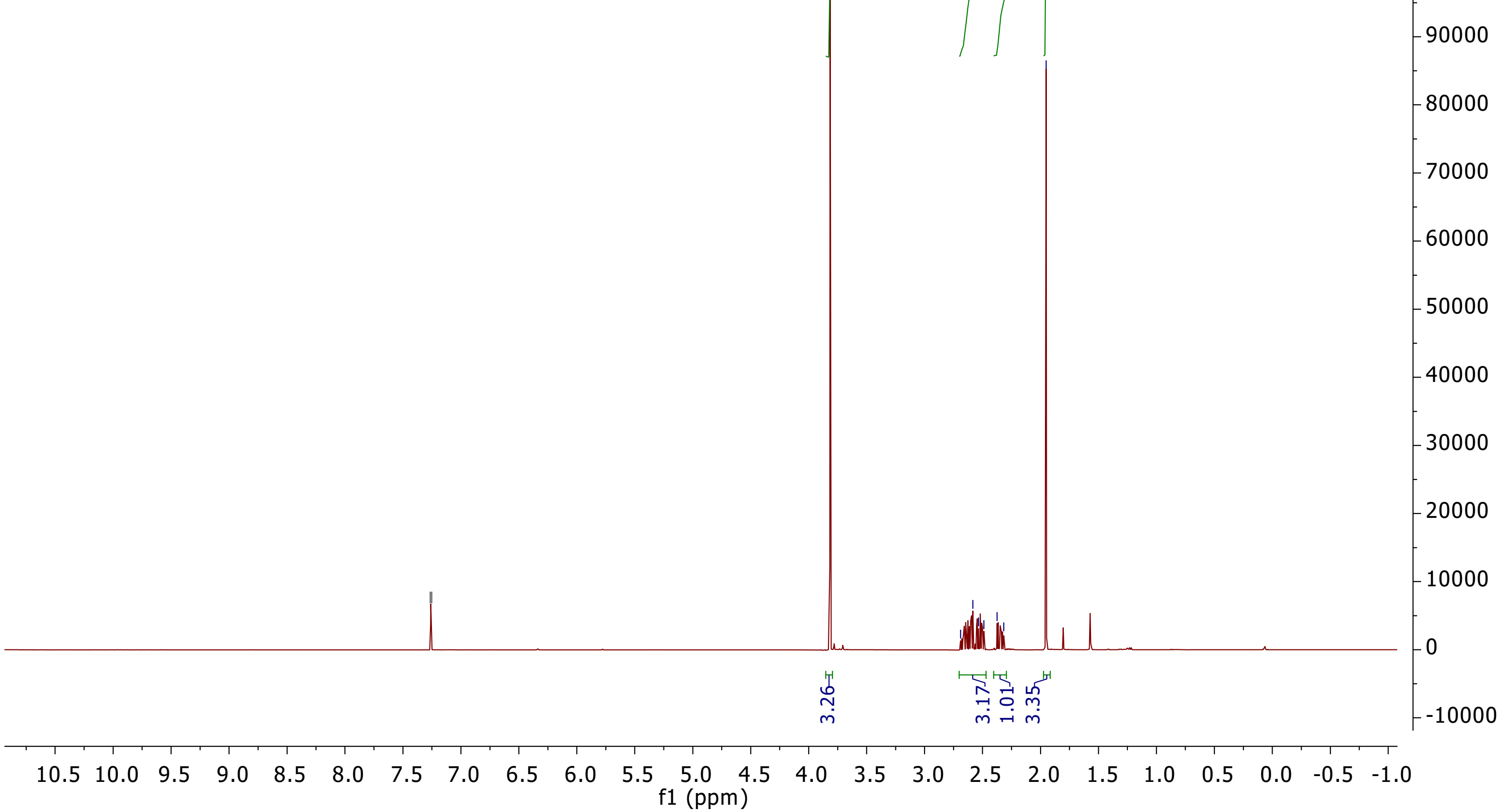

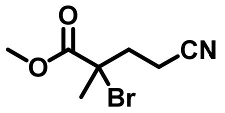

(14b)

^{13}C-NMR (125 MHz, $CDCl_3$)

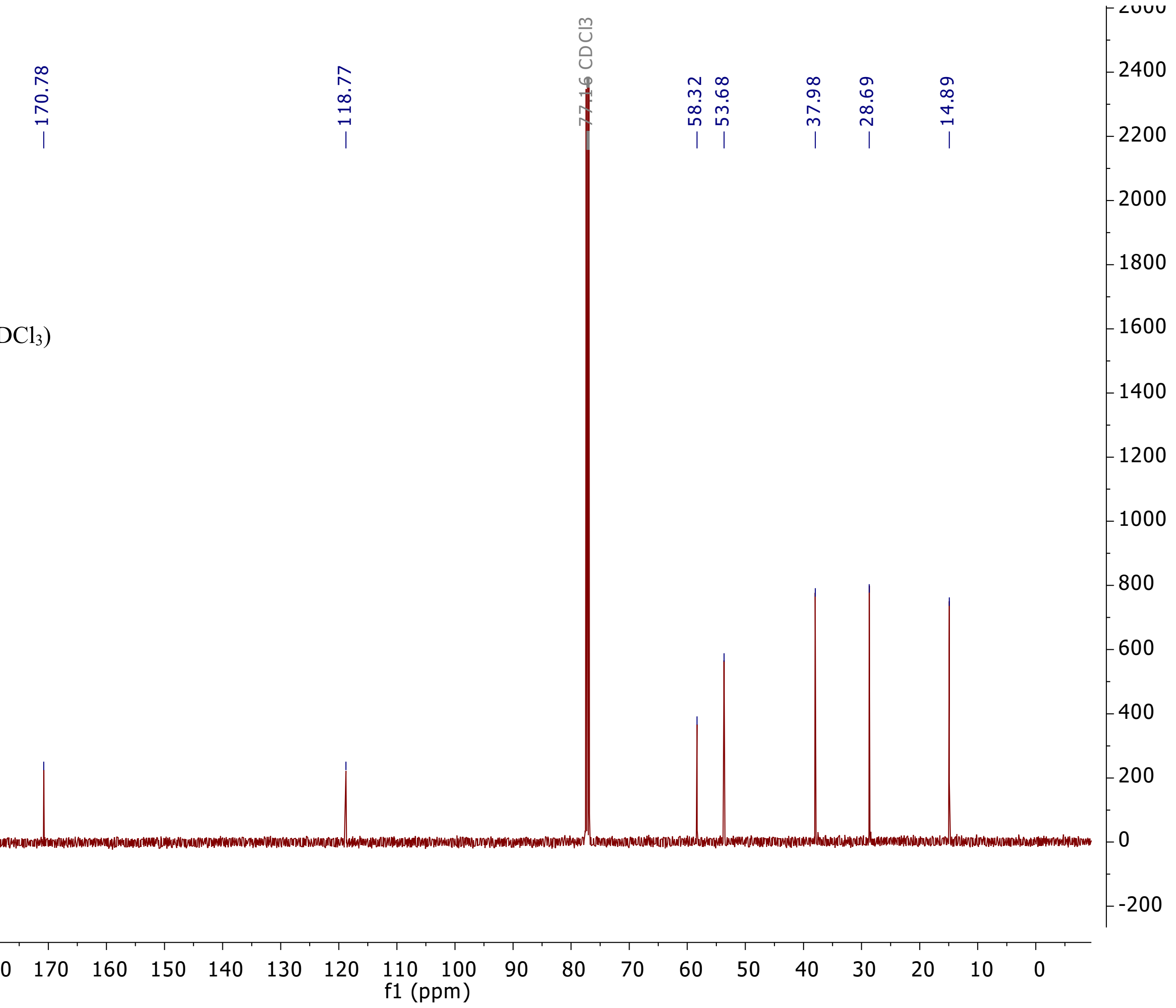

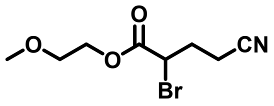

(15b)

^{1}H-NMR (500 MHz, $CDCl_3$)

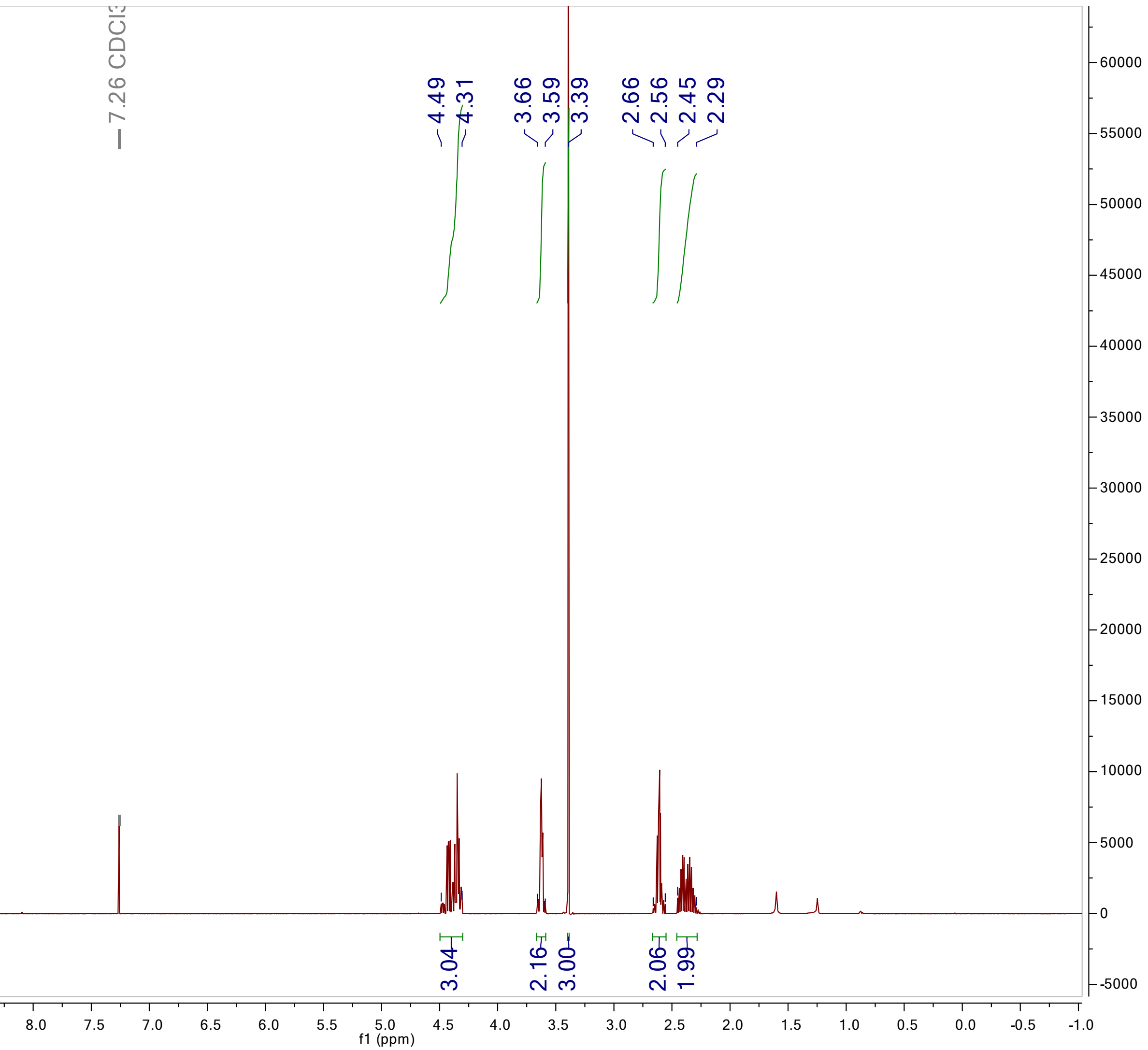

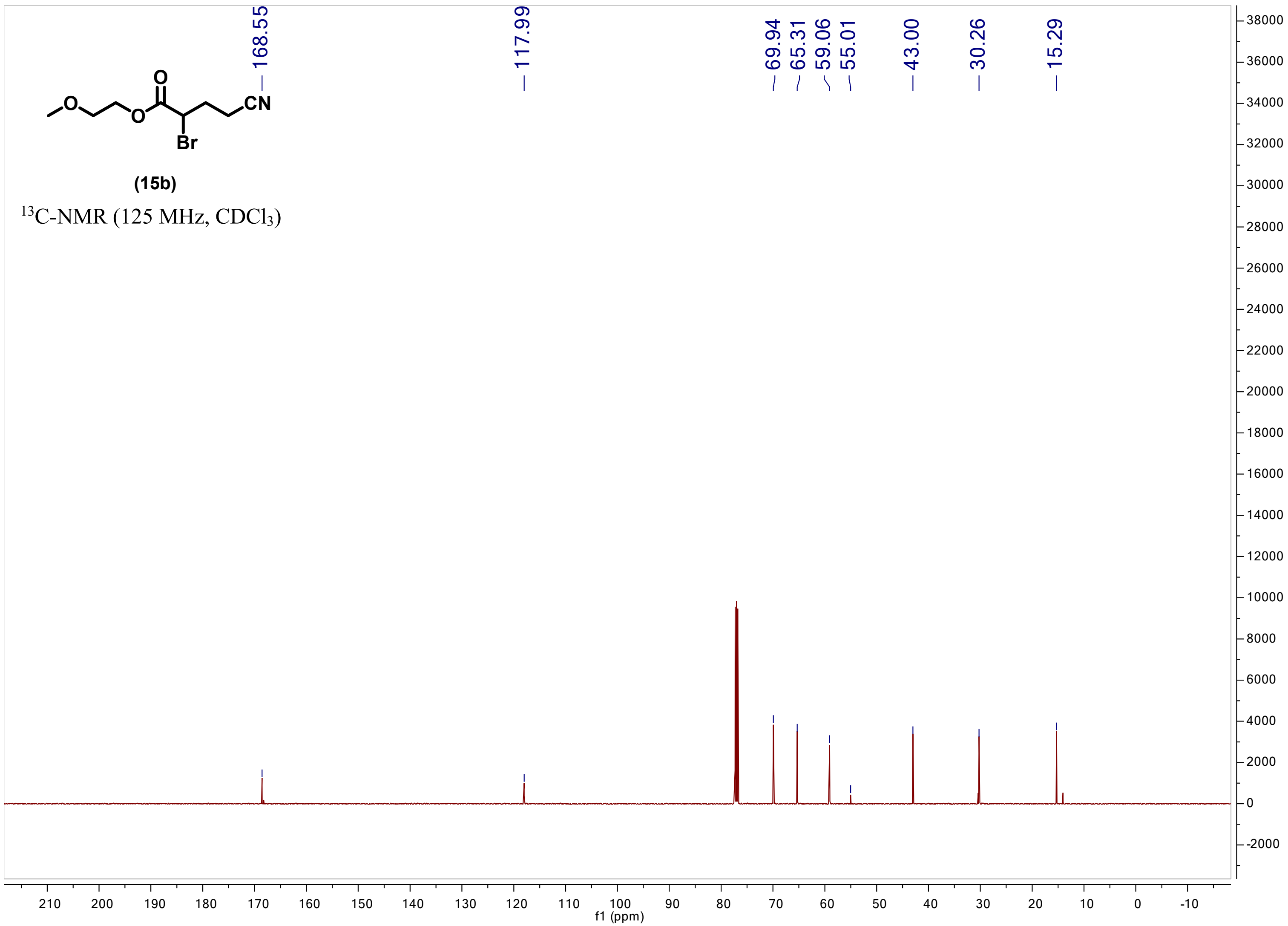

168.55
117.99
69.94
65.31
59.06
55.01
43.00
30.26
15.29
O
O
O
CN
Br
(15b)
13C-NMR (125 MHz, CDCl3)
f1 (ppm)

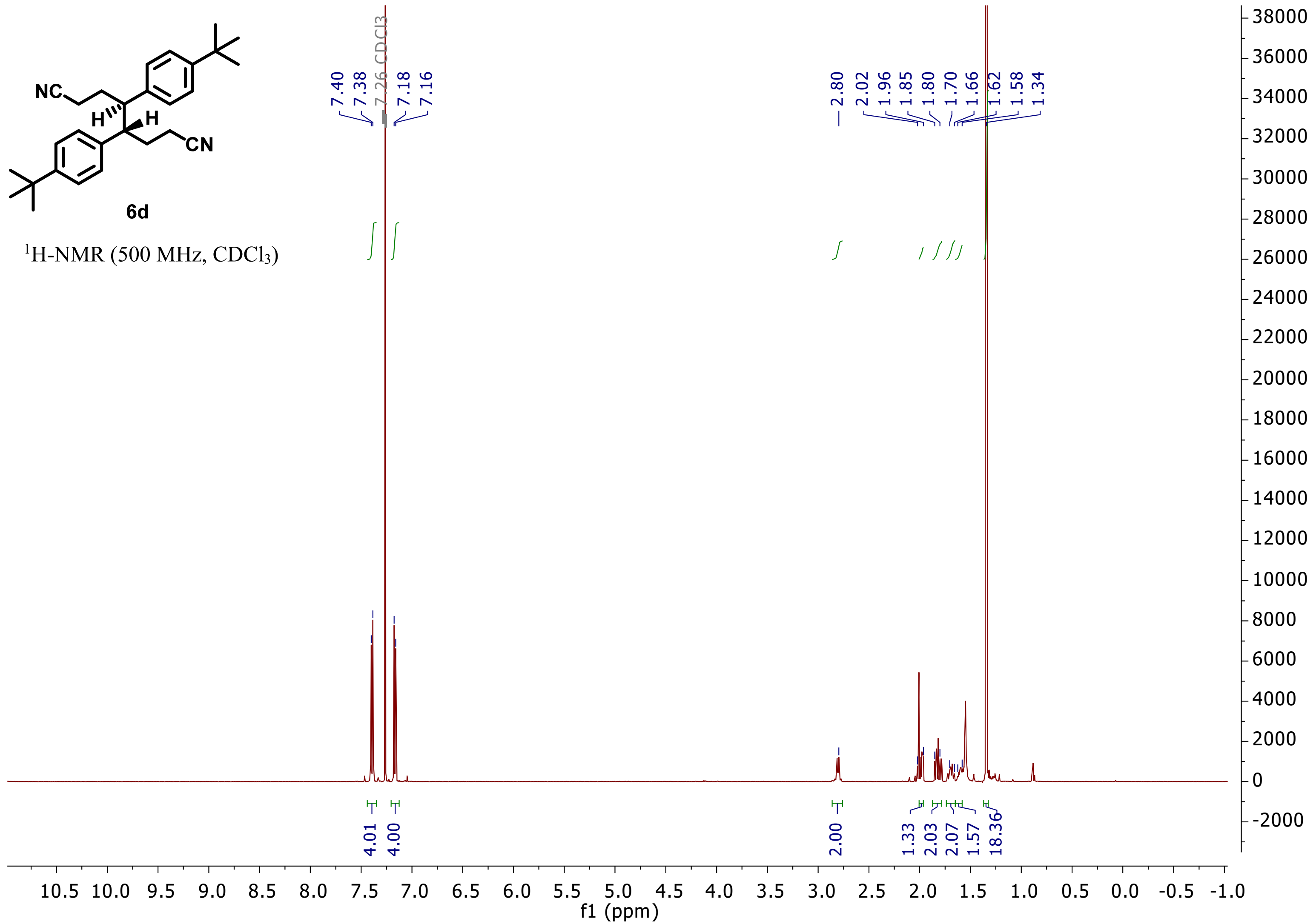

^{1}H-NMR (500 MHz, $CDCl_3$)

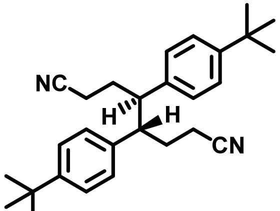

6d

^{13}C-NMR (125 MHz, $CDCl_3$)

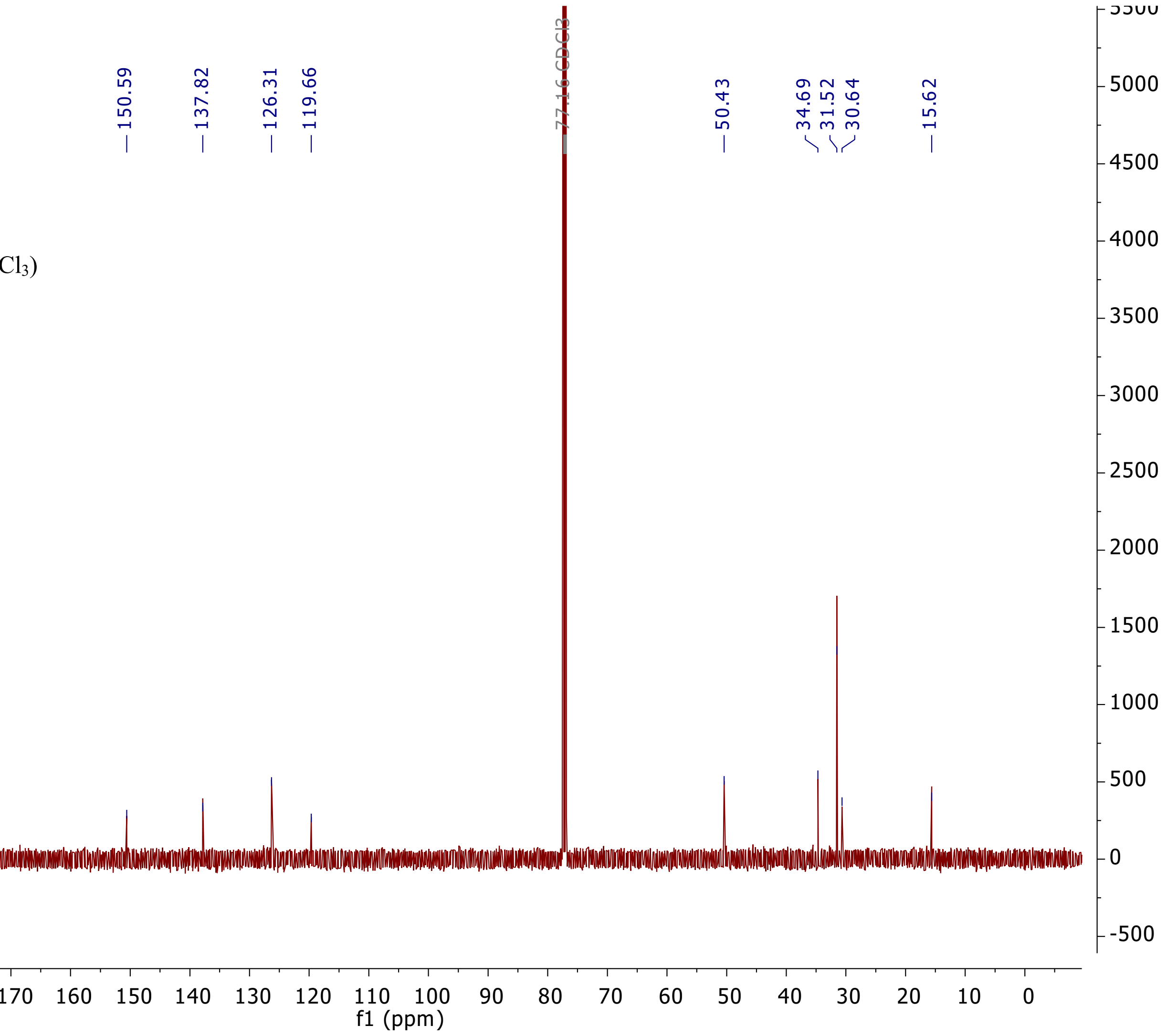

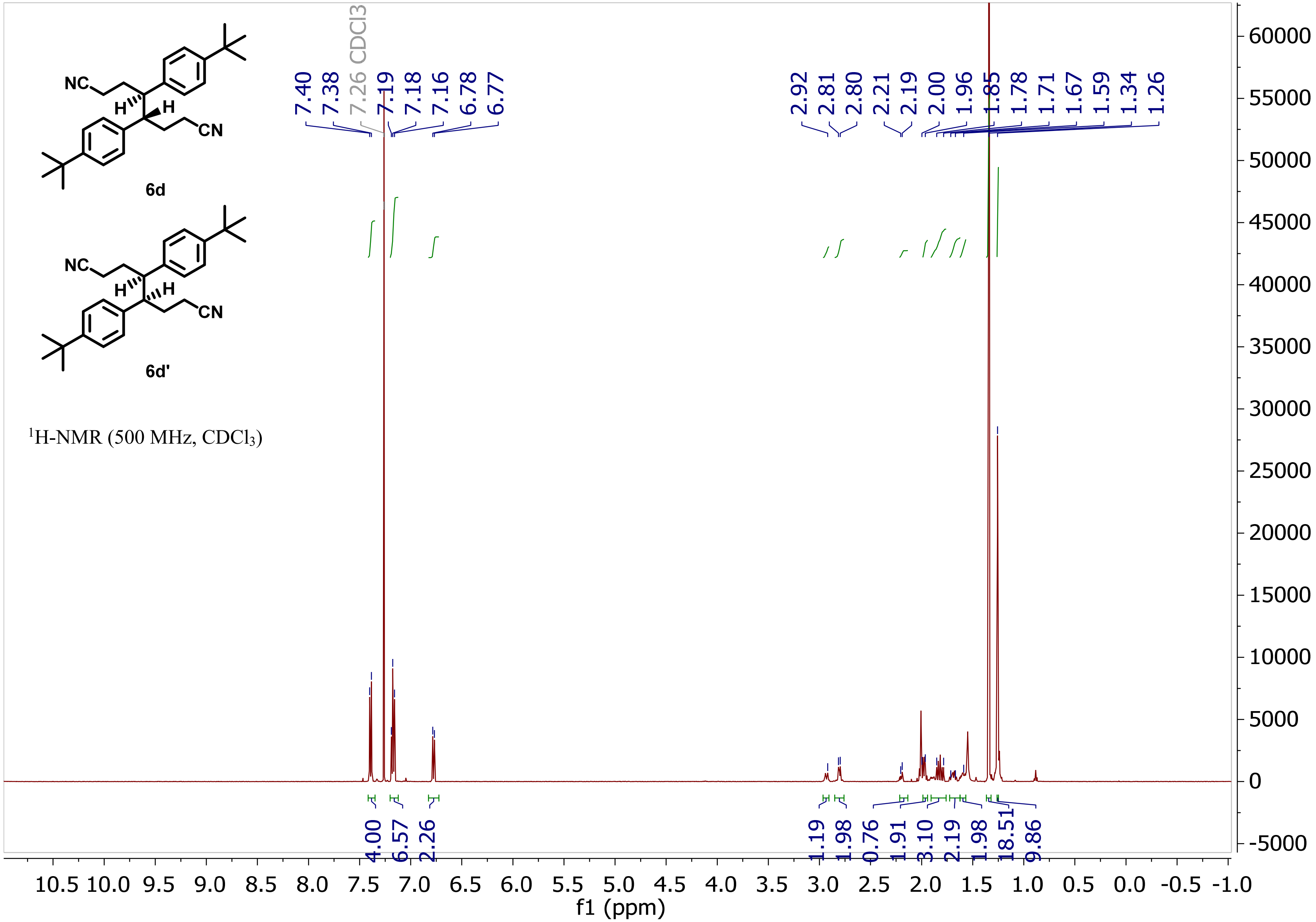

^{1}H-NMR (500 MHz, $CDCl_3$)

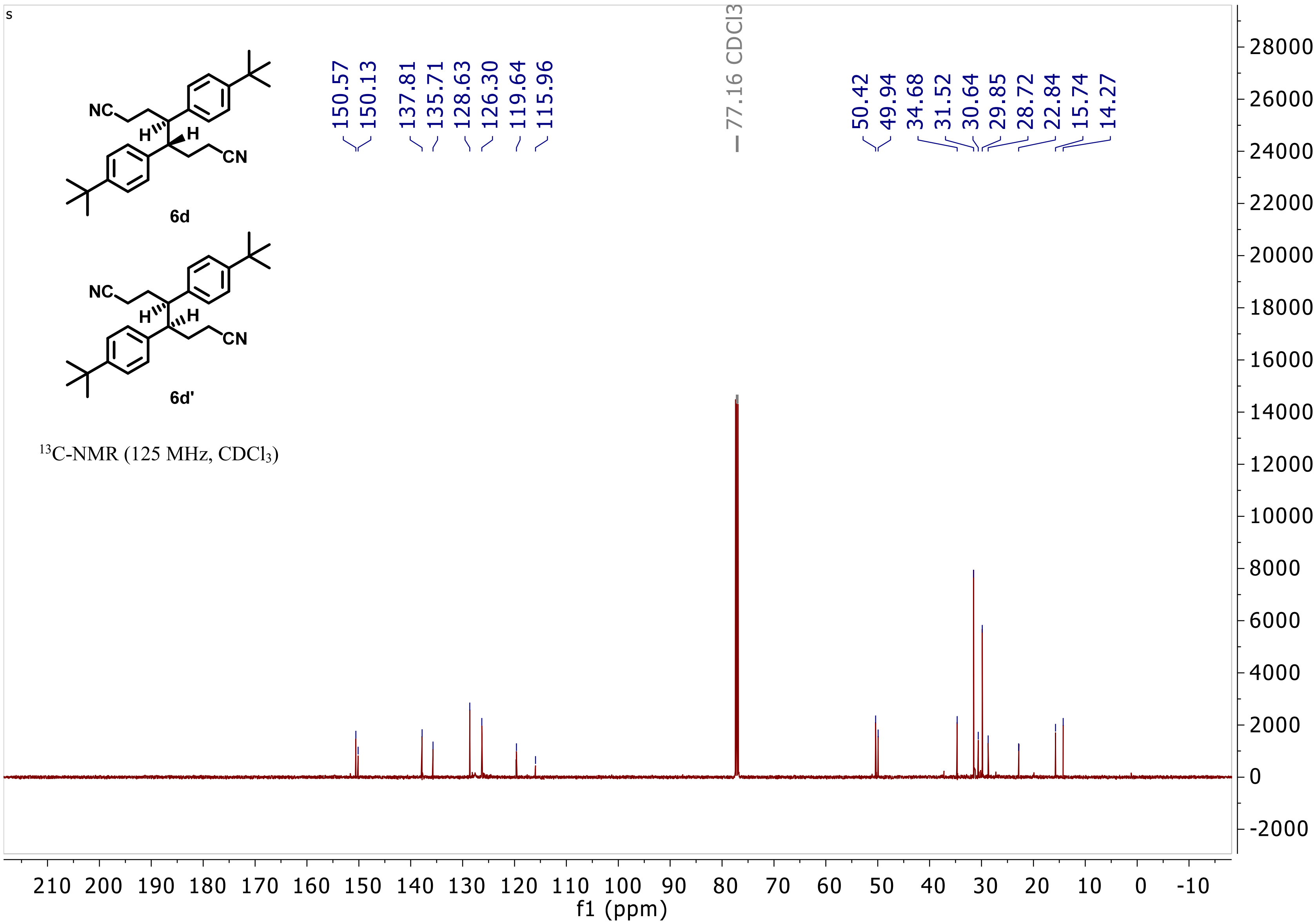

s
NC
H
H
CN
6d
NC
H
H
CN
6d'
13C-NMR (125 MHz, CDCl3)
150.57
150.13
137.81
135.71
128.63
126.30
119.64
115.96
77.16 CDCl3
50.42
49.94
34.68
31.52
30.64
29.85
28.72
22.84
15.74
14.27
f1 (ppm)

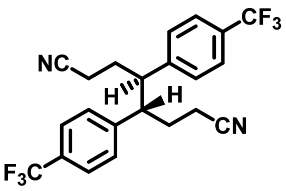

7d

^{1}H-NMR (500 MHz, $CDCl_3$)

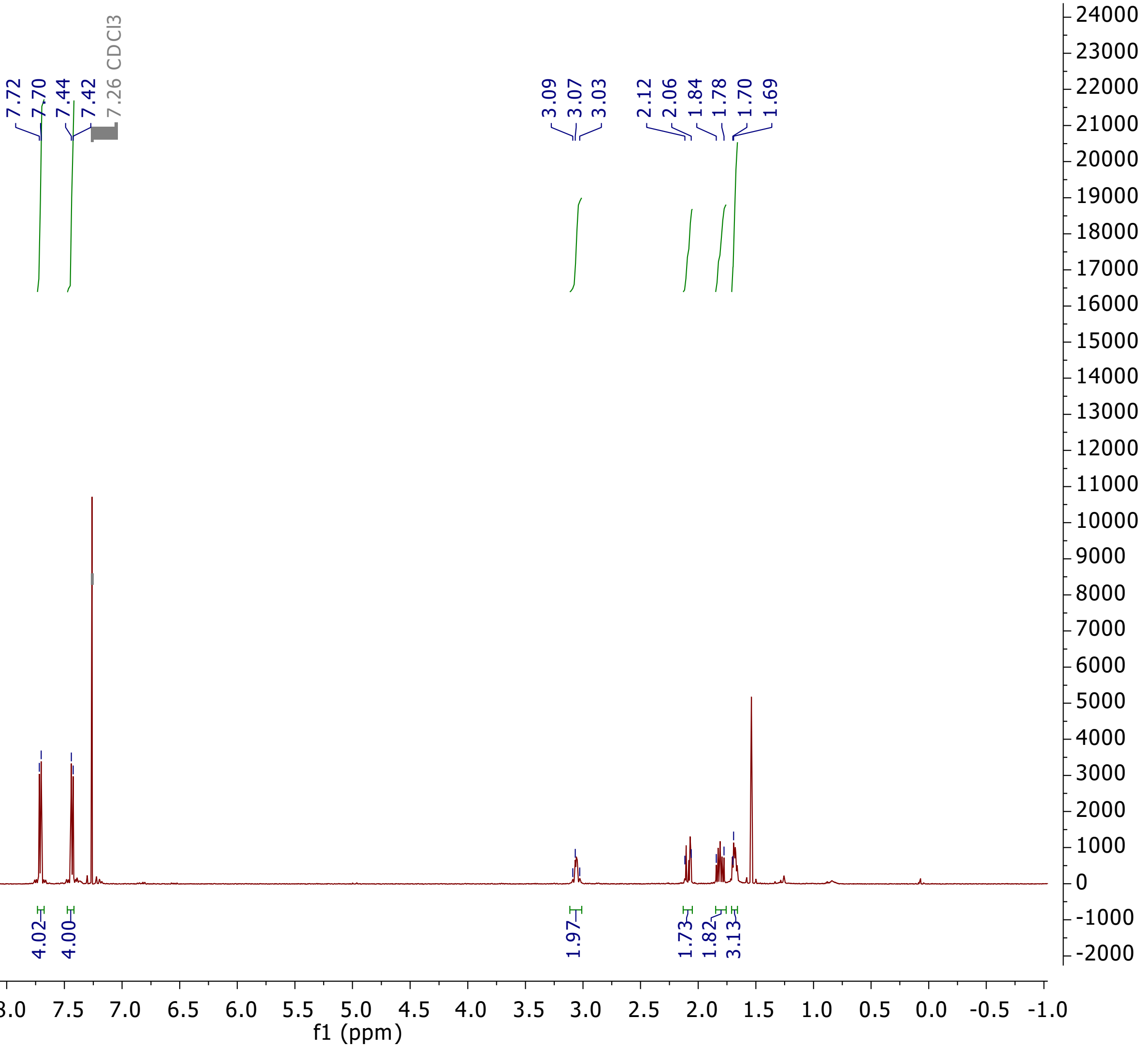

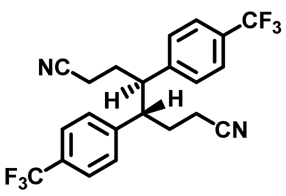

7d

^{13}C-NMR (125 MHz, $CDCl_3$)

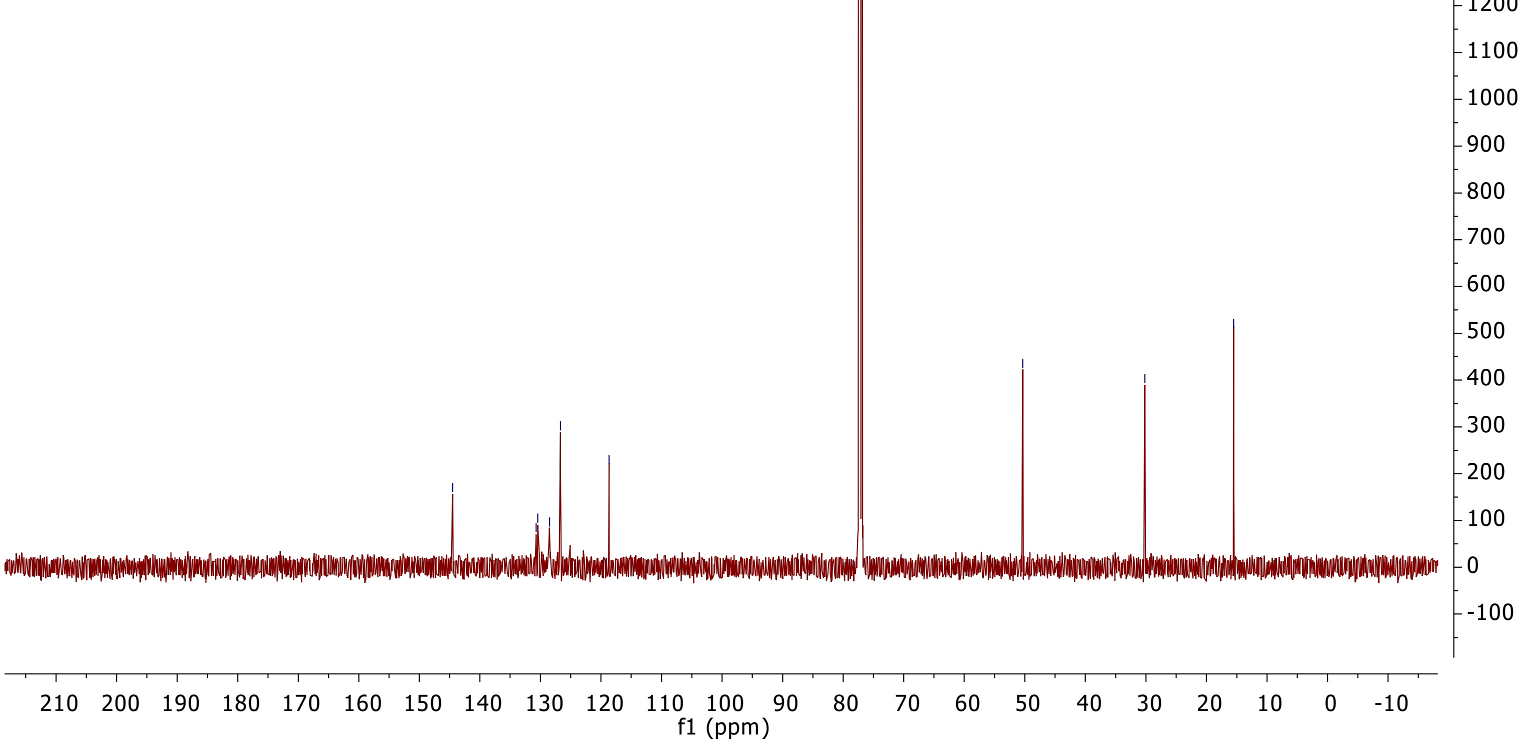

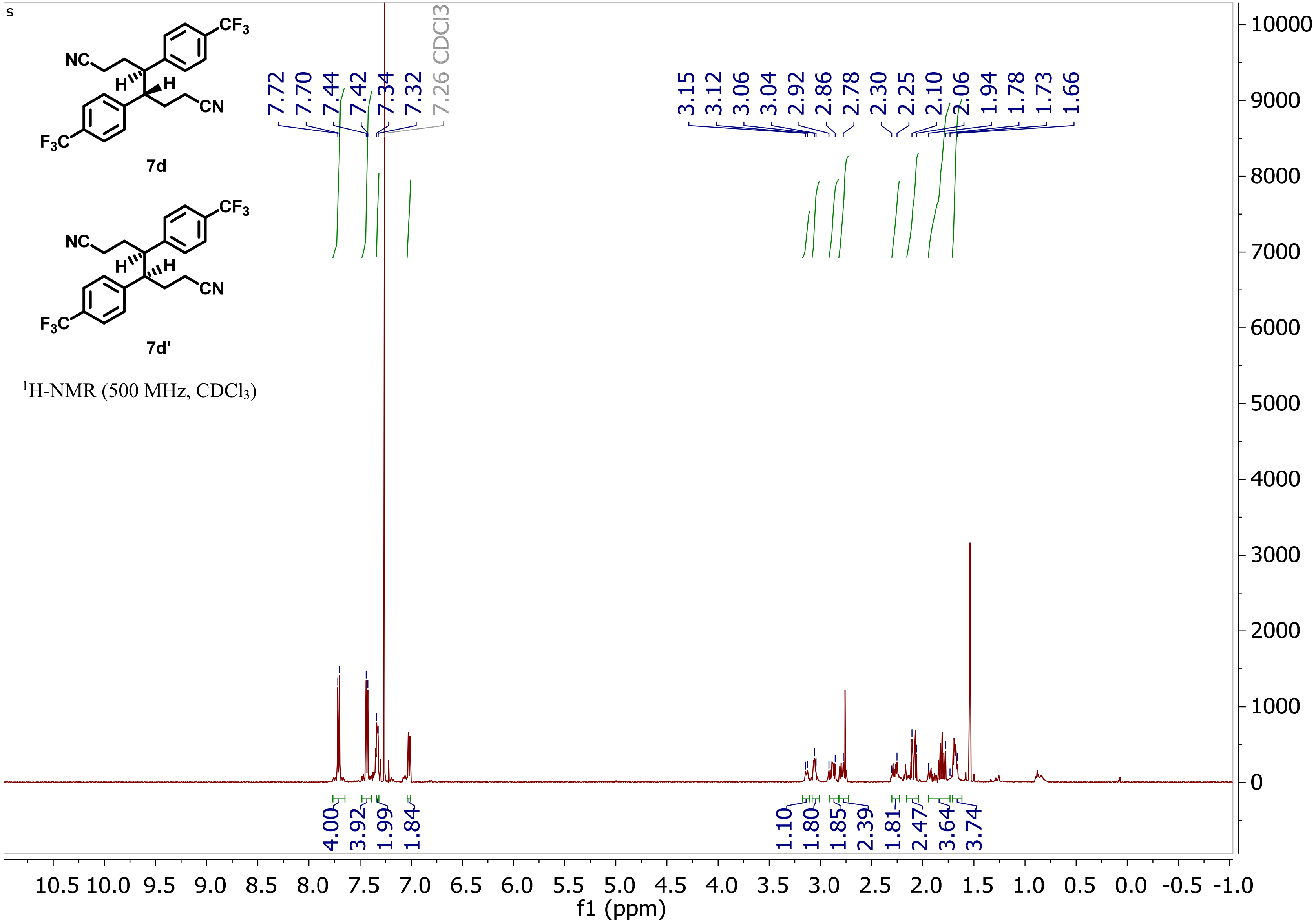

^{1}H-NMR (500 MHz, $CDCl_3$)

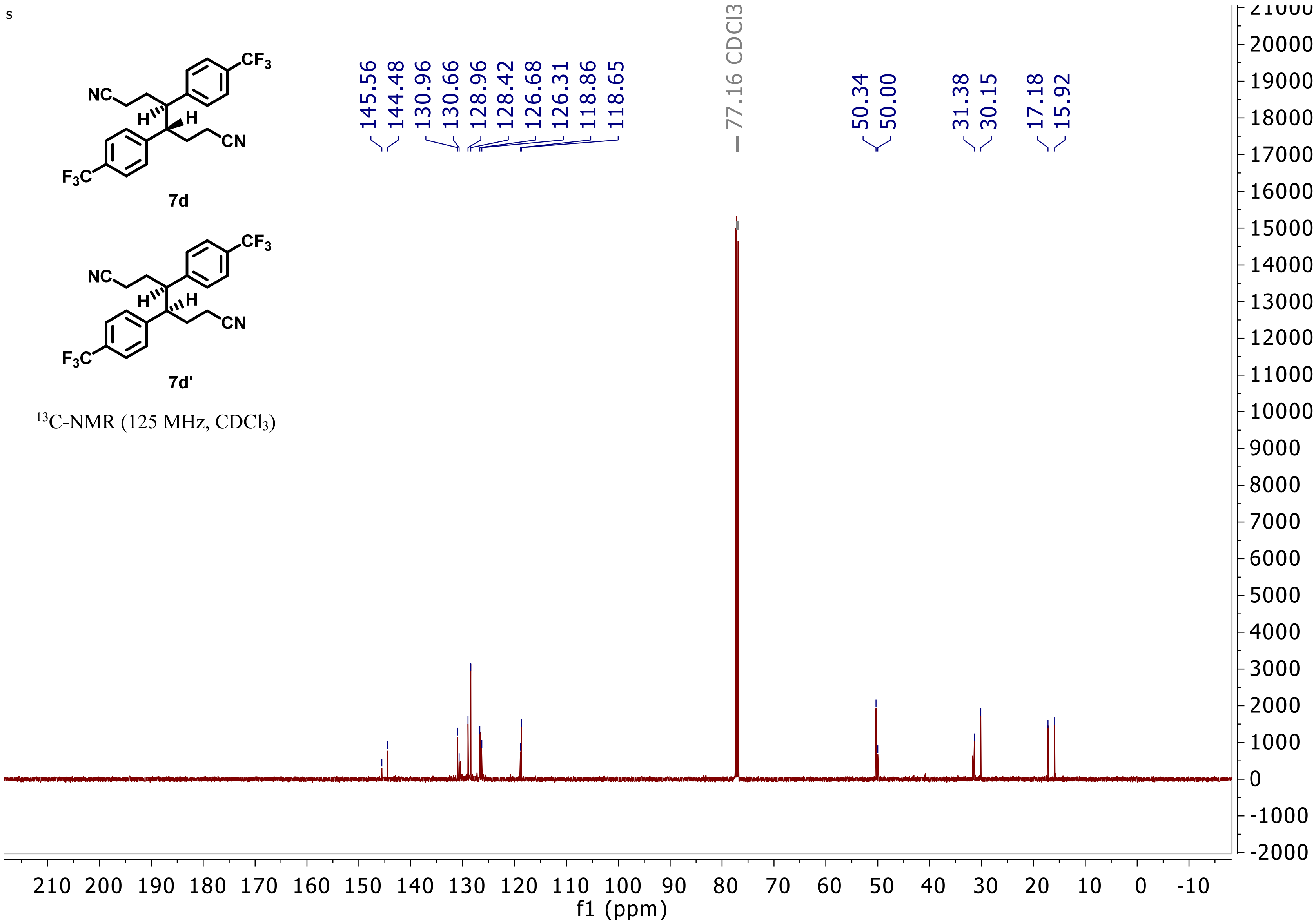

7d
7d'
^{13}C-NMR (125 MHz, CDCl$_3$)
145.56
144.48
130.96
130.66
128.96
128.42
126.68
126.31
118.86
118.65
77.16 CDCl3
50.34
50.00
31.38
30.15
17.18
15.92
f1 (ppm)

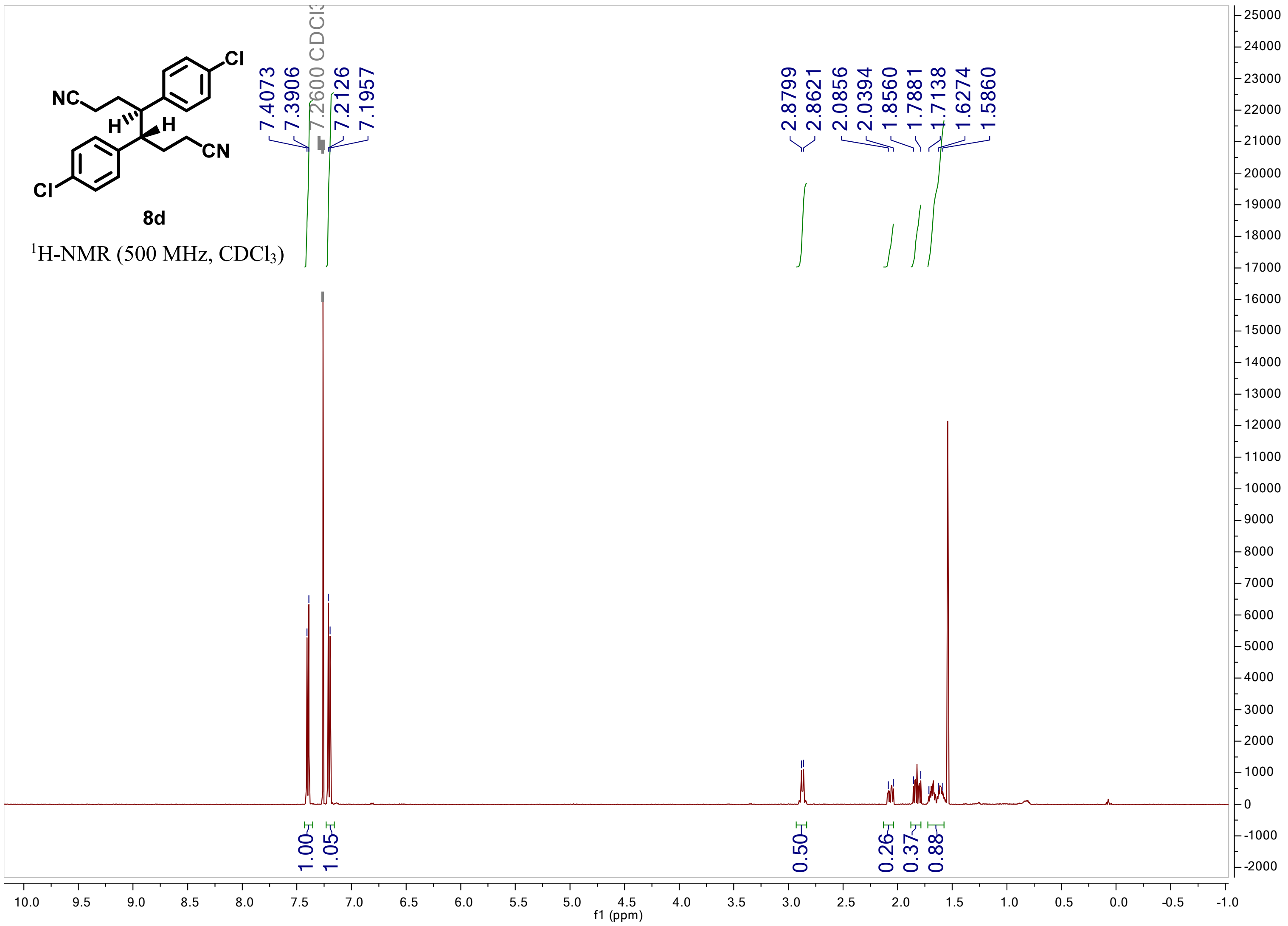
8d
^{1}H-NMR (500 MHz, $CDCl_3$)
7.4073
7.3906
7.2600 CDCl3
7.2126
7.1957
2.8799
2.8621
2.0856
2.0394
1.8560
1.7881
1.7138
1.6274
1.5860
1.00
1.05
0.50
0.26
0.37
0.88
f1 (ppm)

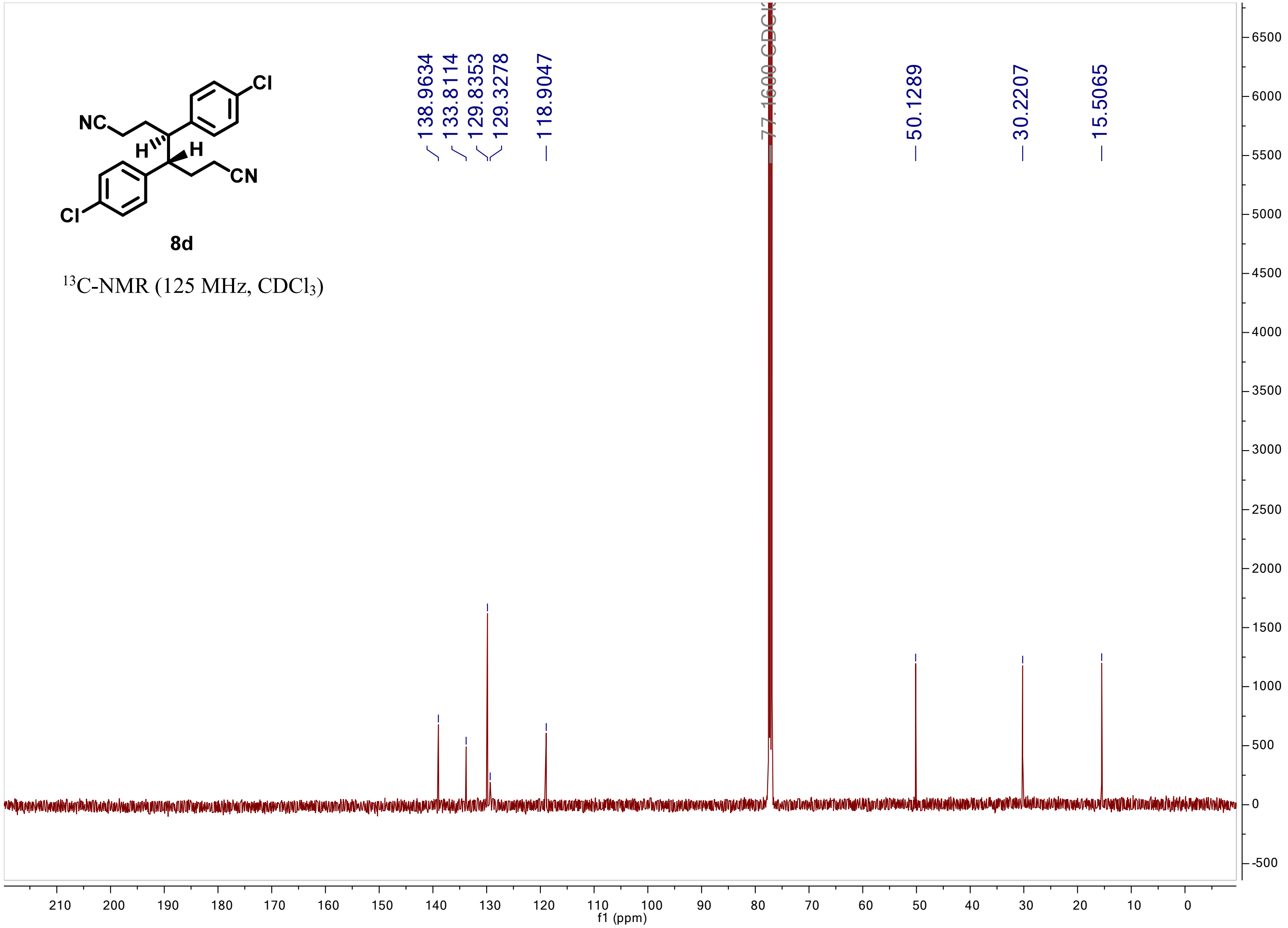

8d

^{13}C-NMR (125 MHz, $CDCl_3$)

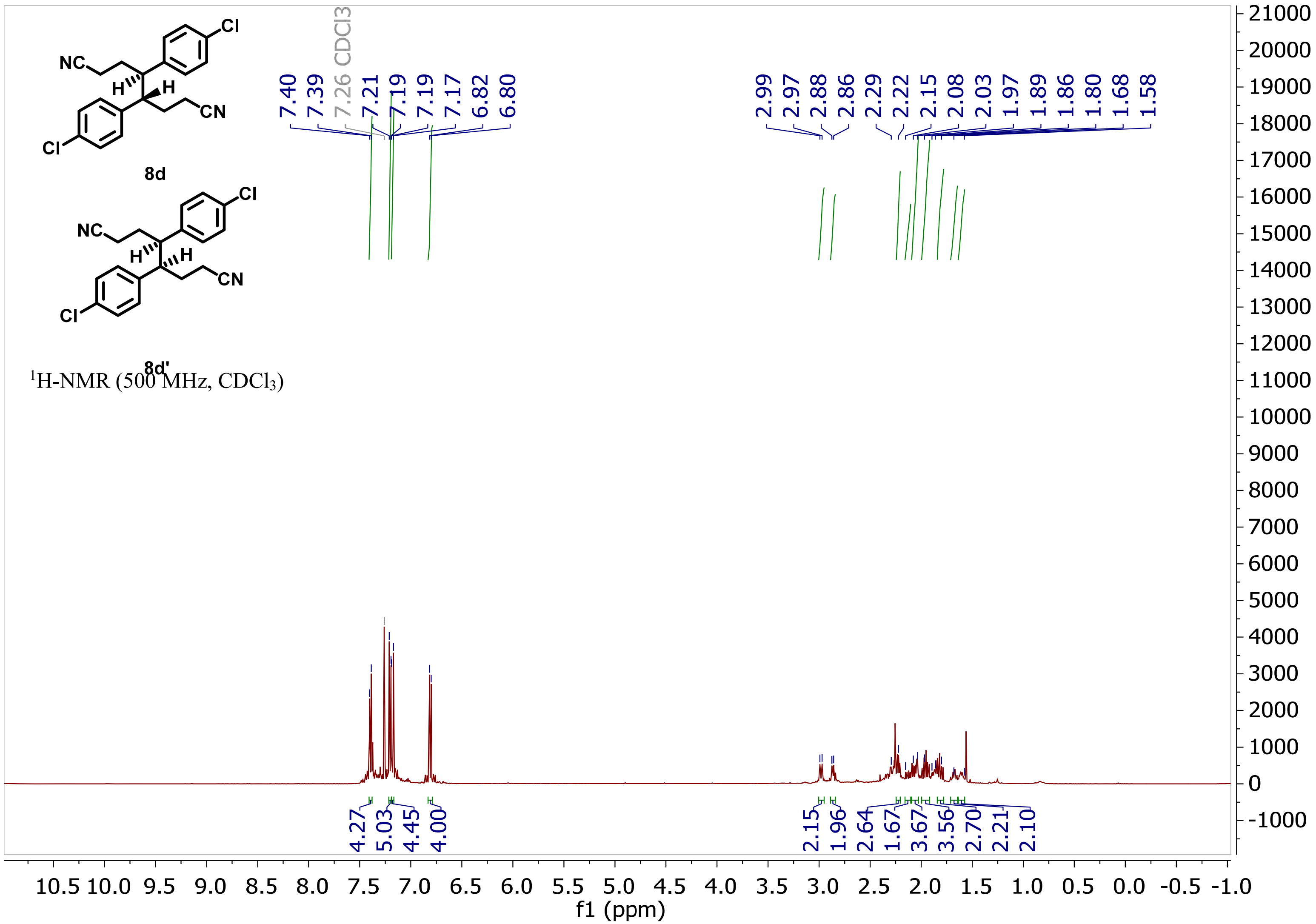
8d
8d'
^{1}H-NMR (500 MHz, $CDCl_3$)
7.40
7.39
7.26 CDCl3
7.21
7.19
7.19
7.17
6.82
6.80
2.99
2.97
2.88
2.86
2.29
2.22
2.15
2.08
2.03
1.97
1.89
1.86
1.80
1.68
1.58
4.27
5.03
4.45
4.00
2.15
1.96
2.64
1.67
3.67
3.56
2.70
2.21
2.10
f1 (ppm)

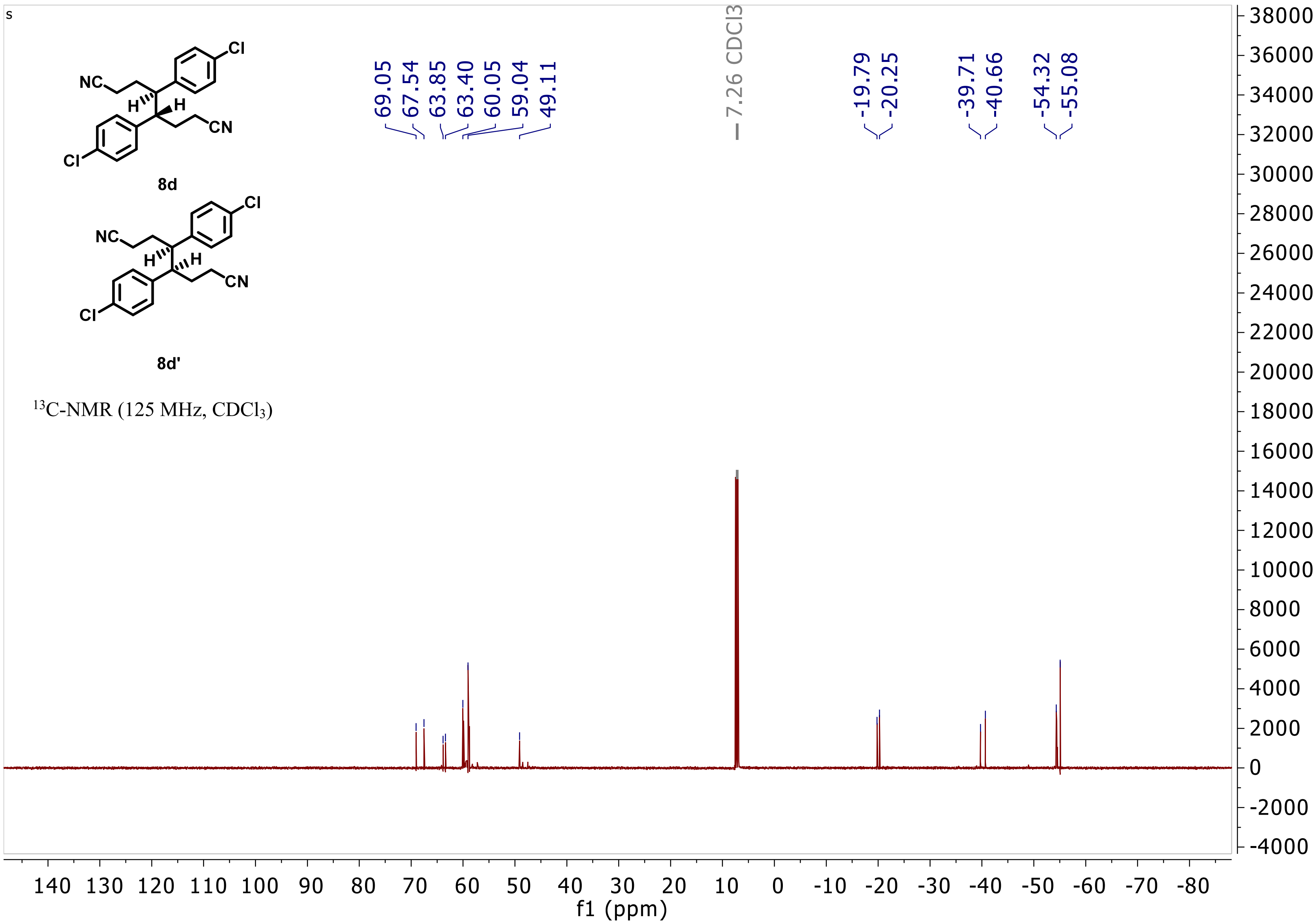
8d
8d'
^{13}C-NMR (125 MHz, $CDCl_3$)
69.05
67.54
63.85
63.40
60.05
59.04
49.11
7.26 CDCl3
-19.79
-20.25
-39.71
-40.66
-54.32
-55.08
f1 (ppm)

9. Control Electrochemical Experiments

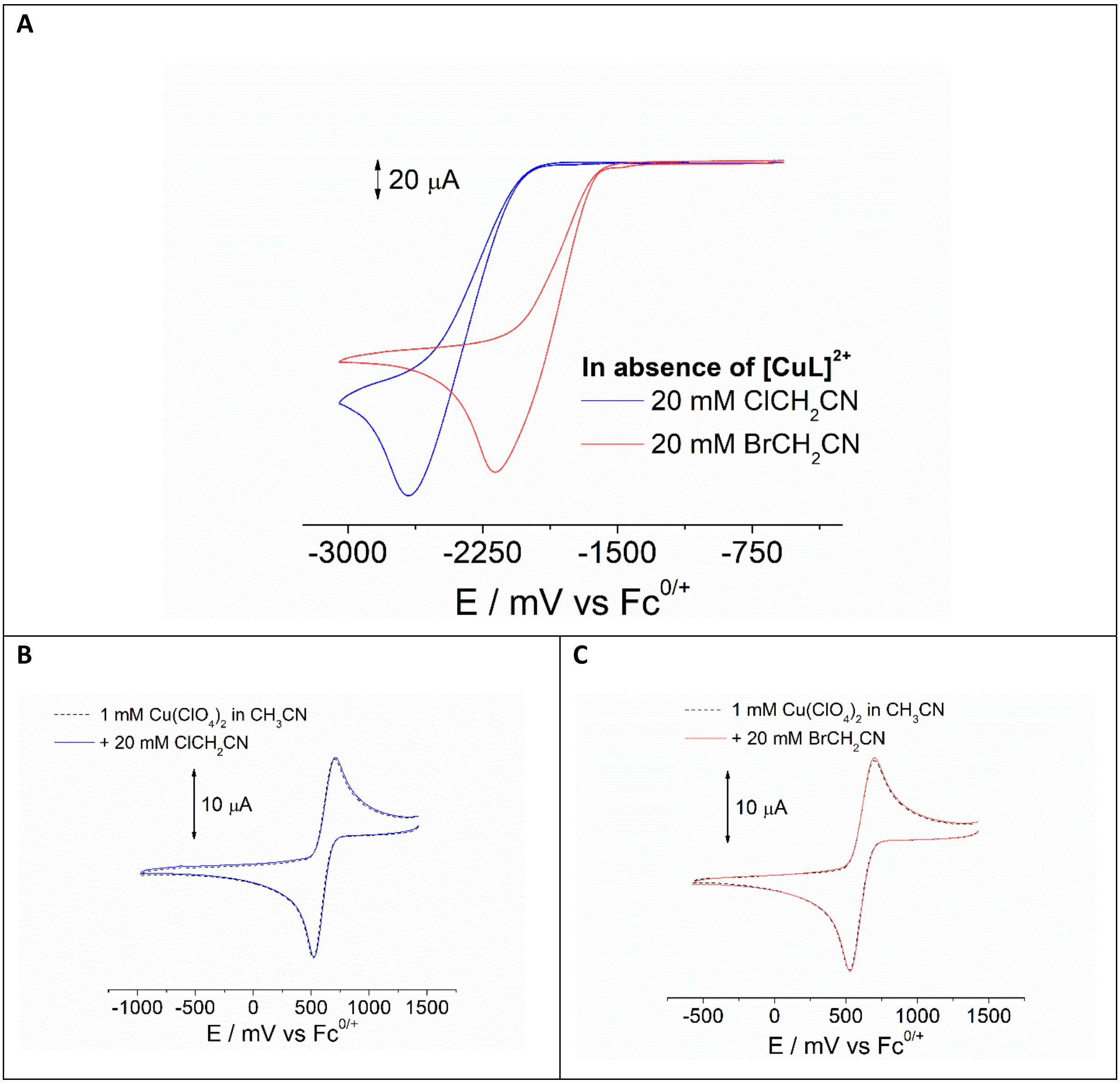

Figure S3. Cyclic voltammetry (scan rate 100 mV s^{-1}, electrolyte 0.1 M Et_4NClO_4 in CH_3CN) of (**A**) $ClCH_2CN$ (20 mM, blue curve) and $BrCH_2CN$ (20, mM, red curve); (**B**) $[Cu(CH_3CN)_4]^{2+}$ (1 mM, broken curve) and $[Cu(CH_3CN)_4]^{2+}$ (1 mM) + 20 mM $ClCH_2CN$ (solid curve); (**C**) $[Cu(CH_3CN)_4]^{2+}$ (1 mM, broken curve) and $[Cu(CH_3CN)_4]^{2+}$ (1 mM) + 20 mM $BrCH_2CN$ (solid curve)

10. X-Ray Crystal Structures

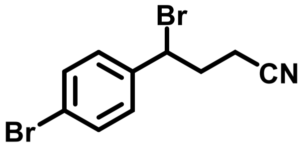

(9b)

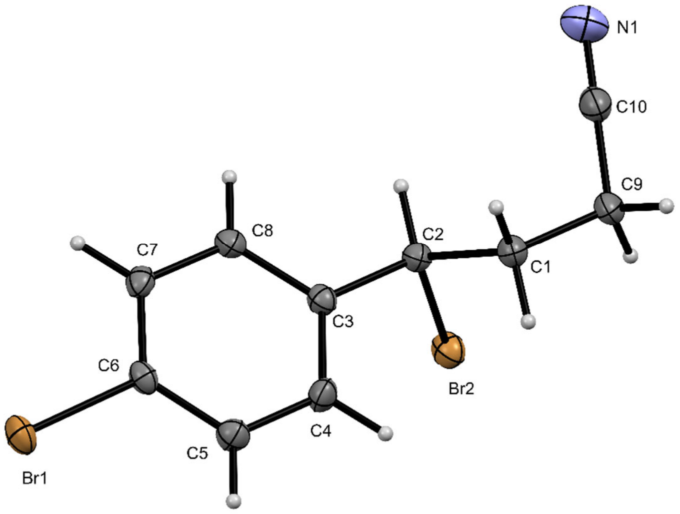

Empirical formula	$C_{10} H_9 Br_2 N$	
Formula weight	303.00	
Temperature	190(2) K	
Wavelength	1.54184 Å	
Crystal system	Monoclinic	
Space group	$P\ 2_1/c$	
Unit cell dimensions	a = 6.3061(2) Å	α= 90°.
	b = 13.0601(4) Å	β= 99.138(4)°.
	c = 13.0615(5) Å	γ = 90°.
Volume	1062.07(6) $Å^3$	
Z	4	
Density (calculated)	1.895 Mg/m^3	
Absorption coefficient	9.294 mm^{-1}	
F(000)	584	
Crystal size	0.1 x 0.05 x 0.02 mm^3	
Theta range for data collection	4.819 to 61.704°.	
Index ranges	-7<=h<=7, -14<=k<=10, -12<=l<=14	
Reflections collected	4137	
Independent reflections	1645 [R(int) = 0.0331]	
Completeness to theta = 61.704°	98.9 %	
Absorption correction	Semi-empirical from equivalents	
Max. and min. transmission	1 and 0.798	

Data / restraints / parameters	1645 / 0 / 118
Goodness-of-fit on F^2	1.061
Final R indices [I>2sigma(I)]	R1 = 0.0280, wR2 = 0.0636
R indices (all data)	R1 = 0.0332, wR2 = 0.0664
Largest diff. peak and hole	0.470 and -0.368 e.$Å^{-3}$

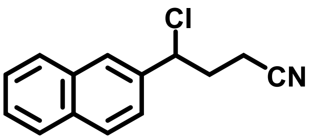

(12a)

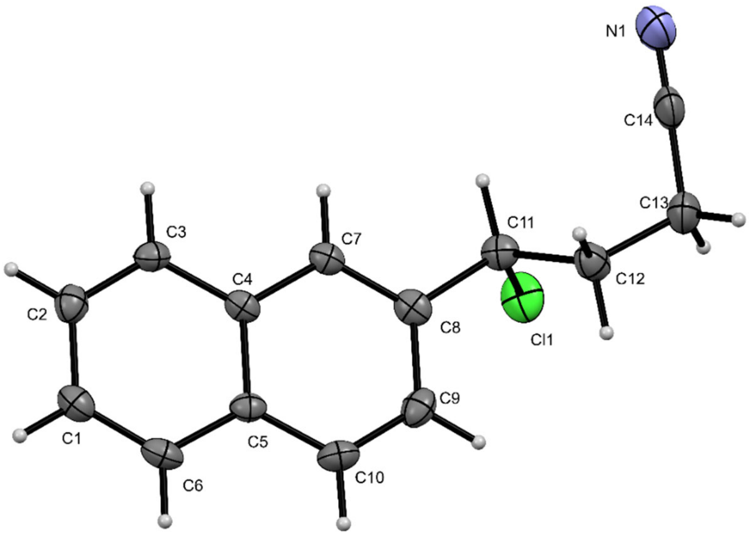

Empirical formula	C_{14} H_{12} Cl N	
Formula weight	229.70	
Temperature	190(2) K	
Wavelength	1.54184 Å	
Crystal system	Orthorhombic	
Space group	*P n a* 2_1	
Unit cell dimensions	a = 11.6527(6) Å	α= 90°.
	b = 16.6170(8) Å	β= 90°.
	c = 5.9865(4) Å	γ = 90°.
Volume	1159.2(1) Å^3	
Z	4	
Density (calculated)	1.316 Mg/m^3	
Absorption coefficient	2.650 mm^{-1}	
F(000)	480	
Crystal size	0.4 x 0.05 x 0.04 mm^3	
Theta range for data collection	4.635 to 61.428°.	
Index ranges	-12<=h<=13, -18<=k<=13, -6<=l<=5	
Reflections collected	2672	
Independent reflections	1403 [R(int) = 0.0371]	
Completeness to theta = 61.428°	99.4 %	
Absorption correction	Semi-empirical from equivalents	
Max. and min. transmission	1 and 0.94	
Data / restraints / parameters	1403 / 1 / 145	

Goodness-of-fit on F^2	1.050
Final R indices [I>2sigma(I)]	R1 = 0.0497, wR2 = 0.1205
R indices (all data)	R1 = 0.0599, wR2 = 0.1298
Absolute structure parameter	0.03(4)
Largest diff. peak and hole	0.496 and -0.302 e.Å^{-3}

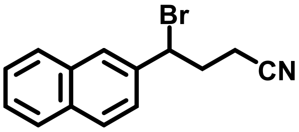

(12b)

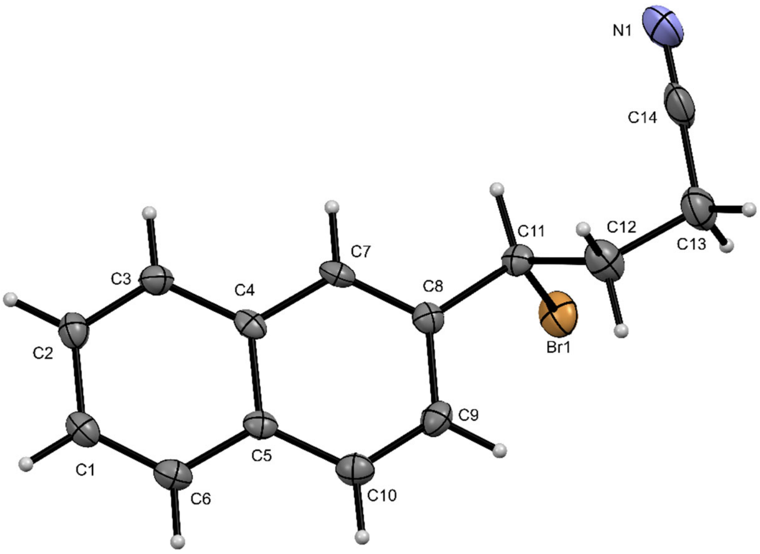

Empirical formula	$C_{14} H_{12} Br N$	
Formula weight	274.16	
Temperature	190(2) K	
Wavelength	1.54184 Å	
Crystal system	Orthorhombic	
Space group	$P\,n\,a\,2_1$	
Unit cell dimensions	a = 11.5971(6) Å	α= 90°.
	b = 17.179(1) Å	β= 90°.
	c = 5.9922(3) Å	γ = 90°.
Volume	1193.8(1) $Å^3$	
Z	4	
Density (calculated)	1.525 Mg/m^3	
Absorption coefficient	4.436 mm^{-1}	
F(000)	552	
Crystal size	0.200 x 0.020 x 0.020 mm^3	
Theta range for data collection	4.600 to 61.486°.	
Index ranges	-13<=h<=10, -13<=k<=19, -4<=l<=6	
Reflections collected	2736	
Independent reflections	1382 [R(int) = 0.0371]	
Completeness to theta = 61.486°	99.8 %	
Absorption correction	Semi-empirical from equivalents	
Max. and min. transmission	1 and 0.881	
Data / restraints / parameters	1382 / 1 / 145	

Goodness-of-fit on F^2	1.070
Final R indices [I>2sigma(I)]	R1 = 0.0342, wR2 = 0.0787
R indices (all data)	R1 = 0.0389, wR2 = 0.0822
Absolute structure parameter	0.04(5)
Largest diff. peak and hole	0.339 and -0.270 e.Å^{-3}

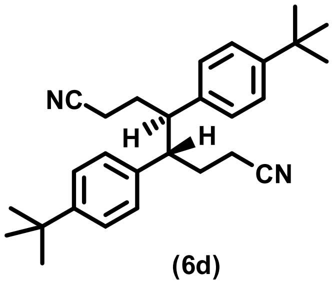

(6d)

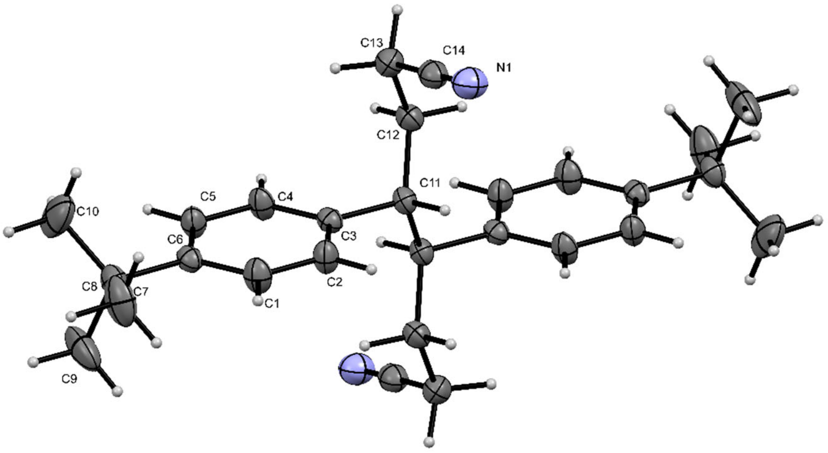

Empirical formula	$C_{28} H_{36} N_2$	
Formula weight	400.59	
Temperature	190(2) K	
Wavelength	1.54184 Å	
Crystal system	Triclinic	
Space group	$P\ \bar{1}$	
Unit cell dimensions	a = 6.2887(6) Å	α= 73.72(1)°.
	b = 9.710(1) Å	β= 77.52(1)°.
	c = 10.801(1) Å	γ = 79.838(8)°.
Volume	613.4(1) $Å^3$	
Z	1	
Density (calculated)	1.084 Mg/m^3	
Absorption coefficient	0.471 mm^{-1}	
F(000)	218	
Crystal size	0.4 x 0.1 x 0.01 mm^3	
Theta range for data collection	4.332 to 61.543°.	
Index ranges	-6<=h<=6, -11<=k<=11, -12<=l<=12	
Reflections collected	3087	
Independent reflections	3087 [R(int) = 0.0592]	
Completeness to theta = 61.543°	98.5 %	
Absorption correction	Semi-empirical from equivalents	
Max. and min. transmission	1 and 0.983	

Data / restraints / parameters	3087 / 0 / 137
Goodness-of-fit on F^2	0.813
Final R indices [I>2sigma(I)]	R1 = 0.0502, wR2 = 0.1062
R indices (all data)	R1 = 0.0947, wR2 = 0.1168
Largest diff. peak and hole	0.211 and -0.124 e.$Å^{-3}$

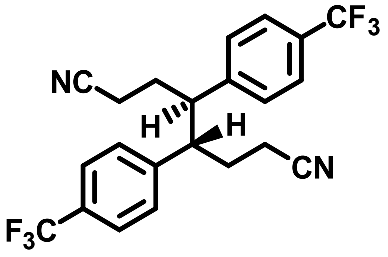

(7d)

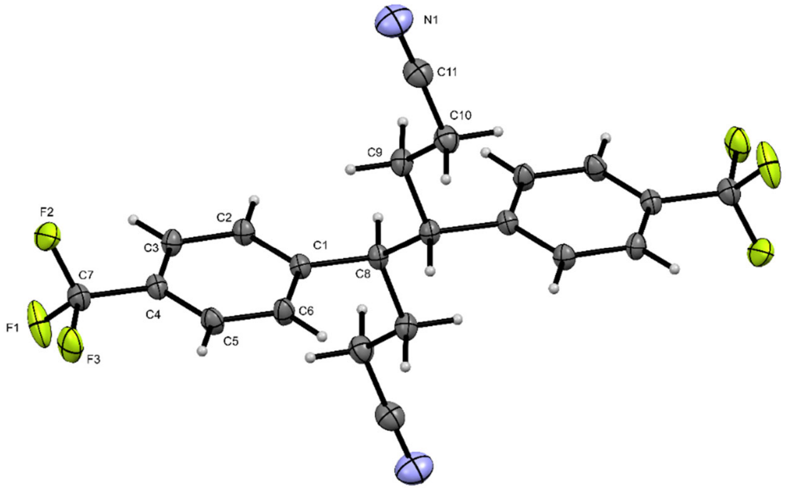

Empirical formula	$C_{22} H_{18} F_6 N_2$	
Formula weight	424.38	
Temperature	190(2) K	
Wavelength	1.54184 Å	
Crystal system	Monoclinic	
Space group	$P\ 2_1/c$	
Unit cell dimensions	a = 10.666(1) Å	α= 90°.
	b = 12.085(1) Å	β= 108.74(1)°.
	c = 8.0892(9) Å	γ = 90°.
Volume	987.41(19) $Å^3$	
Z	2	
Density (calculated)	1.427 Mg/m^3	
Absorption coefficient	1.078 mm^{-1}	
F(000)	436	
Crystal size	0.200 x 0.200 x 0.080 mm^3	
Theta range for data collection	4.377 to 62.487°.	
Index ranges	-12<=h<=12, -13<=k<=13, -9<=l<=9	
Reflections collected	2718	
Independent reflections	2718 [R(int) = 0.022]	
Completeness to theta = 62.487°	100.0 %	
Absorption correction	Semi-empirical from equivalents	
Max. and min. transmission	1 and 0.936	
Data / restraints / parameters	2718 / 0 / 150	
Goodness-of-fit on F^2	1.067	

Final R indices [I>2sigma(I)]	R1 = 0.0399, wR2 = 0.1113
R indices (all data)	R1 = 0.0478, wR2 = 0.1145
Largest diff. peak and hole	0.198 and -0.258 e.$Å^{-3}$

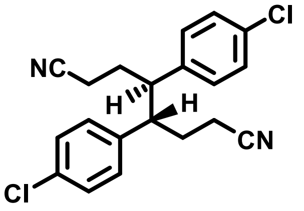

(8d)

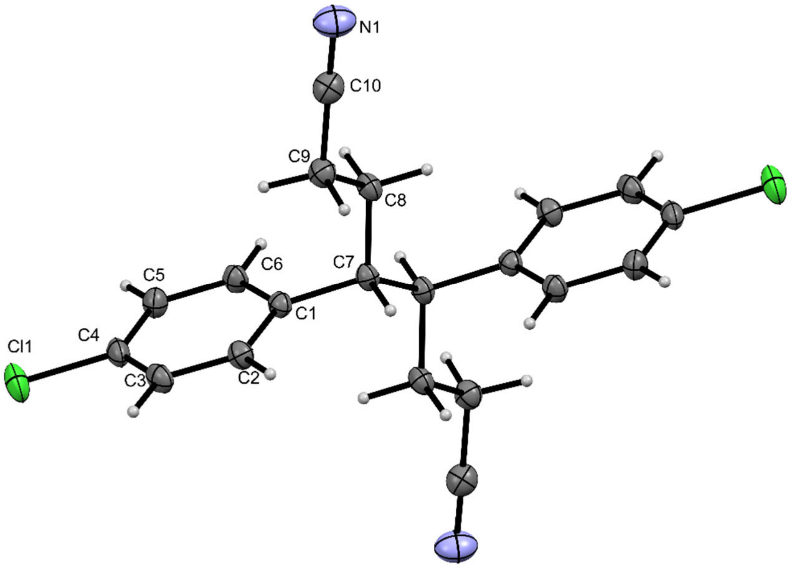

Empirical formula	$C_{20} H_{18} Cl_2 N_2$	
Formula weight	357.26	
Temperature	190(2) K	
Wavelength	1.54184 Å	
Crystal system	Monoclinic	
Space group	$P\ 2_1/c$	
Unit cell dimensions	a = 10.5854(2) Å	α= 90°.
	b = 11.8999(2) Å	β= 110.688(2)°.
	c = 7.6750(1) Å	γ = 90°.
Volume	904.44(3) $Å^3$	
Z	2	
Density (calculated)	1.312 Mg/m^3	
Absorption coefficient	3.236 mm^{-1}	
F(000)	372	
Crystal size	0.300 x 0.150 x 0.100 mm^3	
Theta range for data collection	4.465 to 62.486°.	
Index ranges	-12<=h<=12, -13<=k<=13, -8<=l<=8	
Reflections collected	12699	
Independent reflections	1443 [R(int) = 0.0290]	
Completeness to theta = 62.486°	100.0 %	
Absorption correction	Semi-empirical from equivalents	

Max. and min. transmission	1 and 0.707
Data / restraints / parameters	1443 / 0 / 109
Goodness-of-fit on F^2	1.039
Final R indices [I>2sigma(I)]	R1 = 0.0302, wR2 = 0.0803
R indices (all data)	R1 = 0.0308, wR2 = 0.0807
Largest diff. peak and hole	0.232 and -0.233 e.Å^{-3}

11. References

1. G. M. Sheldrick, *Acta Cryst. Sect. A*, 2008, **64**, 112-122.
2. C. F. Macrae, P. R. Edgington, P. McCabe, E. Pidcock, G. P. Shields, R. Taylor, M. Towler and J. van de Streek, *J. Appl. Crystallogr.*, 2006, **39**, 453-457.
3. L. J. Farrugia, *J. Appl. Crystallogr.*, 1999, **32**, 837-838.
4. T. J. Zerk and P. V. Bernhardt, *Inorg. Chem.*, 2017, **56**, 5784-5792.
5. S. Haubenreisser, T. H. Wöste, C. Martínez, K. Ishihara and K. Muñiz, *Angew. Chem. Int. Ed.*, 2016, **55**, 413-417.
6. B. N. Bhawal, J. C. Reisenbauer, C. Ehinger and B. Morandi, *J. Am. Chem. Soc.*, 2020, **142**, 10914-10920.
7. T. Iwasaki, Y. Miyata, R. Akimoto, Y. Fujii, H. Kuniyasu and N. Kambe, *J. Am. Chem. Soc.*, 2014, **136**, 9260-9263.
8. M. Davi and H. Lebel, *Org. Lett.*, 2009, **11**, 41-44.
9. N. Parveen and G. Sekar, *J. Org. Chem.*, 2020, **85**, 4682-4694.
10. R. Wang, Y. Chen, M. Shu, W. Zhao, M. Tao, C. Du, X. Fu, A. Li and Z. Lin, *Chem. Eur. J.*, 2020, **26**, 1941-1946.
11. X. Tian, T. A. Karl, S. Reiter, S. Yakubov, R. de Vivie-Riedle, B. König and J. P. Barham, *Angew. Chem. Int. Ed.*, 2021, **60**, 20817-20825.
12. T. M. Gøgsig, L. S. Søbjerg, A. T. Lindhardt, K. L. Jensen and T. Skrydstrup, *J. Org. Chem.*, 2008, **73**, 3404-3410.
13. M. A. Gonzálvez, J. R. Harmer and P. V. Bernhardt, *Inorg. Chem.*, 2021, **60**, 10648-10655.
14. V. S. Kostromitin, A. A. Zemtsov, V. A. Kokorekin, V. V. Levin and A. D. Dilman, *Chem. Commun.*, 2021, **57**, 5219-5222.
15. X.-Y. Yu, J. Chen, H.-W. Chen, W.-J. Xiao and J.-R. Chen, *Org. Lett.*, 2020, **22**, 2333-2338.
16. W. Pu, D. Sun, W. Fan, W. Pan, Q. Chai, X. Wang and Y. Lv, *Chem. Commun.*, 2019, **55**, 4821-4824.
